# Some statistical properties of regulatory DNA sequences, and their use in predicting regulatory regions in the *Drosophila *genome: the fluffy-tail test

**DOI:** 10.1186/1471-2105-6-109

**Published:** 2005-04-27

**Authors:** Irina Abnizova, Rene te Boekhorst, Klaudia Walter, Walter R Gilks

**Affiliations:** 1 MRC Biostatistics Unit, Institute of Public Health, Robinson Way, Cambridge CB2 2SR, UK; 2 Computer Science Department, University of Hertfordshire, College Lane, AL10 92BA, Hatfield Campus, UK

## Abstract

**Background:**

This paper addresses the problem of recognising DNA cis-regulatory modules which are located far from genes. Experimental procedures for this are slow and costly, and computational methods are hard, because they lack positional information.

**Results:**

We present a novel statistical method, the "fluffy-tail test", to recognise regulatory DNA. We exploit one of the basic informational properties of regulatory DNA: abundance of over-represented transcription factor binding site (TFBS) motifs, although we do not look for specific TFBS motifs, *per se *. Though overrepresentation of TFBS motifs in regulatory DNA has been intensively exploited by many algorithms, it is still a difficult problem to distinguish regulatory from other genomic DNA.

**Conclusion:**

We show that, in the data used, our method is able to distinguish cis-regulatory modules by exploiting statistical differences between the probability distributions of similar words in regulatory and other DNA. The potential application of our method includes annotation of new genomic sequences and motif discovery.

## Background

The transcription rate of genes is dictated primarily by interactions between DNA-binding transcription factors. Comparatively short sequences (several hundred to several thousand base pairs, depending on thespecies) upstream or downstream of the transcription start site often play a major role in the regulation of gene expression. Specific sites within such regions are recognized by regulatory proteins (transcription factors), which act upon binding as transcriptional repressors or activators, controlling the rate of transcription. The identification of regulatory regions, which are generally composed of dense clusters of target transcription factor binding sites, forms an essential step in understanding the regulatory interactions that govern the spatial and temporal expression of individual genes (see for example [1,2]) and genetic regulatory networks, (see for example [3]).

Ultimately, this task is accomplished experimentally using techniques such as empirical deletion analysis, direct binding measurements, and co-precipitation of protein-DNA complexes. However, experimental verification is expensive and time consuming. Therefore, to address the growing volumes of available genomic sequence, a number of algorithms that identify putative cis-regulatory modules and transcription factor binding sites using evolutionary comparisons, whole-genome data, and known descriptions of transcription factor binding sites, have been successfully developed. Regulatory regions of higher eukaryotes can be subdivided into proximal regulatory units – promoters – which are located close to and upstream of the gene, and distal transcription regulatory units called enhancers or cis-regulatory modules. These may be located far upstream or downstream of the target gene, and are much more difficult to recognise. In our work we focus on recognition of enhancers.

Methods for recognising regulatory DNA may be divided into the following approaches:

1. Recognition of regulatory DNA regions based on description of known transcription factor binding sites (TFBS). This approach exploits the clustering of known, cooperatively-acting transcription factors (TFs). Extracting clustered recognition motifs is one of the most reliable techniques, but is limited to the recognition of similarly regulated cis-regulatory regions. Among the most popular representatives of search by known TFBS are [4-9].

2. Recognition of regulatory DNA based on phylogenetic foot-printing [10-14]. Methods of this type assume that regulatory regions are highly conserved in cross-genomic comparison, and conserved segments can be extracted from evolutionary related genomes. Performance of phylogenetic foot-printing depends on the evolutionary distance between given species and on the conservation level of individual genes. This is an actively progressing area, as more and more sequenced genomes appear. However, such an approach offers little information as to the specific function of the conserved sequences. Furthermore, it is still an open question as to how many genomes are sufficient for reliable extraction of regulatory regions.

3. Methods based on the difference of local nucleotide composition between regulatory and non regulatory DNA [15-18]. It is assumed that this difference is due to presence of multiple transcription signals, such as binding motifs for TFs in regulatory regions. The works [15-17] are based on constructing a global interpolated Markov model, applied to promoter recognition only.

In our method, we assume that the abundance of regulatory motifs within regulatory regions leaves a distinct "signature" in nucleotide composition, and that it is possible to capture this "signature" statistically. More specifically, we hypothesize that it takes the form of an over-representation of "similar words" (which are not simple repeats).

The approach of looking for over-occurrence of words has also been widely used in motif discovery, but this is not our aim here. This over-representation of similar words should appear as outliers in the right tail of the distribution of similar word lists of variable length. The "fluffy tail test", proposed in this paper, is designed to identify such outliers and is a useful technique when data from multiple genes and genomes are lacking. It may also be used as a complementary tool when such data are available.

## Results

In this section, we first present our new statistical 'fluffy tail' test for measuring the overrepresentation of similar words, and then show its performance on experimentally verified sequence data.

### Test bed

To demonstrate the power of our test, we need a positive, experimentally verified, training set of regulatory sequence data, and also negative training sets of non- regulatory sequence data. We use three test beds. The positive training set is a collection of 60 experimentally verified functional *Drosophila melanogaster *regulatory regions [18]. This set consists of cis-regulatory modules located far from gene coding sequences and transcription start sites. It contains many binding sites (and site clusters), best known of which are bicoid, hunchback, Kruppel, knirps and caudal, – the sites involved in the regulation of developmental genes. The total size of the positive training set comprises about 68 Kb of sequence data, and contains 58 clusters of the same type of TFBS (homotypic). The two negative training sets are: (i) 60 randomly picked *Drosophila *exons, and (ii) 60 randomly picked *Drosophila *non-coding, non-regulatory DNA sequences: we excluded exons and regions of length 1 KB upstream and downstream of genes, using the Ensembl Genome Browser [19]. Each training set contains 68 Kb of sequences in total.

### Estimation of distributions of similar words

**Figure 1:**
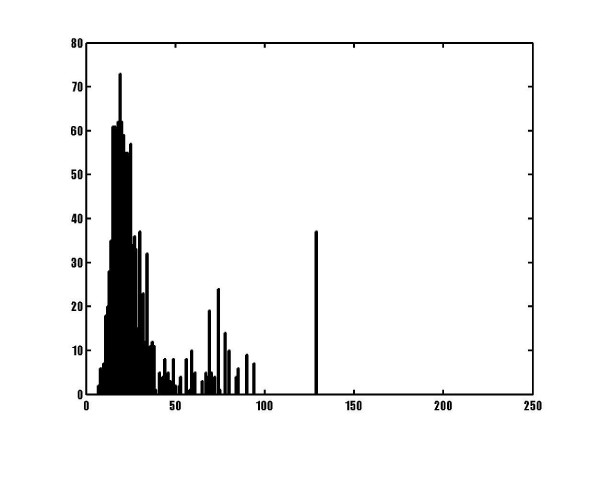
**Histogram of similar words for the knirps cis-regulatory module**. An example of a distribution of similar 5-mer words for the knirps cis-regulatory module *Drosophila melanogaster *. Note that the sequence contains an exceptionally large number (37) of lists with an exceptionally large number (137) of similar words. The Y axis shows the number of lists, the X axis is for list size.

To construct the distribution of similar words, we first need to specify the length of words under consideration. We try to mimic the TF core, which is the less variable part of a binding motif. Because the core of TFBSs is relatively short (around 3–5 bp) we considered 5-mer words, allowing for 1 mismatch. However, our results also hold for words of length 4 through 12, allowing for 1 through 4 mismatches (see Supplementary Materials [see Additional files 1, 2, 3, 4, 5, 6, 7, 8, 9, 10, 11, 12]). Thus, for each 5-mer word in each of the 180 sequences (60 sequences in each training set) we computed the number n of similar words of the same length. Thus, each word is the "seed" of a list of similar words. Next, the number of (non-disjoint) lists containing n words is counted, where n = 1,2,3....

(See Methods section for further details). As an example, thehistogram of the distribution of similar 5-mer words is plotted in Figure 1. In this histogram, the Y axis represents the number of lists containing 1,2, ..., n words and the X axis shows the number n of similar words in the list.

From this plot it can be seen that most lists contain 10 to 40 words, but there are outliers: some very large lists form a long, "fluffy" tail. We call a list having the largest size the maximal similar word list (MSWL). If the original sequence is characterized by the presence of an unusually high number of over-represented words, we expect it to contain more long lists in comparison to a random sequence.

**Figure 2:**
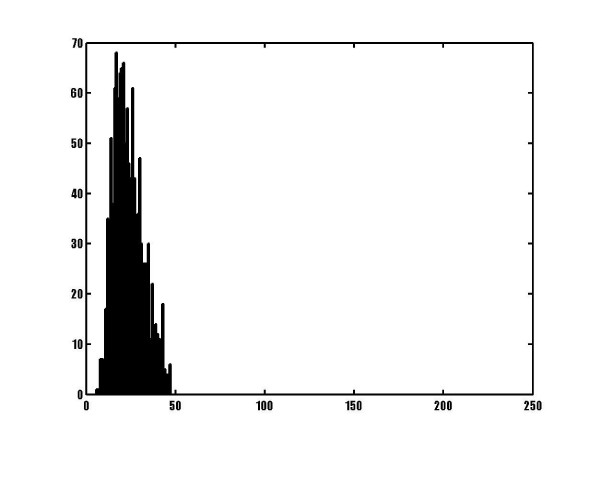
**Histogram of similar words for the knirps cis-regulatory module, after shuffling**. The frequency distribution of similar words for one randomly shuffled version of the knirps cis-regulatory region, ***Drosophila melanogaster ***. The Y axis shows the number of lists, the X axis is for list size.

**Figure 3:**
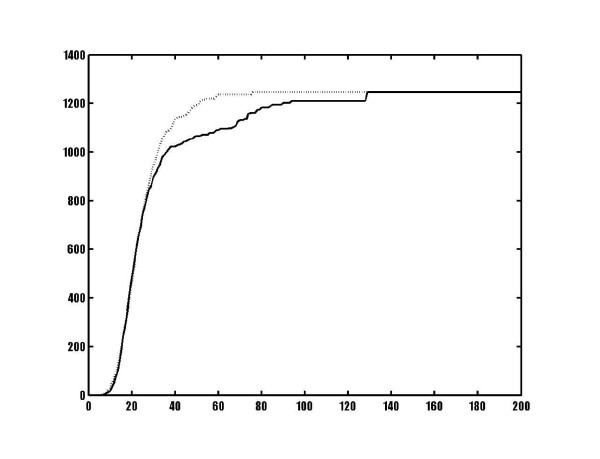
**Cumulative histograms**. Cumulative histograms for the data in Figures 1 and 2: solid line: original data from Figure 1, dotted line: randomised data from Figure 2. The X axis shows the size of lists of similar words, the Y axis is the number of lists.

To sample such a random distribution we shuffled the given sequence of original data 50 times. For each randomisation we assessed the frequency distribution of similar words. Figure 2 shows a typical example of the distribution of similar words for one of the randomly shuffled sequences of the same (knirps) cis-regulatory module as in Figure 1. Compared with the distribution of the original data (Figure1), the randomised sequence in Figure 2 lacks a heavy, "fluffy" right tail. Figure 3 shows the difference between original and randomised similar word distributions in cumulative form. The difference between the two curves reflects the fluffy right tail of the original data.

**Figure 4:**
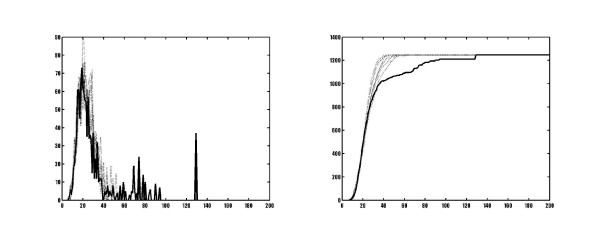
**Fluffy-tailed knirps distribution**. (Left) The distribution of the original regulatory knirps sequence: (solid line); the distribution of 10 randomised sequences (dotted lines). (Right) The same distributions in cumulative form. The X axis shows the size of lists of similar words, the Y axis is the number of lists.

In Figure 4, ten randomised sequences are plotted as dotted contours together with the histogram of the original regulatory knirps data (solid). The cumulative histogram for original (solid) and randomised (dotted) sequences is shown in Figure 4 (right). All dotted tails are shorter than the solid one, indicating the statistical significance of the solid tail.

### Definition of the fluffiness coefficient F

To measure how strong the distribution of similar words of regulatory regions deviate from randomness, we introduce a "fluffiness" coefficient F:

)

*w *here *L *_max,*original *_is the number of words in the maximal similar word list (MSWL) in the original sequence,  and σ_*r *_are the mean and standard deviation of the MSWL size in each of *r *shuffled sequences. Here we call the sequence "random" if it is obtained from original sequence by shuffling it, preserving its single nucleotide composition. We will omit the subscript *r *for *F*_*r *_later in the paper for simplicity.

One can regard F as measuring the difference between signal and noise, where the signal is taken from the original sequence, and the noise from the randomised sequences with the same composition and length. Thus, the fluffiness coefficient is normalised for the length and base composition of the sequence, because we compare each original sequence only with respect to shuffled sequences of the same length and composition. Thus one can compare the fluffiness F for sequences of different base composition and length.

### Results for regulatory regions

**Figure 5:**
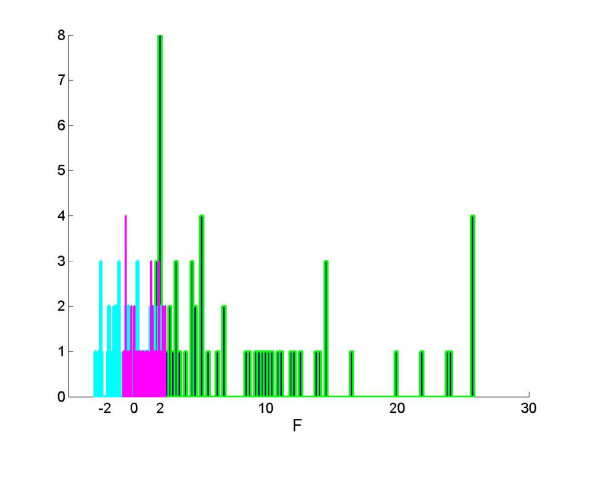
**Histograms for regulatory (green), coding (cyan) and NCNR (magenta) sequences**. The word length is 5, mismatch is 1, r is 50. The X axis shows the fluffiness coefficient F, the Y axis is the number of sequences in the set with this F.

Figure 5 shows the distribution of fluffiness coefficient F for regulatory, coding and non-coding non-regulatory (NCNR) DNA. In each sequence we generated r = 50 shuffled versions, in calculating F. One can see that F = 2 distinguishes regulatory DNA from other types of DNA. Thus, we use the value F = 2 as a threshold. A sequence with F>2 we declare to have a "fluffy" tail. Moreover, we found that for each regulatory region having F>2, all the randomised sequences had a shorter tail. This value F = 2 is sufficiently robust: if we vary our threshold a little around F = 2, we still get a fair separation.

Our choice of r = 50 shuffled versions for each sequence allows us to obtain reliable estimates for the fluffiness coefficient F and make the computational time reasonable. Table 1 shows that F is somewhat unstable for smaller r for the knirps regulatory region. However, for each choice of r, F clearly exceeds the threshold value 2, in this example. See Supplementary Materials for more detailed descriptions [see Additional files 1, 2, 3, 4, 5, 6, 7, 8, 9, 10, 11, 12].

Using the methodology described above, we found that 51 out of 60 regulatory regions (85%) analysed in our positive training set exhibit the significant "fluffy-tail" pattern (see Table 2). The non-detection of the remaining "non-fluffy" regulatory regions could perhaps be partly due to the limited power of experimental deletion analyses to correctly distinguish the boundaries of the cis-regulatory modules.

**Figure 6:**
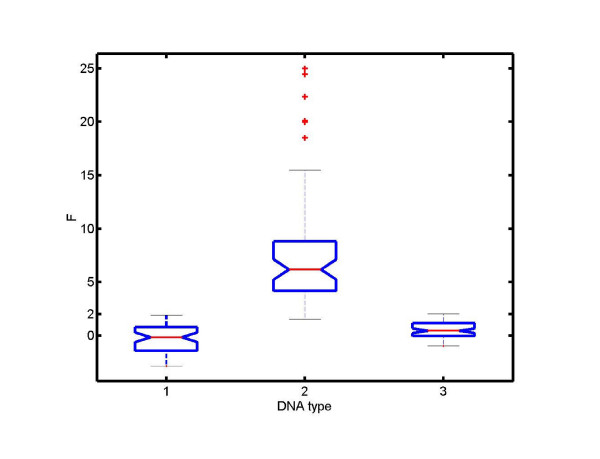
**Separation of regulatory DNA**. Separation of regulatory DNA (column 2) from coding (column 1) and non-coding, non-regulatory (column 3) due to the fluffiness coefficient F (Y-axis). Box-plot of the Fluffiness (Y-axis) index for the three functional regions.

We calculated the distribution of F for our two negative and one positive training sets. The separation of regulatory DNA from coding and non-coding, non-regulatory DNA on the basis of fluffiness was quantified by estimating the distribution of the F coefficients. A Kruskal-Wallis test showed that these regions differ significantly in the magnitude of the fluffiness coefficient (H = 132.81, N = 180, df = 2, p = 0.00001), with exons and non-coding non-regulatory DNA having much lower F-values than regulatory regions (See Fig. 6).

We now turn to examine the location of similar words in the MSWL for a given sequence.

**Figure 7:**
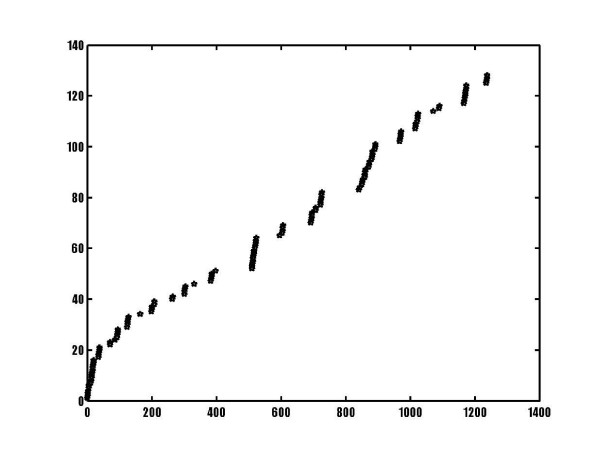
**Spatial distribution of similar words in MSW L**. **F**airly uniform spatial distribution of start locations for words in the MSWL (n = 137, see Fig.1) of the knirps cis- regulatory region of *Drosophila melanogaster *. The X axis shows the positions of each word start in the sequence, the Y axis is the rank of this position in the list.

When the start positions of each of the words in the MSWL are plotted, they tend to be fairly uniformly scattered along the length of the sequence, as illustrated in Figure 7.

We now examine the relationship between the MSWL and predicted TFBS sites. We found significant enrichment of most MSWLs with the occurrences of TFBS in databases: when submitted to the Transfac and Jaspar TFBS databases, the "seed" words for MSWLs exhibited 10–20 fold enrichment with putative TFBS in comparison with all 5-mer words within the given regulatory region: thus, for the most part, these "seed" words turned out to be instances of known TFBS (results not shown here).

### Results for exons

**Figure 8:**
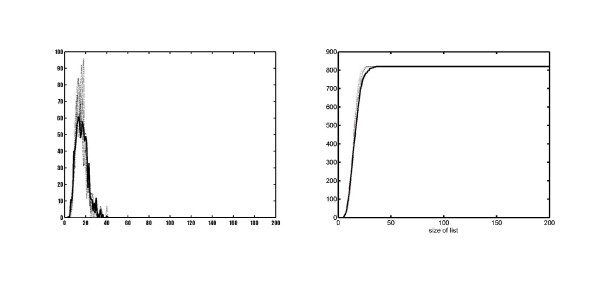
**Histogram for exon *cg3201 *3**. Distribution of similar words for the exon *cg3201 *3 of *Drosophil *a (solid line) compared to the histograms of the randomly shuffled versions (dotted lines) in direct (left) and cumulative (right) forms. The X axis shows the size of lists of similar words, the Y axis is the number of lists.

We repeated the fluffy tail test for randomly picked *Drosophila *exons, and found that the distribution of over-represented words of the original sequences did not differ statistically from those of their randomised versions (See Table 2). Note the absence of a "fluffy tail" in Figure 8 (left) and the lack of distinction in the cumulative distribution (Figure 8 right).

Thus we have established a statistical difference between exons and regulatory DNA. Next we compare regulatory DNA with non-coding non-regulatory DNA.

### Results for non-coding, presumed non-regulatory DNA

**Figure 9:**
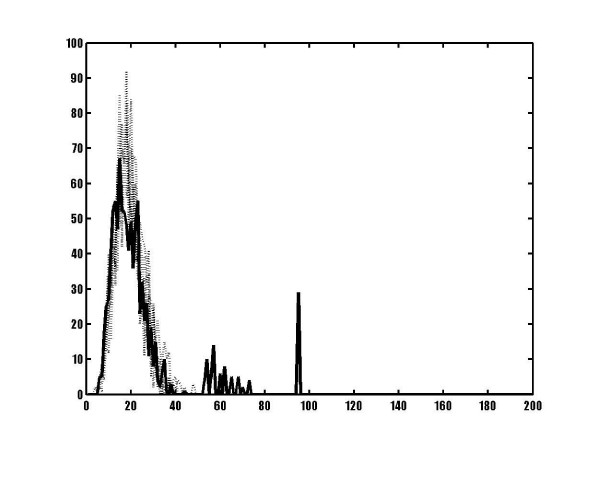
**Histogram for non-coding presumed non-regulatory sequence**. Distribution of similar words for a non-coding, non-regulatory sequence, randomly picked from chromosome 3L has significant tail because of simple repeats. The X axis shows the size of lists of similar words, the Y axis is the number of lists.

The similar words distribution for non-coding non-regulatory DNA typically shows two patterns: (1) without significant tails, as for exons and (2) with significant tails (Figure 9) but in this case – and in contrast to the regulatory sequences – the spatial locations of over-represented words are typically clustered (Figure 11c).

To deal with this, we developed a measure of spatial clustering of similar words. We say that two words *w *_1 _and *w *_2 _belong to the same cluster, if their genomic start positions *s *_1 _and *s *_2 _satisfy |*s *_1 _- *s *_2_| ≤ *m*·*k *, where m is the word length, and k is a constant. We examined the following choices for k: 1; 1.5; 2; 2.5; 3.

**Figure 10:**
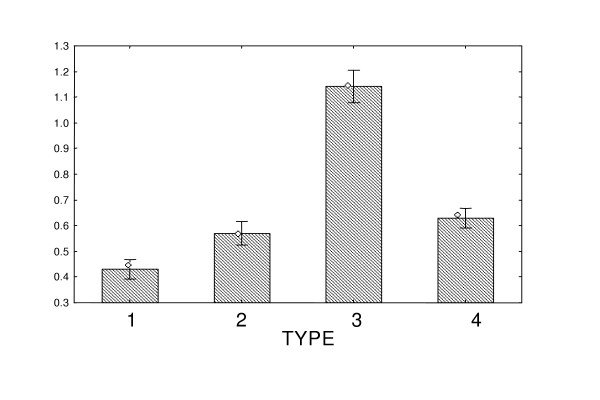
**Coefficient of variation in spatial cluster size for four types of DNA: **exons (1), non-fluffy NCNR (2), fluffy NCNR (3), regulatory regions (4); Vertical bars denote 95% confidence intervals. The Y axis shows coefficient of variation, the X axis is for four DNA type. We calculated CV based on spatial clustering coefficient k = 1.

The size of a cluster is defined as the number of words in the cluster. For each MSWL we computed the coefficient of variation (CV) in cluster sizes, where CV is the standard deviation divided by the mean cluster size. We used analysis of variance to test for difference in coefficients of variance among four types of functional DNA: exons, non-fluffy NCNR, fluffy NCNR and regulatory regions. The assumptions for ANOVA (homogeneity of variance (CV), no correlation between means and standard deviations of the samples) were satisfied. The results show a strongly significant difference between the four types: see Figure 10. Thus we can use the cluster size CV to distinguish fluffy NCNR from regulatory DNA. CVs for fluffy NCNR are almost always more than 1, for k from 1 to 3; and significantly different from CVs for regulatory DNA.

**Figure 11:**
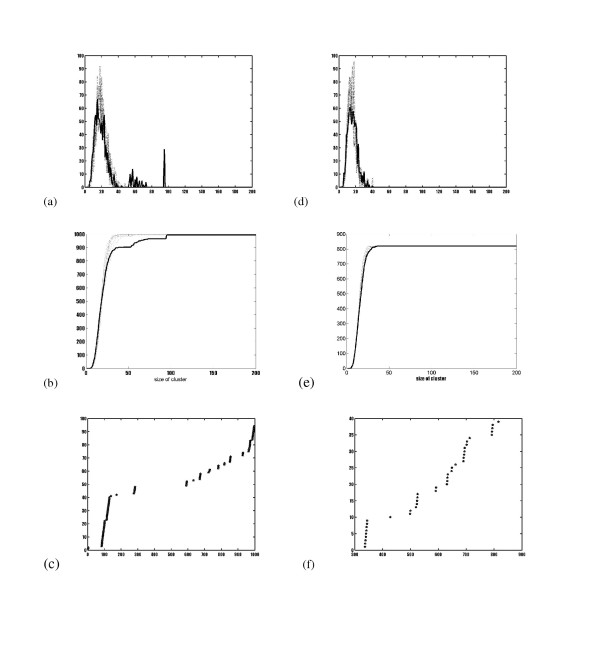
**Non-coding presumed non-regulatory sequence before and after repeat-masking**. For a non-coding, non-regulatory sequence, randomly picked from chromosome 3L. Panels (a,b,c) show results before repeat-masking; panels (d,e,f) show results after repeat-masking. Panels (a,d) show histograms of similar words (solid: original data; dotted: after random shuffling) as in Figure 1; panels (b,e) show the same data in cumulative form as in Figure 3; panels (c,f) show start locations of similar words as in Figure 7.

We found that large clusters of adjacent over-represented words in fluffy NCNR DNA disappear after repeat-masking [20], thus revealing their identity as non-perfect simple repeats (Figure 11: compare panels a,b,c with d,e,f).

For details about spatial clustering and illustration of coefficient of variation robustness to choice of **k **and **m**, see Supplementary Materials [see Additional files 1, 2, 3, 4, 5, 6, 7, 8, 9, 10, 11, 12].

## Discussion

Our method allows us to distinguish regulatory DNA from other non-regulatory DNA. In effect, our method aggregates many small signals contained in the region, and makes an internal comparison with background, represented by shuffled sequences.

We would like to extend the application of our method to larger sets of experimentally verified regulatory regions, from *Drosophila *or any other species. Unfortunately, few experimentally (not computationally!) verified sets are available. We managed to extended our positive training set a little, including a few experimentally verified regulatory regions from human, chicken, sea urchin, fruit fly and yeast (see Supplementary Materials [see Additional files 1, 2, 3, 4, 5, 6, 7, 8, 9, 10, 11, 12]), but it is still not a lot.

We would also like to explore the correlation between the genomic positions of words in MSWL (most abundant words), and positions of known regulatory elements. This may allow us to utilise our method as a kind of motif discovery algorithm. Unfortunately, again, the lack of reliably annotated regulatory regions with regulatory elements makes this step difficult.

Phylogenetic foot-printing is an important and rapidly developing branch of motif discovery methodology. It would be very interesting to compare genomic positions of words in MSWL with conserved sequences from phylogenetic foot-printing analyses. This would reveal whether such words are conserved, and therefore of functional significance.

In a similar vein, we would like to compare the results of fluffiness analysis results across multiple species. We could then answer the question whether cross-species conserved regions have "fluffy" regulatory region properties, and thus infer their putative function.

We are keen to compare results of our fluffy-tail-analysis with the results of recognition methods based on description of known TFBS, such as in the works [6] and [4]. These authors [4] also analysed developmental genes of *Drosophila melanogaster *containing approximately the same clusters of transcription factors.

The work [18] is closely related to our study. However, it is likely that their method is unable to distinguish non-perfect simple tandem repeat sequences from truly regulatory DNA. We have implemented their method as far as we can understand it, and found out that their separation of positive (cis-regulatory modules) and negative (coding and non-coding non-regulatory DNA) training sets due to local words frequency seems to be less clear than our separation due to "fluffiness" coefficient F (see Figure 6).

There might be possible other regulatory mechanisms apart from TFBS binding. It may be in some specific cases that the 3D local structure of DNA in the nucleus (chromatin) is the principal factor of gene expression and modulating regulatory modules play little or no role [21]. Thus one of the next steps in our work will be the incorporation of nucleosome position information.

## Conclusion

We present a novel statistical approach that allows regulatory DNA to be distinguished from coding and non-coding non-regulatory regions according to its "fluffiness" values. This method is based on the presence of unusually high number of short runs of over-represented scattered words in the given DNA sequence.

The performance of the method on experimentally verified sequence data shows that the method allows us to predict whether a sequence may be regulatory.

## Methods

### Description of fluffy tail test

The fluffy tail test essentially consists of the comparison of similar word distributions for the original sequence and for a number of shuffled versions of the original sequences. These shuffled sequences clearly have the same length and single nucleotide composition as the original one.

To construct a similar words distribution one can perform the following two steps:

(1). First, obtain the distribution of similar words for a given DNA stretch (as described in detail below under "Distribution of similar words"). Then randomise the original sequence many times, and obtain a distribution of similar words for each shuffled sequence. These randomised sequences represent the null model (or the background model). The distributions of similar words obtained for the randomised sequences are compared with the corresponding distribution for the original sequence. If there are no statistical differences, we conclude that the sequence probably is an exon (related results are in [22]) or a homogeneous non-coding non-regulatory region.

However, if the given sequence does contain many similar words, these will show up in its distribution as a longer right tail that may even have a second mode. Such "fluffy" tails are seldom found in the distributions of the shuffled sequences, therefore suggesting the sequence is not exonic or homogeneous non-coding, non-regulatory DNA.

(2). To rule out "fluffy" tails due to non perfect simple tandem repeats, we check whether a) the similar words are spatially clustered and b) if the tails disappear after repeat-masking the sequence (using the on-line tool available at [20]) then repeating procedure (1).

### Distribution of similar words

We considered 5-mer words, allowing for 1 mismatch. However, our results also hold for words of length 4 through 12, allowing for 1 through 4 mismatches (see Supplementary materials [see Additional files 1, 2, 3, 4, 5, 6, 7, 8, 9, 10, 11, 12]). Thus, for each 5-mer word in each of the 180 sequences (60 sequences in each training set) we computed the number n of similar words of the same length. Each word is the "seed" for a list of similar words.

As an example, consider a stretch of DNA :

accgggtgtaaaccgacctgatacccggtcgcccggttttaac...

The first "seed" 5-word 'accgg' forms the following list of similar words:

accgg, accga, acctg, acccg, cccgg,

which we have underscored in the above sequence.

The second 5-word 'ccggg' forms another list of similar words:

ccggg, ccggt, ccggt

etc. The first 5-word has the longest list of similar words here. The lists may intersect: e.g. the list for the 'accga'- seed word contains some words from the 'accgg'-seed word list.

## Authors' contributions

WRG contributed to development of methodology, KW did numerical comparison with other related methods, RtB statistically processed the data, IA contributed to development of methodology, collected the data and wrote the software. All authors read and approved the final manuscript

## Supplementary Material

Additional File 1 Contains short introduction and notation for Supplementary Material Click here for file

Additional File 2 Contains Supplementary Table1 with results of Fluffy-tail test and Coefficients of Variation for some more experimentally verified regulatory regions for other than Fruit fly species. Click here for file

Additional File 3 Contains a visual example of F dependence on the number of randomisations r. Click here for file

Additional File 4 Gives some more details about spatial clustering threshold Click here for file

Additional File 5 Shows some examples for consistence of fluffiness for different word length, tables. Click here for file

Additional File 6 Shows some examples for consistence of fluffiness for different word length in the histogram form Click here for file

Additional File 7 Consistent fluffiness and coefficient of variation for spatial cluster size for some example sequences. Click here for file

Additional File 8 Contains the Figures showing fluffiness and spatial clustering of similar words for NCNR 3L4 region. Click here for file

Additional File 9 Contains the Figures showing fluffiness and spatial clustering of similar words for NCNR repeat-masked 3L4 region. Click here for file

Additional File 10 Contains the Figures showing fluffiness and spatial clustering of similar words for knirps regulatory region Click here for file

Additional File 11 Contains the Figures showing fluffiness and spatial clustering of similar words for abdominantA regulatory region. Click here for file

Additional File 12 Contains the Figures showing fluffiness and spatial clustering of similar words for internal exon 2r4. Click here for file

## Figures and Tables

**Table 1:** Sensitivity of F to choice of r, the number of randomisations, for the knirps regulatory region.

r	F	σ_*r *_
25	14.7	5.39
50	8.65	8.77
100	10.22	7.56

**Table 2:** "Fluffiness" predictions for three types of functional region, showing the number of fluffy (F>2) sequences, the number of non-fluffy (F<2) sequences and corresponding positive and negative prediction rates, for each type of the region.

**Functional type**	**Fluffy tails (F>2)**	**No fluffy tails (F<2)**	**Positive rate**	**Negative rate**
Regulatory regions	51	9	85 %	15 %
Exons	1	59	1.6 %	98.4 %
Non-coding presumed non- regulatory	10	50	16 %	84 %
