# Supplementary material for: Some statistical properties of regulatory DNA sequences, and their use in predicting regulatory regions in the Drosophila genome: the fluffy-tail test
Source: BMC Bioinformatics. 2005 Apr 27;6:109. doi: 10.1186/1471-2105-6-109 (PMC1127108; doi:10.1186/1471-2105-6-109)
Supplement: Additional File 1 — Contains short introduction and notation for Supplementary Material [file 1471-2105-6-109-S1.doc]

# Supplementary Materials to the manuscript 'Some statistical properties of regulatory DNA sequences, and their use in predicting regulatory regions in the Drosophila genome: the fluffy-tail test.' *Irina Abnizova, Klaudia Walter, Rene te Boekhorst and Walter R. Gilks*

# Supplementary introduction and notations

In this section we present the evidence that our main result, namely our ability to distinguish regulatory DNA from coding DNA and non-coding non-regulatory DNA, does not depend strongly on the size of words examined, and is valid for words of length m=3,5,7,9,12 with corresponding mismatches=0,1,2,3,4.

We show that when word length is increased, regulatory regions still remain fluffy ( F>2), exons and masked non-coding and non-regulatory DNA remain un-fluffy, while the coefficient of variation (CV) of spatial cluster size for fluffy (not masked) non-coding and non-regulatory DNA remains high (>1.0). We show exhaustive examples of variability of F and CV for the abdominant Anterior regulatory region, knirps regulatory region, random DNA from chromosome 3L (3L4) and internal exon CG10392 (exon 2r4) from chromosome 2R: see corresponding Supplementary files.

Notation used:

Similarity=(m,mim)= e.g. (5,1) where

m = length of the word

mim= number of allowed mismatches

F = **coefficient of fluffiness** ( measure of similar words abundance in comparison

with background model). It is the number of standard deviations over the

mean:

$F=(L_{\max,origina}-\bar{L})/\sigma$

*w*here L_max, original_ is the number of similar words in the largest list in the original sequence, $\bar{L}$ is computed as

$$\bar{L}=\frac{\sum_{i=1}^{r} L_{\max,i}}{r}$$

where L_max,i_ is the sizes of largest clusters in the i-th shuffled sequence, $\sigma$ is their standard deviation, and $r$ is the number of randomisations.

If F>2 we call the sequence “fluffy”:

CV = **coefficient of variation** in the size of clusters of adjacent similar words in MSWL (maximal similar words list): measure of scattering of these values
