# Supplementary material for: Some statistical properties of regulatory DNA sequences, and their use in predicting regulatory regions in the Drosophila genome: the fluffy-tail test
Source: BMC Bioinformatics. 2005 Apr 27;6:109. doi: 10.1186/1471-2105-6-109 (PMC1127108; doi:10.1186/1471-2105-6-109)
Supplement: Additional File 2 — Contains Supplementary Table1 with results of Fluffy-tail test and Coefficients of Variation for some more experimentally verified regulatory regions for other than Fruit fly species. [file 1471-2105-6-109-S2.doc]

# Supplementary Materials to the manuscript 'Some statistical properties of regulatory DNA sequences, and their use in predicting regulatory regions in the Drosophila genome: the fluffy-tail test.' *Irina Abnizova, Klaudia Walter, Rene te Boekhorst and Walter R. Gilks*

**Supplementary Table1**: Results of Fluffy-tail test and Coefficients of Variation for some more experimentally verified regulatory regions for other than Fruit fly species.

| species | Sequences name | F | CV |
| --- | --- | --- | --- |
| human | hoxb | 6.3 | 0.79 |
| human | bah | 4.03 | 0.90 |
| human | hoxa | 8.0 | 0.86 |
| chicken | bach | 4.05 | 0.85 |
| Common carp | bac | 5.95 | 0.90 |
| Fruit fly | Ultr1 | 4.09 | 0.70 |
| Fruit fly | Even3 | 5.8 | 0.56 |
| Yeast | Ade5 | 2.1 | 0.80 |
| Yeast | Cln1 | 7.0 | 0.53 |
| Yeast | Pgk | 2.01 | 0.87 |
| Yeast | Cln3 | 2.23 | 0.80 |
| Yeast | Gal10 | 2.32 | 0.86 |
| Yeast | Car1 | 3.02 | 0.66 |
| Yeast | Cdc19 | 8.4 | 0.93 |
| Sea urchin | cyiiia | 3.7 | 0.59 |

**Legend for Supplementary Table1**: First column shows the species type of the sequence. Third column shows Fluffiness for each regulatory sequence (second column), fourth column shows Coefficient of Variation (CV) for each regulatory sequence.

Note, that Fluffiness F is always more than 2 ( fluffy) and CV<1 (comparable cluster sizes of similar words), which was typical for our Drosophila enhancers positive training set.

Below are some visualizations of Fluffiness and spatial clustering for some regulatory sequences from the **Supplementary Table1**:


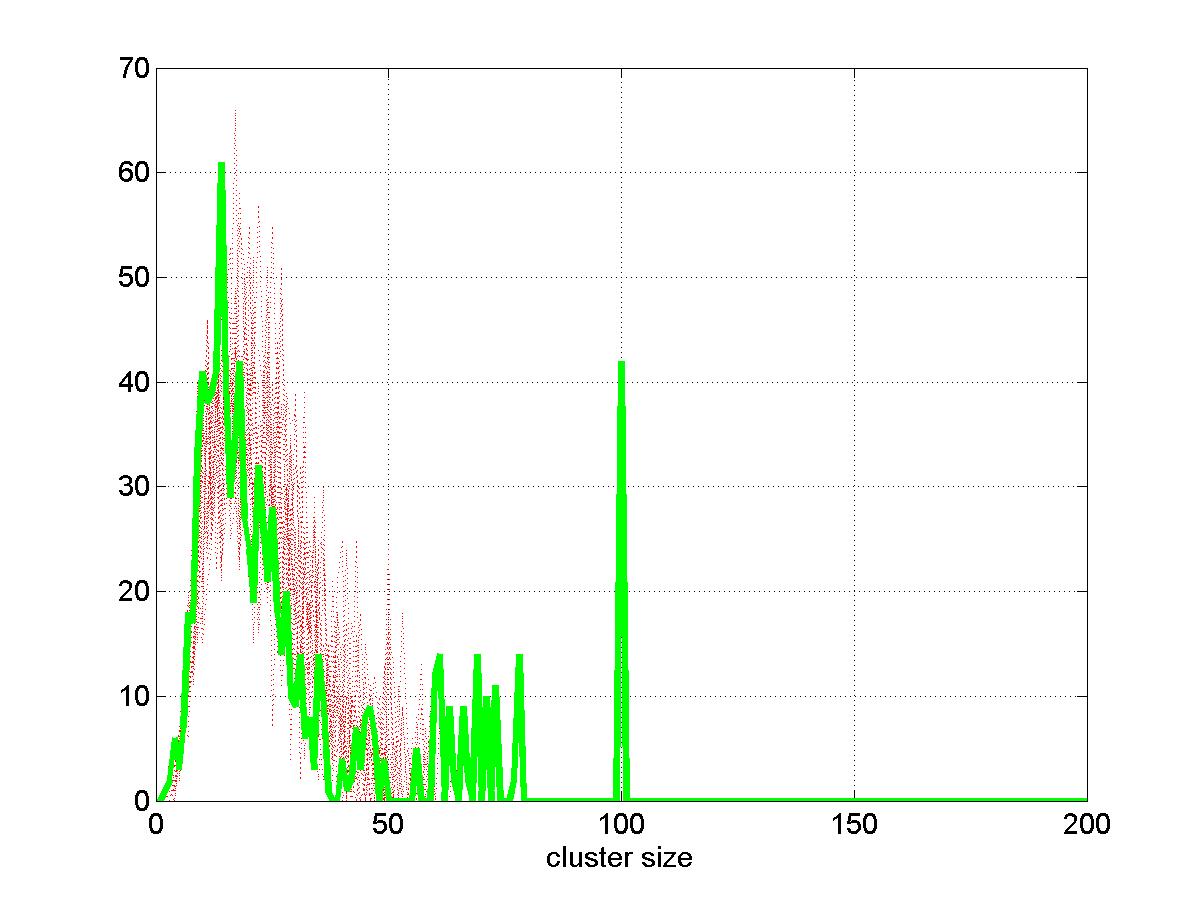

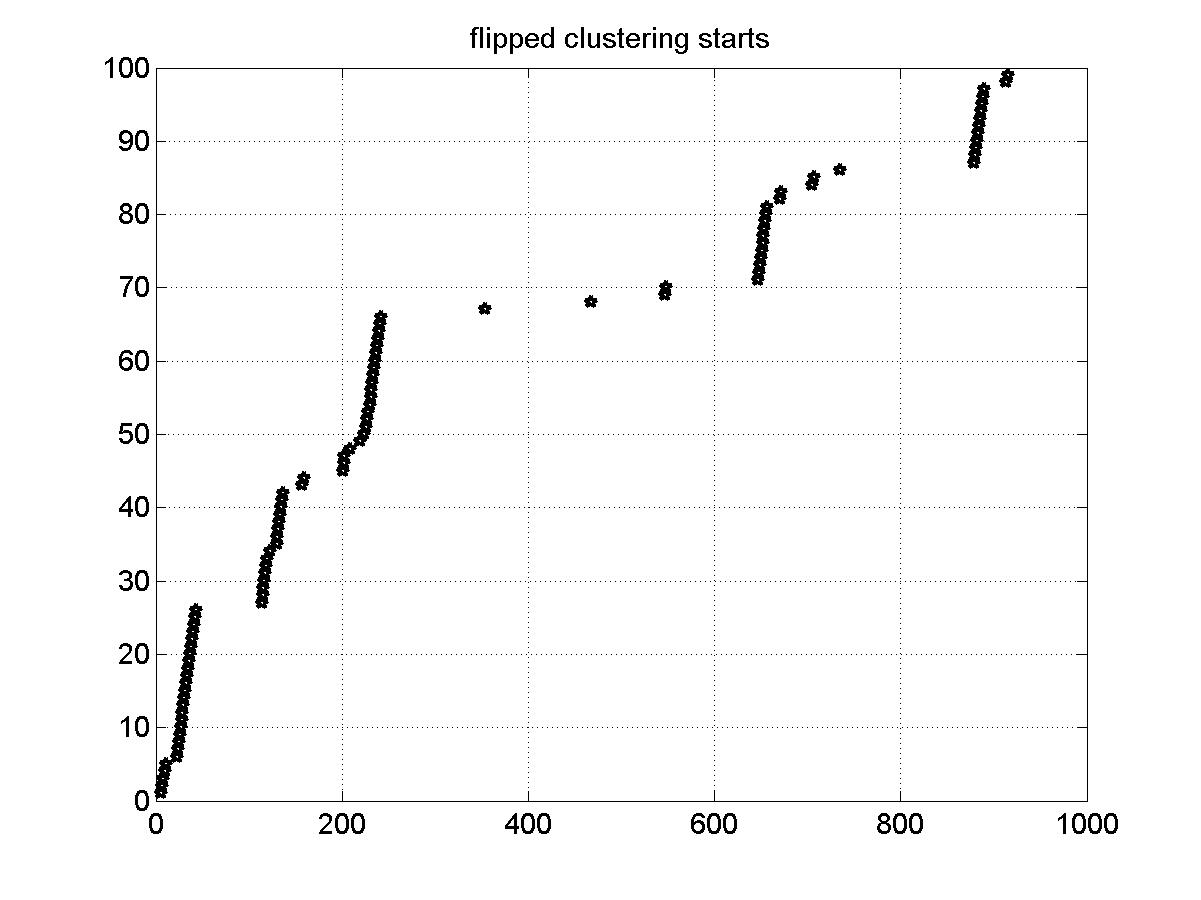


**Figure S1: Similar word distribution and spatial clustering for Cdc19 regulatory region, yeast.**


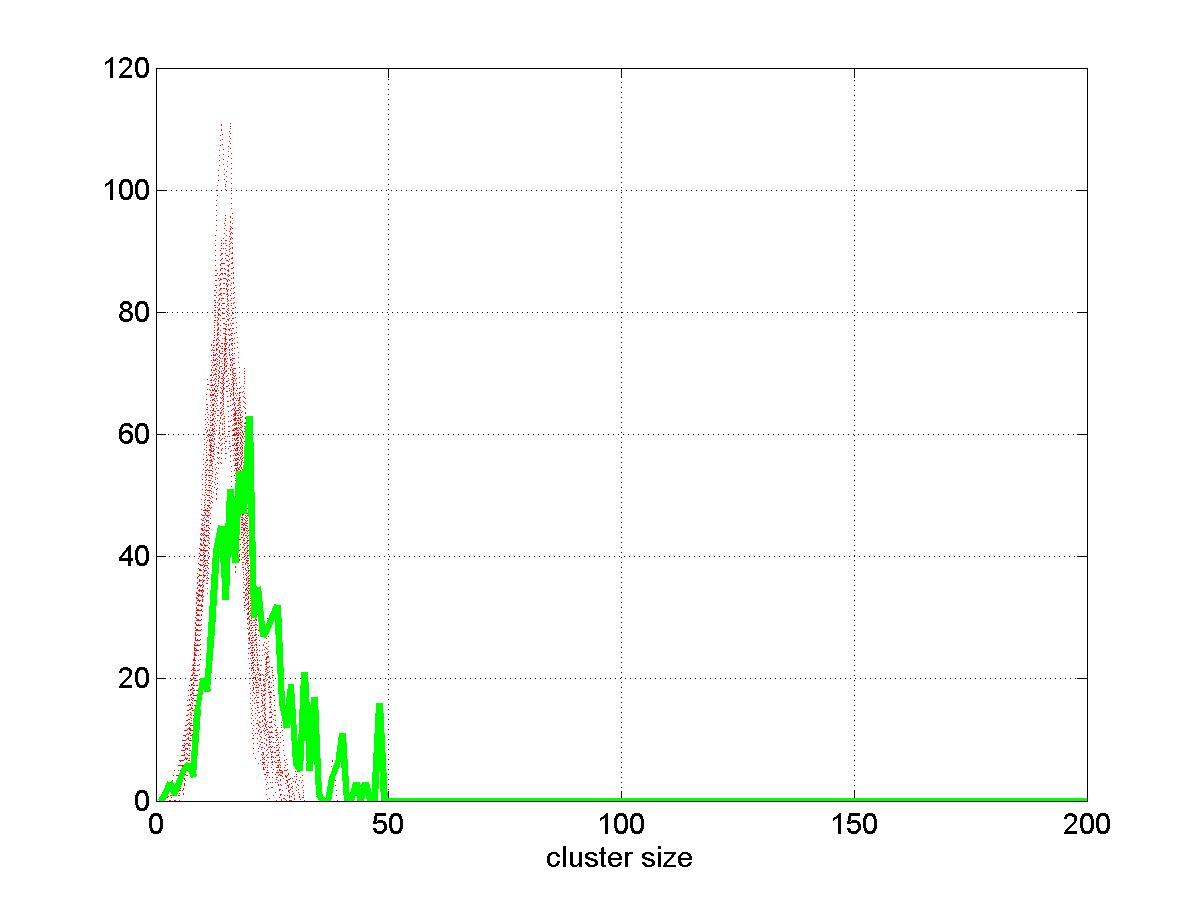

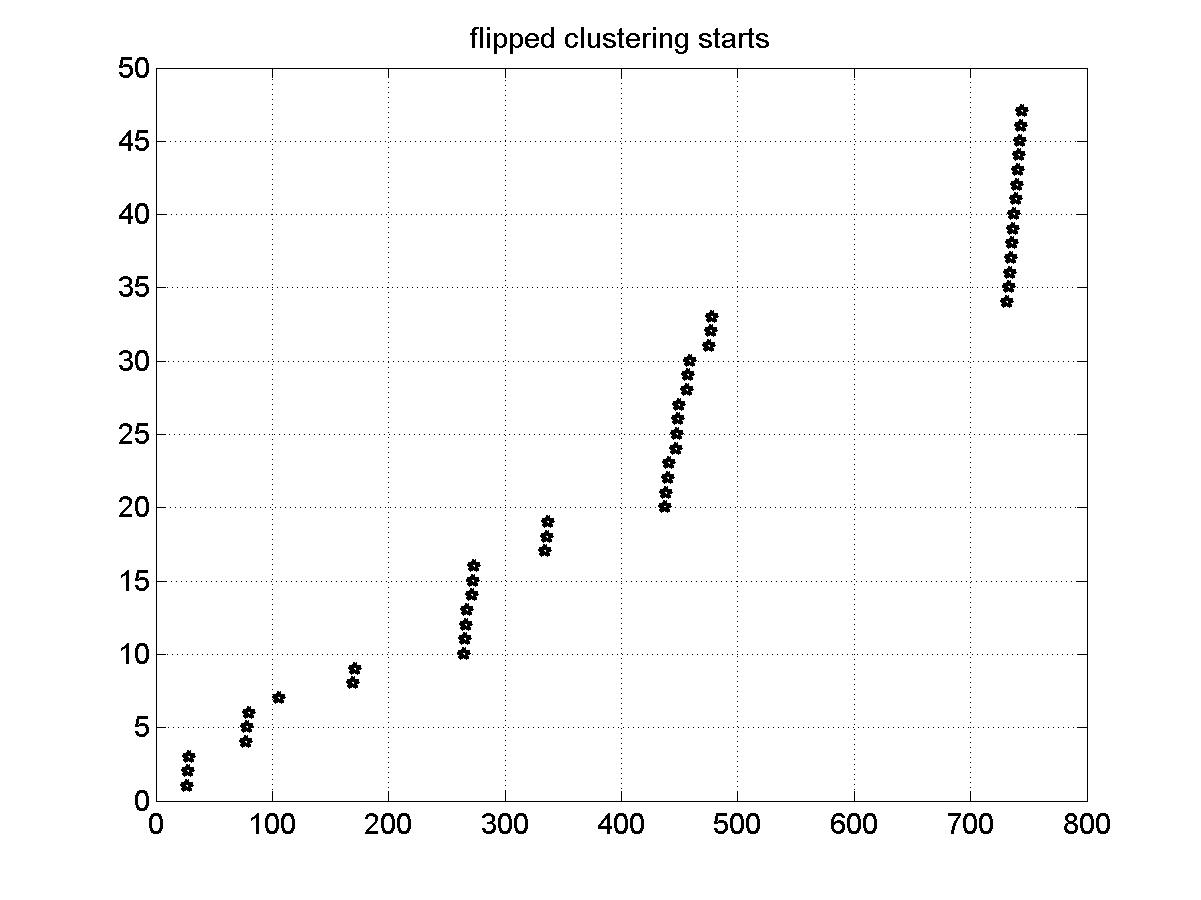


**Figure S2: Similar word distribution and spatial clustering for hoxa regulatory region, human.**


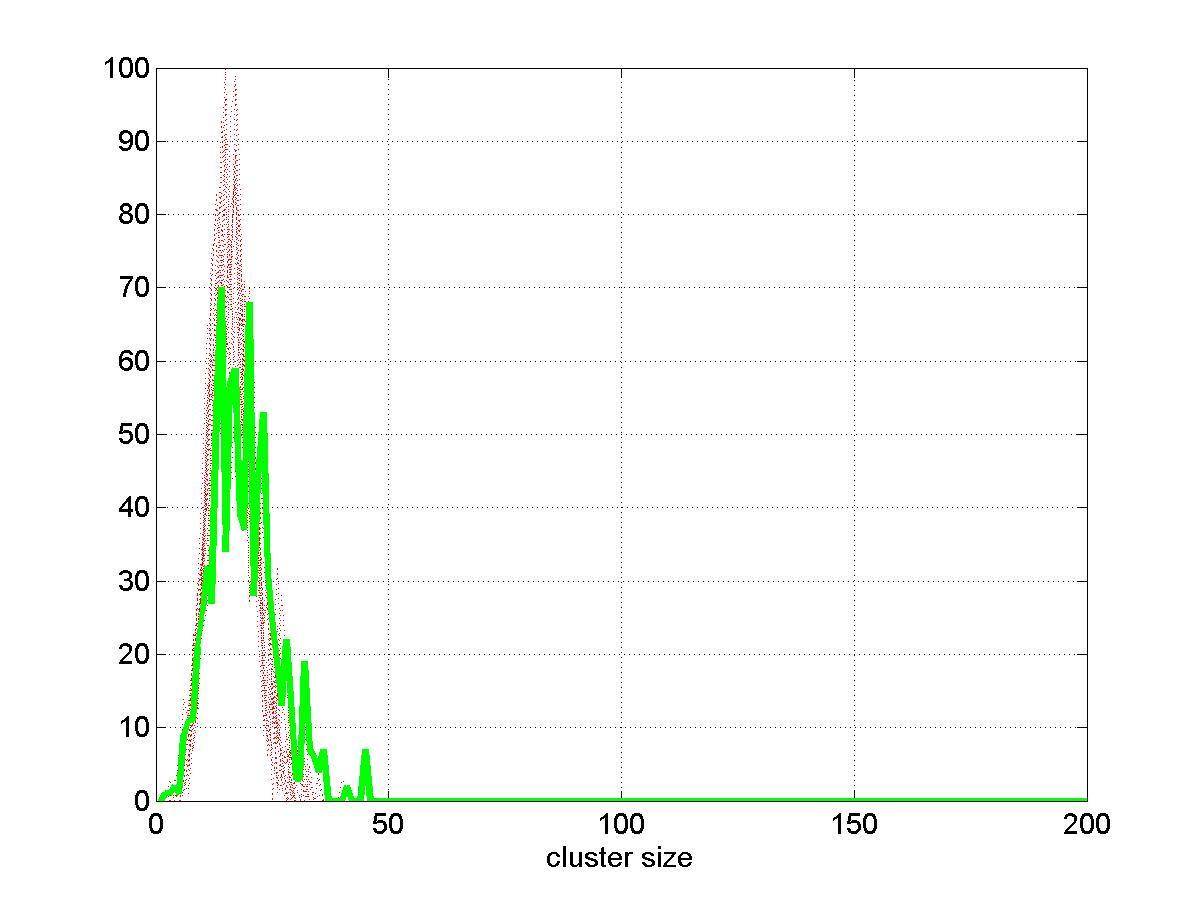

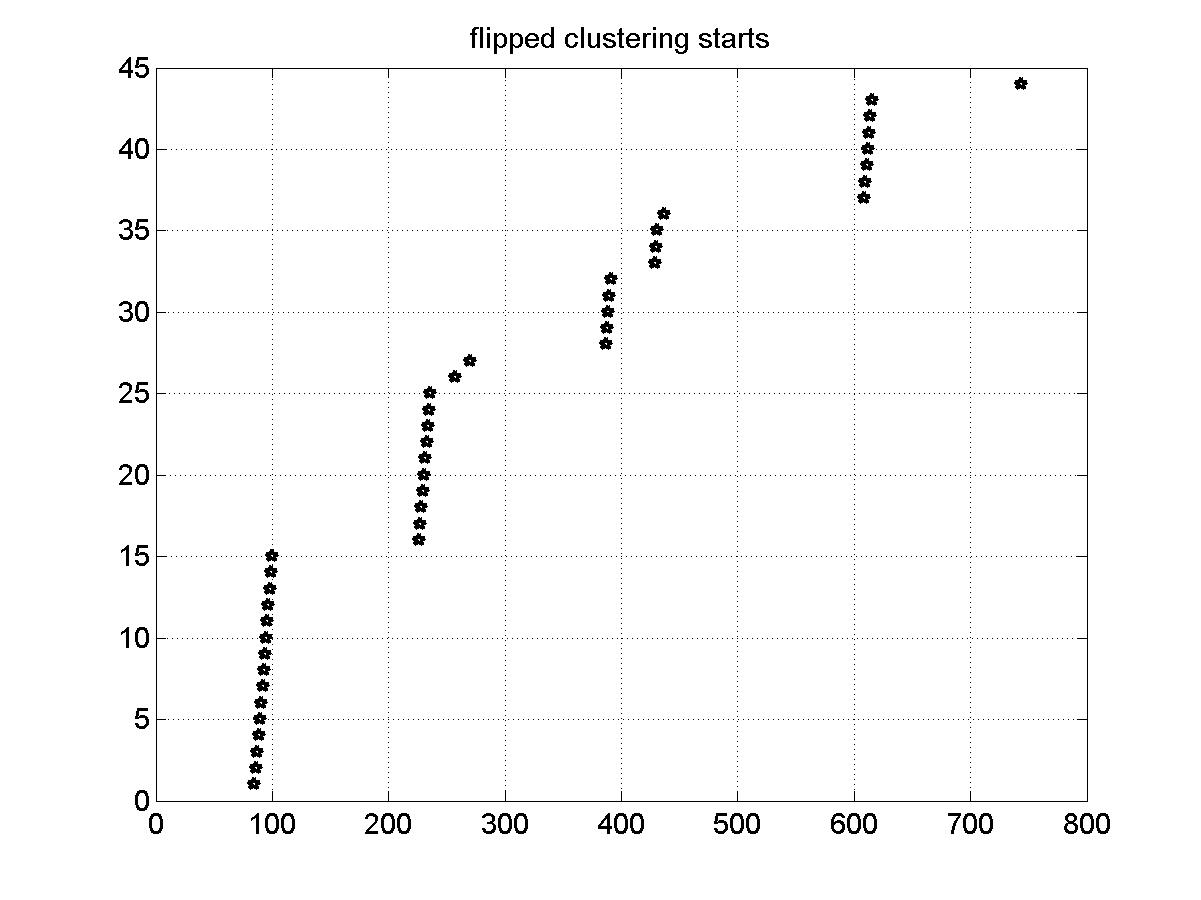


**Figure S3: Similar word distribution and spatial clustering for bach regulatory region, chicken.**
