# Supplementary material for: Some statistical properties of regulatory DNA sequences, and their use in predicting regulatory regions in the Drosophila genome: the fluffy-tail test
Source: BMC Bioinformatics. 2005 Apr 27;6:109. doi: 10.1186/1471-2105-6-109 (PMC1127108; doi:10.1186/1471-2105-6-109)
Supplement: Additional File 3 — Contains a visual example of F dependence on the number of randomisations r. [file 1471-2105-6-109-S3.doc]

# Supplementary Materials to the manuscript 'Some statistical properties of regulatory DNA sequences, and their use in predicting regulatory regions in the Drosophila genome: the fluffy-tail test.' *Irina Abnizova, Klaudia Walter, Rene te Boekhorst and Walter R. Gilks*

Supplementary: a visual example of F dependence on the number of randomisations r.

Obviously, the value F depends on the number of randomisations; the computational time, too.


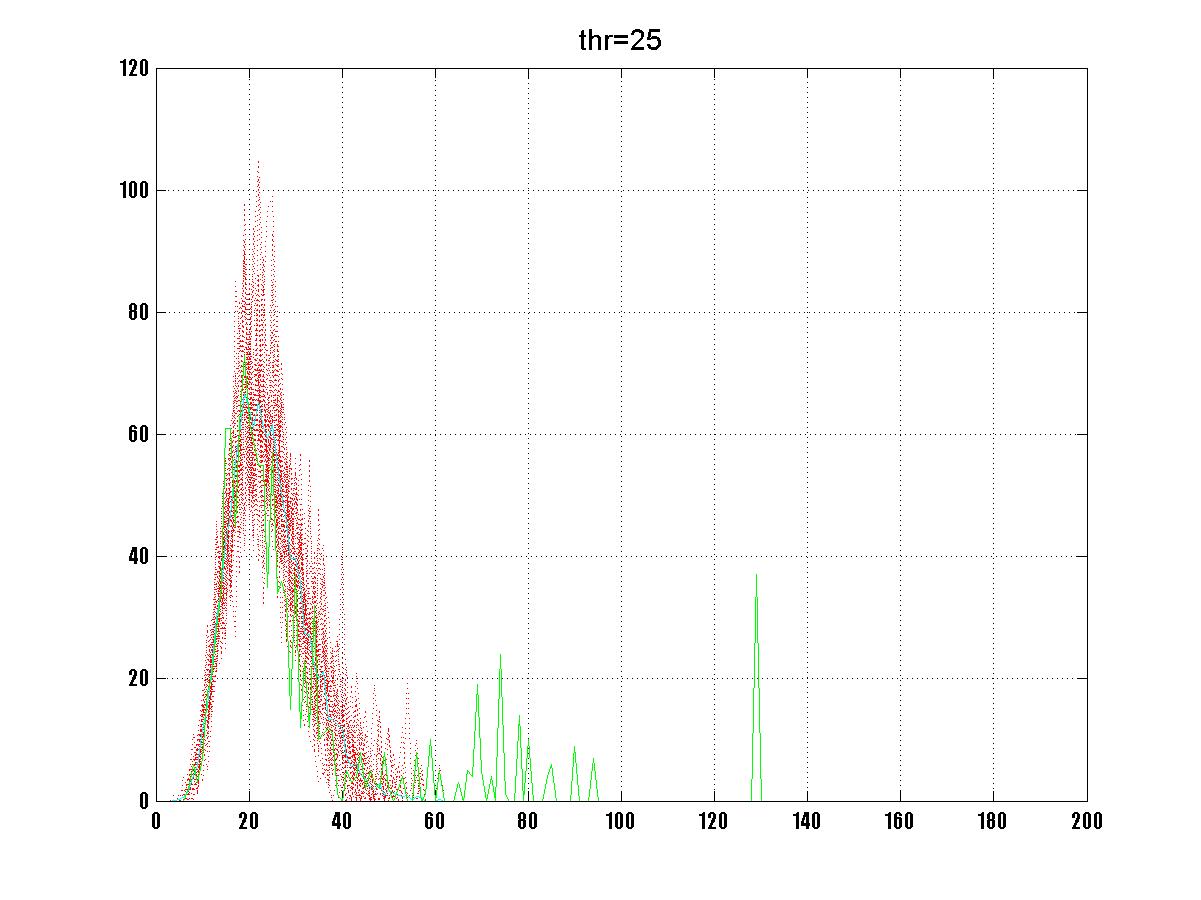


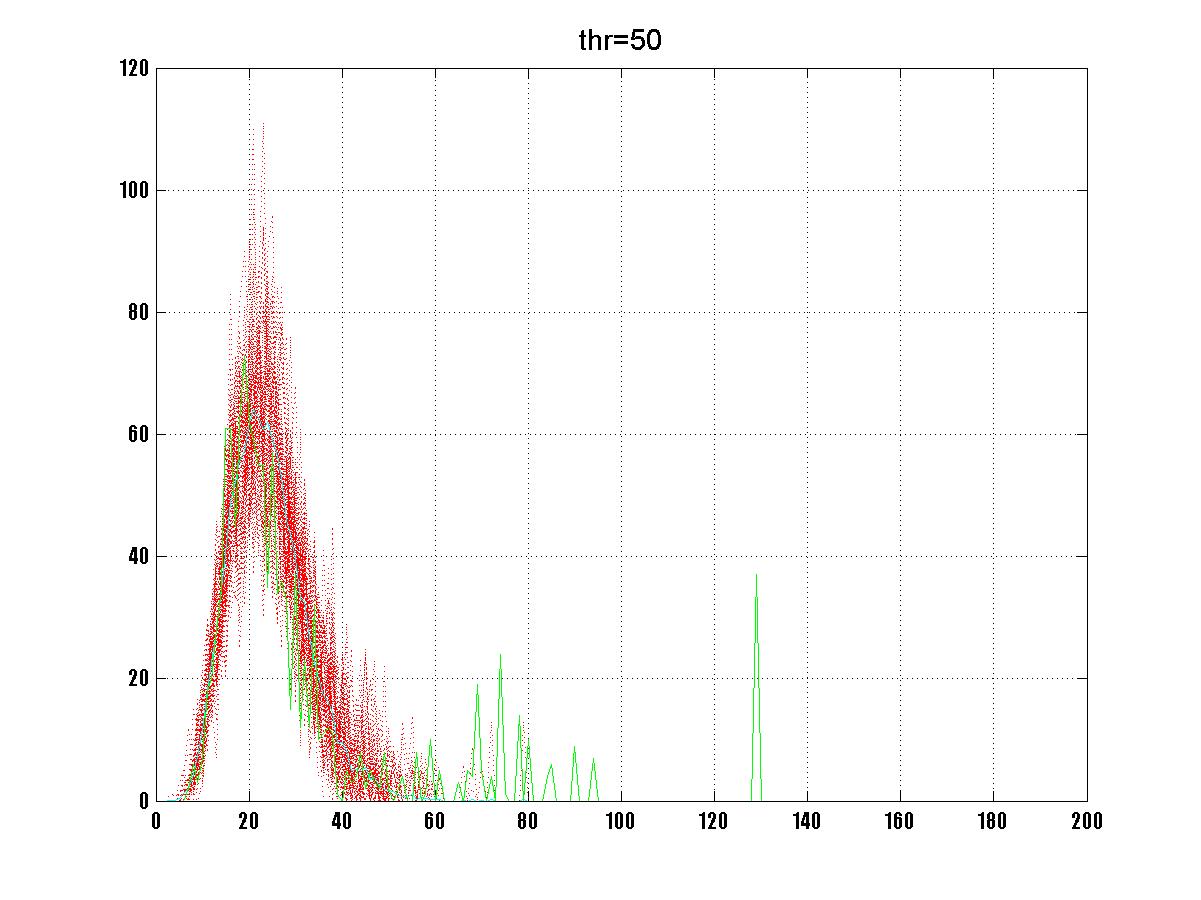


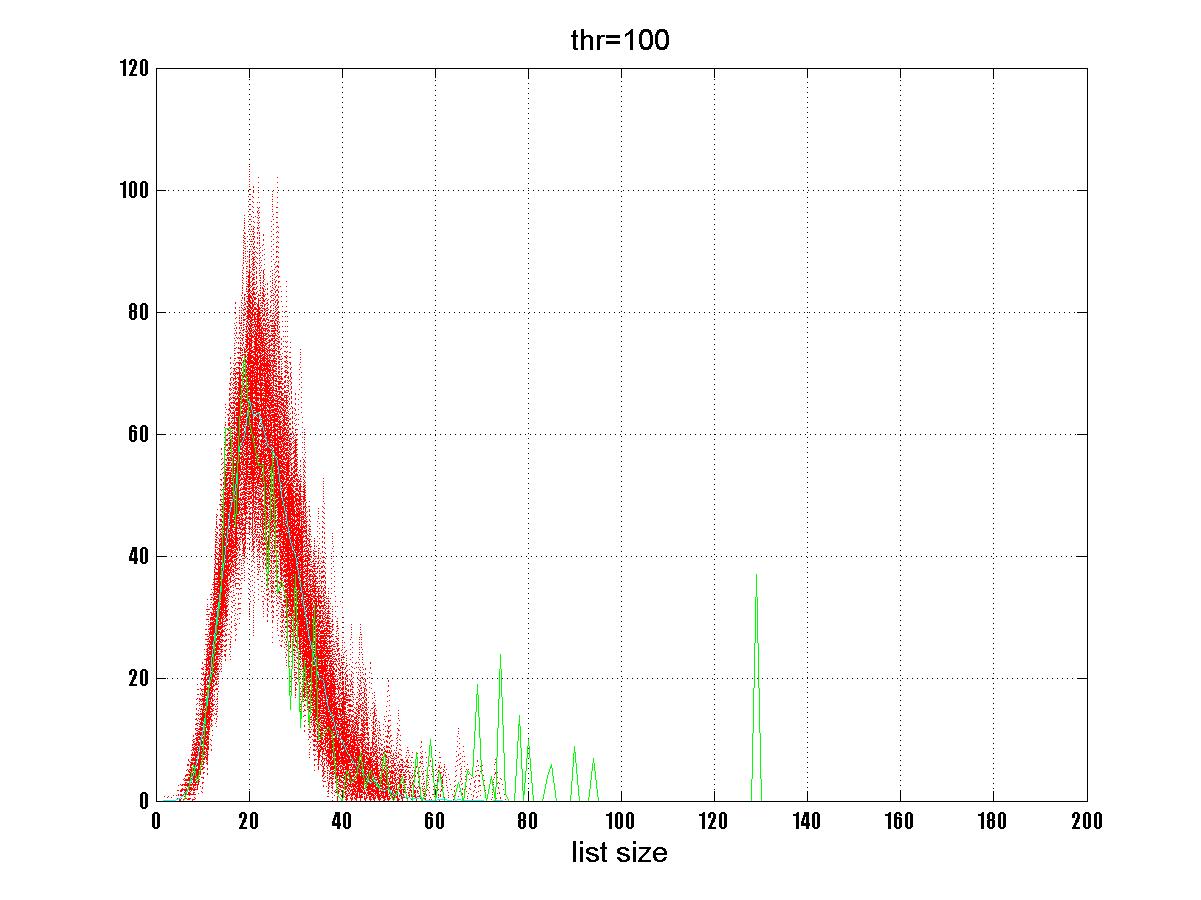


**Figure s4.** **Distributions (histograms) of similar words for kni cis regulatory regions.** X axis is the size of list of similar words, Y axis if the number of lists with this size. Red dotted lines are for randomised sequences, green solid are for original sequence. Upper plot is for NR=25, middle is for NR=50, bottom is for NR=100.

We took number of randomisations 50; one can see at the Figure S4 that no one randomised tail is longer than 80 (red dotted lines) for both 100 and 50 randomisations, assuring that F=8.65 is fair measure of fluffiness here. We did not take NR=25, because it seems to be not enough for reliable estimation of F: on the upper plot Figure 2 all red tails are shorter than 60, while with NR growth (50, 100) there appear some red tails more than 60 less than 80.
