# Supplementary material for: Some statistical properties of regulatory DNA sequences, and their use in predicting regulatory regions in the Drosophila genome: the fluffy-tail test
Source: BMC Bioinformatics. 2005 Apr 27;6:109. doi: 10.1186/1471-2105-6-109 (PMC1127108; doi:10.1186/1471-2105-6-109)
Supplement: Additional File 4 — Gives some more details about spatial clustering threshold [file 1471-2105-6-109-S4.doc]

# Supplementary Materials to the manuscript 'Some statistical properties of regulatory DNA sequences, and their use in predicting regulatory regions in the Drosophila genome: the fluffy-tail test.' *Irina Abnizova, Klaudia Walter, Rene te Boekhorst and Walter R. Gilks*

**Spatial clustering measure: coefficient of variation**

The **coefficient of variation**  CV

CV= sd/mean

is a well known statistical measure of value’s dispersion, it shows whether the values in a set are comparable or there are few exceptional ones.

We tested the **Coefficients of Variation** for the cluster sizes of similar words.

We used an analysis of variance to test for the difference in coefficients of variance for four types of functional DNA: exons, non-fluffy NCNR, fluffy NCNR and regulatory regions.

The assumptions for ANOVA (homogeneity of variance, no correlation between means and standard deviations of the samples) were satisfied, and the outcome is a strongly significant difference between the 4 types.

See Supplementary Table3 below:

**Supplementary Table3**

We used CV to distinguish fluffy NCNR from regulatory DNA. CV for fluffy NCNR almost always more than 1, they significantly differ from CV for regulatory DNA.

You can also see this in the Figure9 in the paper. A Newman-Keuls range test was performed to find out between what types these significant differences occur. Apart from the difference between non-tailed junk and regulatory regions (not significant, p > 0.08), all other differences are significant.
