# Supplementary material for: Some statistical properties of regulatory DNA sequences, and their use in predicting regulatory regions in the Drosophila genome: the fluffy-tail test
Source: BMC Bioinformatics. 2005 Apr 27;6:109. doi: 10.1186/1471-2105-6-109 (PMC1127108; doi:10.1186/1471-2105-6-109)
Supplement: Additional File 5 — Shows some examples for consistence of fluffiness for different word length, tables. [file 1471-2105-6-109-S5.doc]

# Supplementary Materials to the manuscript 'Some statistical properties of regulatory DNA sequences, and their use in predicting regulatory regions in the Drosophila genome: the fluffy-tail test.' *Irina Abnizova, Klaudia Walter, Rene te Boekhorst and Walter R. Gilks*

Consistence of fluffiness for different word length.

Supplemental Table2: The number of fluffy sequences for regulatory, exons, non-coding non-regulatory (NCNR) training DNA sets depending on word length.

| m, mim | Fluffy regulatory  F>2 | Non fluffy  Regulatory  F<2 | Fluffy exons  F>2 | Non fluffy  exons  F<2 | Fluffy NCNR  F>2 | Non fluffy  NCNR  F<2 |
| --- | --- | --- | --- | --- | --- | --- |
| 3,0 | 52 | 8 | 2 | 58 | 5 | 55 |
| 5,1 | 51 | 9 | 1 | 59 | 10 | 60 |
| 7,2 | 52 | 8 | 1 | 59 | 8 | 52 |
| 9,3 | 53 | 7 | 3 | 57 | 3 | 57 |
| 12,4 | 54 | 6 | 6 | 54 | 7 | 53 |

Note, that we are able to distinguish regulatory ( most part of sequences from positive training set passed the F-test: occurred fluffy) from coding and non-coding non-regulatory for different word length and mismatches. NCNR DNA was repeat-masked. In the paper we showed the results for (5,1) , see Table 2.
