# Supplementary material for: Some statistical properties of regulatory DNA sequences, and their use in predicting regulatory regions in the Drosophila genome: the fluffy-tail test
Source: BMC Bioinformatics. 2005 Apr 27;6:109. doi: 10.1186/1471-2105-6-109 (PMC1127108; doi:10.1186/1471-2105-6-109)
Supplement: Additional File 6 — Shows some examples for consistence of fluffiness for different word length in the histogram form [file 1471-2105-6-109-S6.doc]

# Supplementary Materials to the manuscript 'Some statistical properties of regulatory DNA sequences, and their use in predicting regulatory regions in the Drosophila genome: the fluffy-tail test.' *Irina Abnizova, Klaudia Walter, Rene te Boekhorst and Walter R. Gilks*

Consistence of Fluffiness for different word length: visualization.

On the Figures S5-S9 below one can see the separation of regulatory DNA (green) from exons (cyan) and non-coding and non regulatory (magenta) for different word length m=3,5,7,9,12 and corresponding mismatches mim=0,1,2,3,4


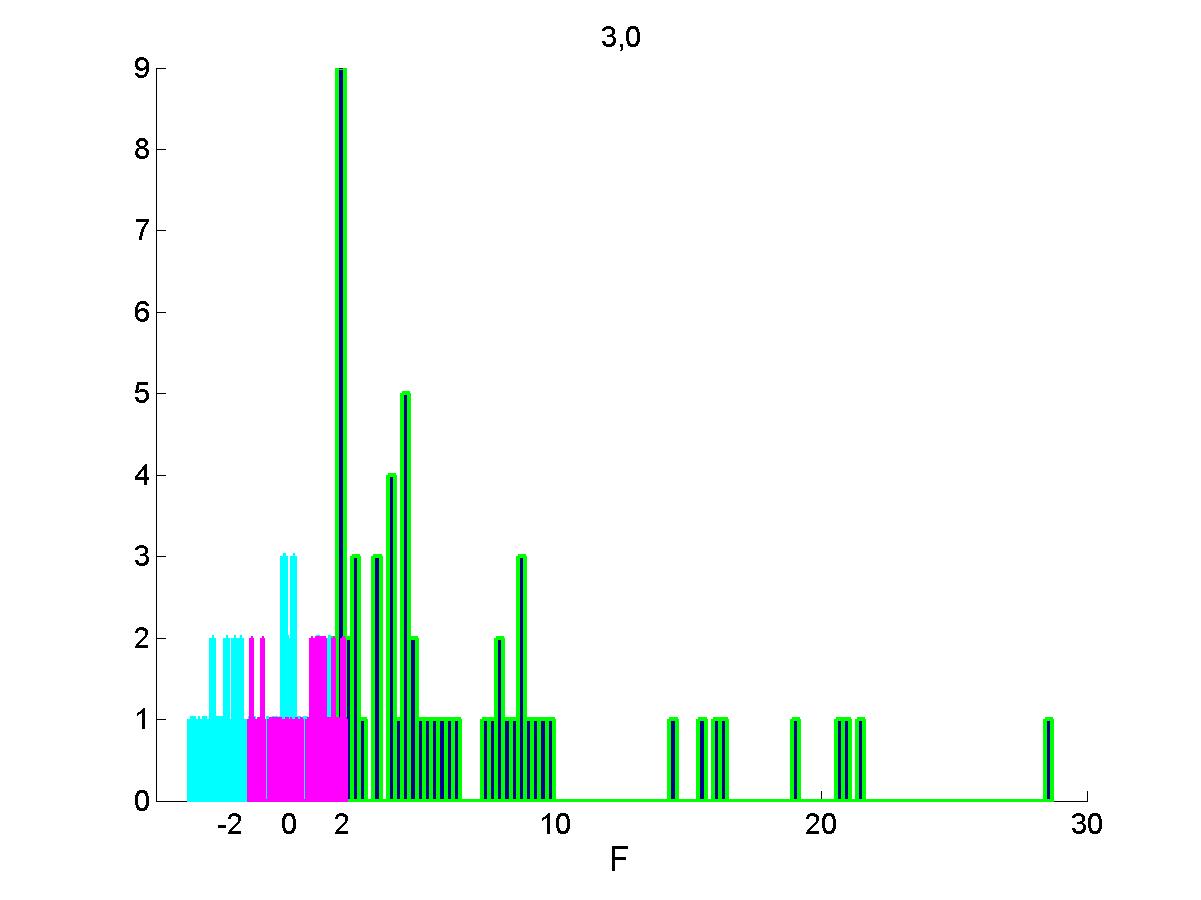


**Figure S5**: Separation of regulatory DNA (green) from other types of DNA for (m.mim)=(3,0). X axis shows the value F, y axis shows the number of sequences with this F for exons (cyan), NCNR(magenta) and regulatory (green) DNA.


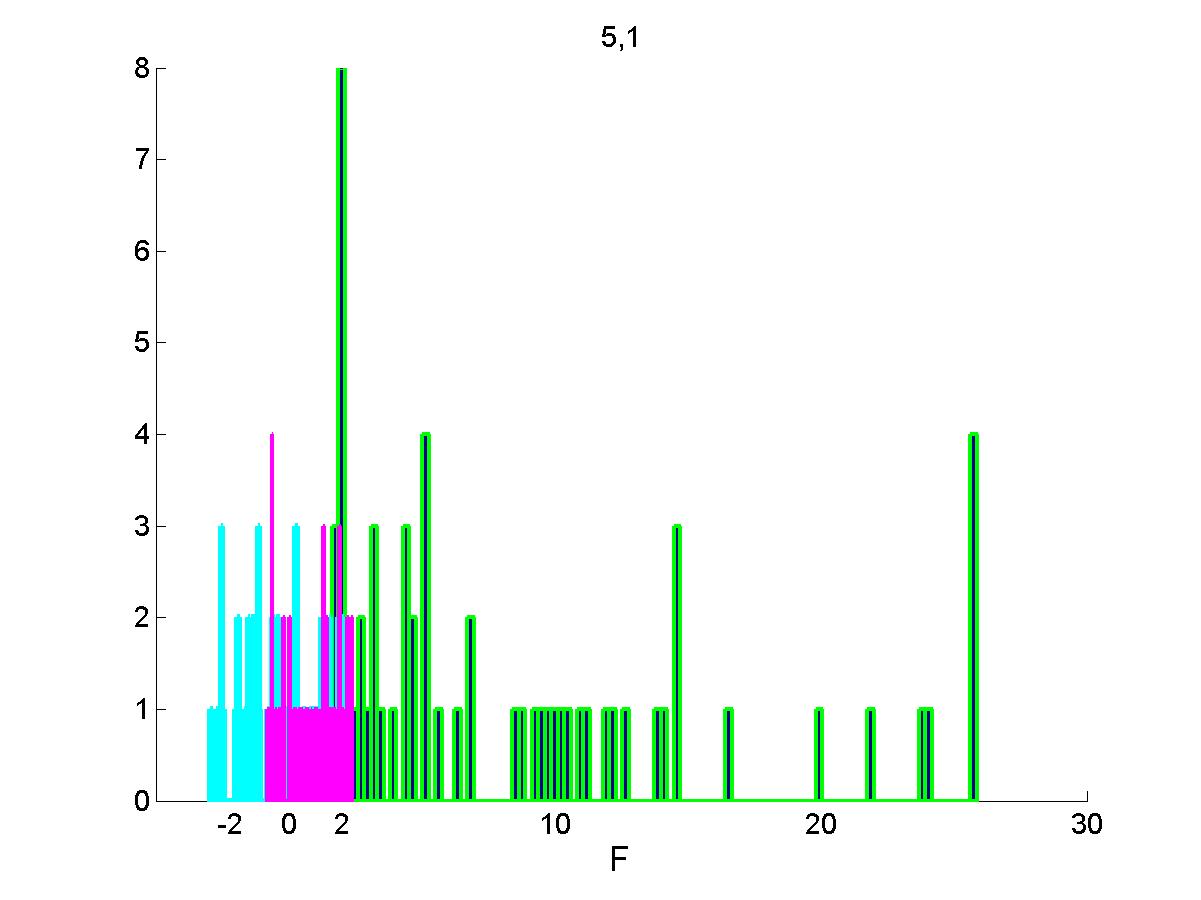


**Figure S6**: Separation of regulatory DNA (green) from other types of DNA for (m.mim)=(5,1). X axis shows the value F, Y axis shows the number of sequences with this F for exons (cyan), NCNR(magenta) and regulatory (green) DNA.


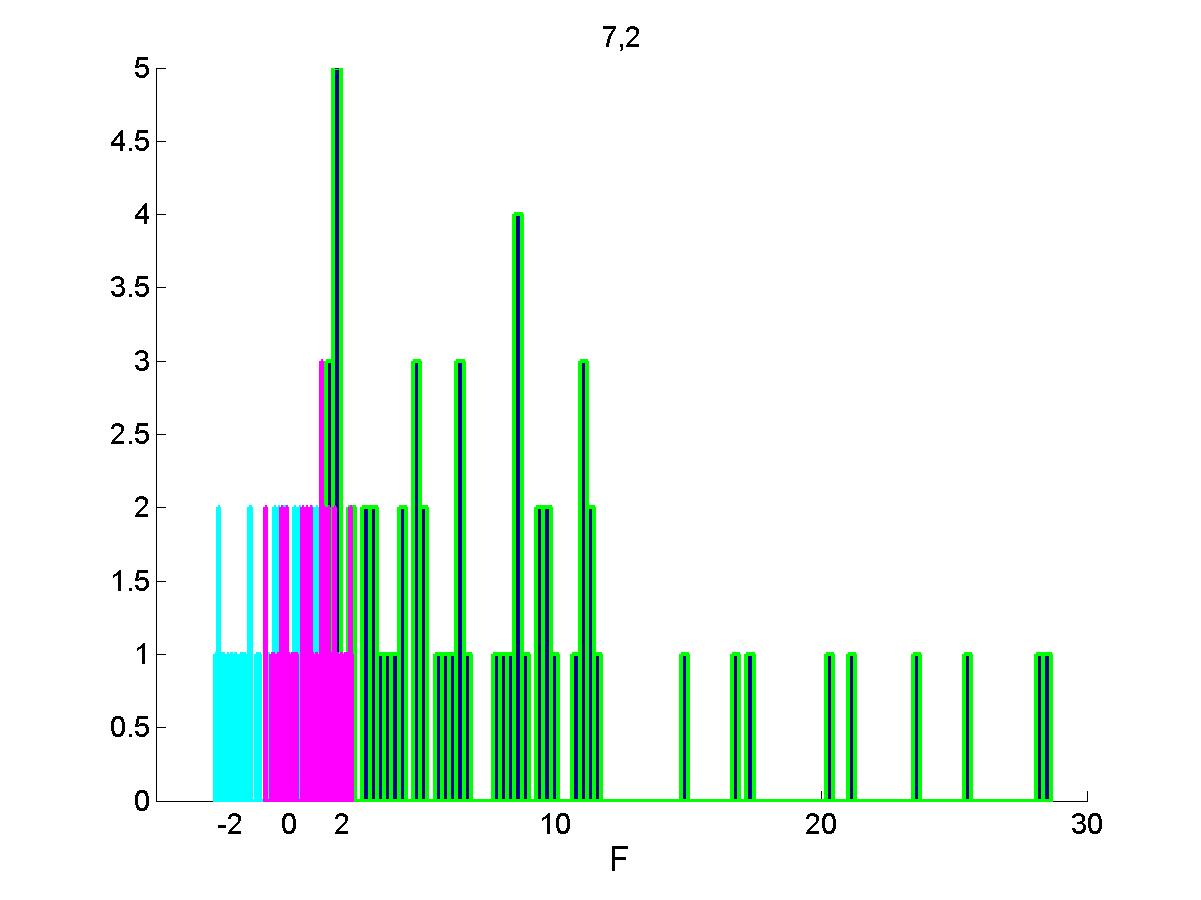


**Figure S7**: Separation of regulatory DNA (green) from other types of DNA for (m.mim)=(7,2). X axis shows the value F, Y axis shows the number of sequences with this F for exons (cyan), NCNR(magenta) and regulatory (green) DNA.


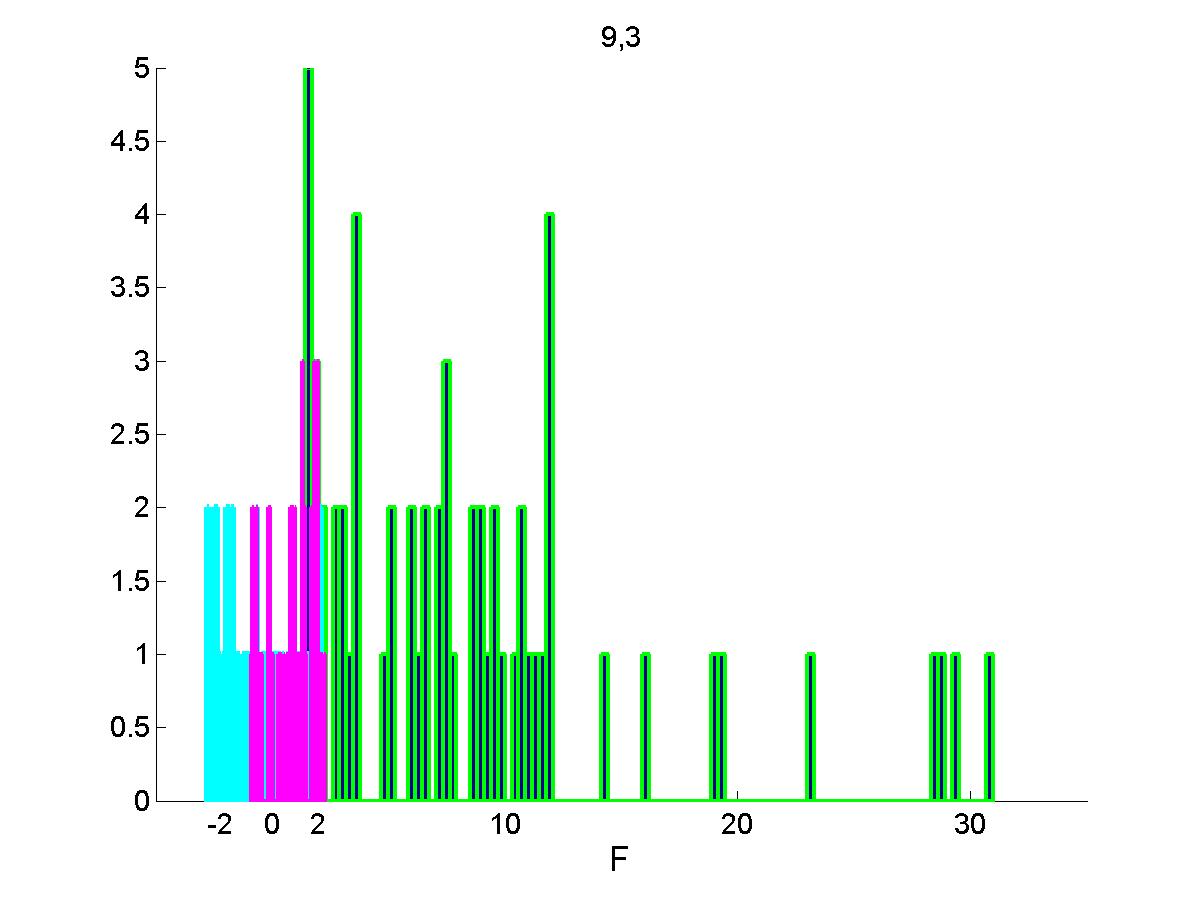


**Figure S8**: Separation of regulatory DNA (green) from other types of DNA for (m.mim)=(9,3). X axis shows the value F, Y axis shows the number of sequences with this F for exons (cyan), NCNR(magenta) and regulatory (green) DNA.


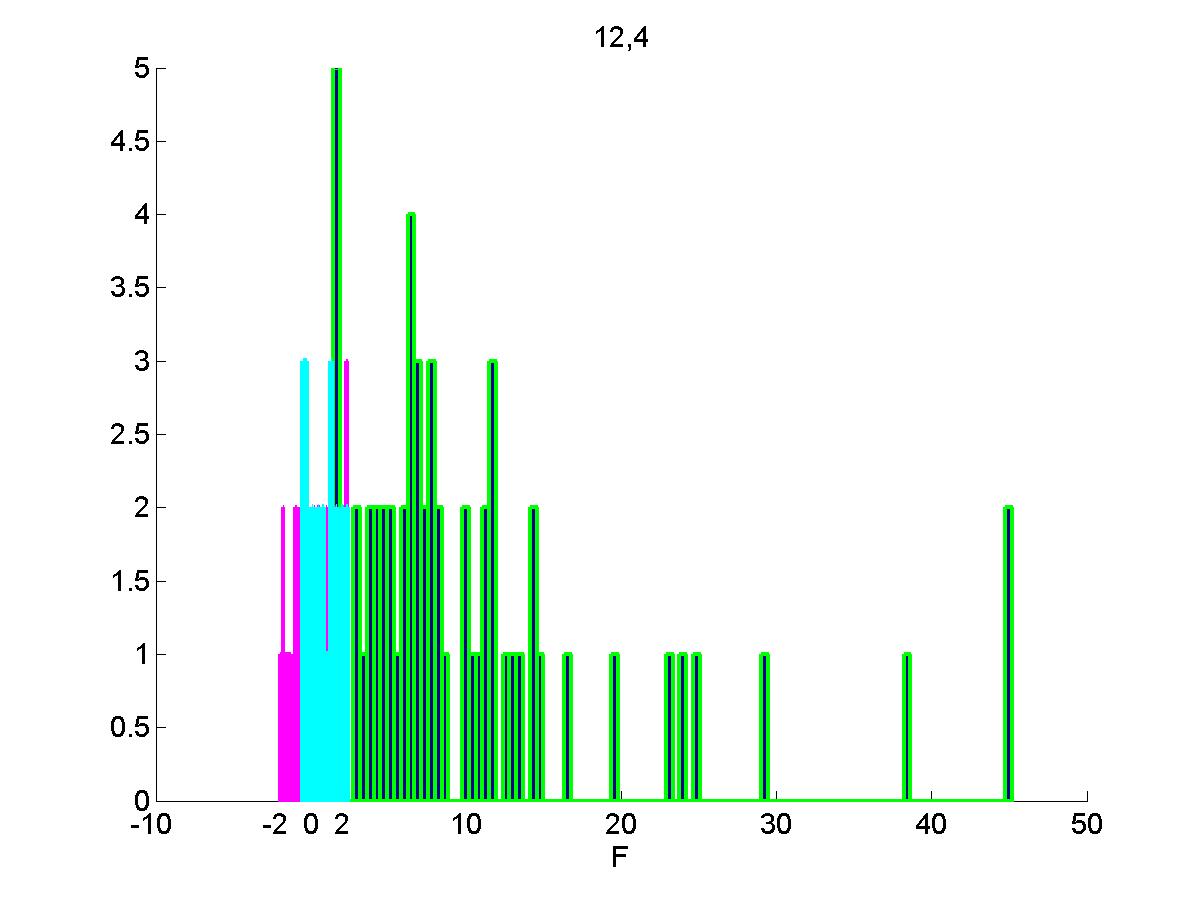


**Figure S9**: Separation of regulatory DNA (green) from other types of DNA for (m.mim)=(12,4). X axis shows the value F, Y axis shows the number of sequences with this F for exons (cyan), NCNR(magenta) and regulatory (green) DNA.
