# Supplementary material for: Some statistical properties of regulatory DNA sequences, and their use in predicting regulatory regions in the Drosophila genome: the fluffy-tail test
Source: BMC Bioinformatics. 2005 Apr 27;6:109. doi: 10.1186/1471-2105-6-109 (PMC1127108; doi:10.1186/1471-2105-6-109)
Supplement: Additional File 7 — Consistent fluffiness and coefficient of variation for spatial cluster size for some example sequences. [file 1471-2105-6-109-S7.doc]

# Supplementary Materials to the manuscript 'Some statistical properties of regulatory DNA sequences, and their use in predicting regulatory regions in the Drosophila genome: the fluffy-tail test.' *Irina Abnizova, Klaudia Walter, Rene te Boekhorst and Walter R. Gilks*

Consistent fluffiness and coefficient of variation for spatial cluster size for some example sequences:

Table s1: F and CV for exon 2r4 for different values (m,mim).

| m,mim | F | CV |
| --- | --- | --- |
| 3,0 | 1.6 | 0.52 |
| 5,1 | 0.85 | 0.69 |
| 7,2 | 2.0 | 0.62 |
| 8,2 | 1.6 | 0.64 |
| 9,3 | 1.91 | 0.55 |
| 12,4 | 1.18 | 0.33 |

Table s2: F and CV for abdominantA regulatory region for different values (m,mim).

| m,mim | F | CV |
| --- | --- | --- |
| 3,0 | 14.10 | 0.59 |
| 5,1 | 11.26 | 0.77 |
| 7,2 | 18.3 | 0.70 |
| 9,3 | 13.9 | 1.07 |
| 12,4 | 8.7 | 0.82 |

Table s3: F and CV for knirps regulatory region for different values (m,mim).

| m,mim | F | CV |
| --- | --- | --- |
| 3,0 | 11.18 | 0.78 |
| 5,1 | 8.65 | 0.68 |
| 7,2 | 10.11 | 0.78 |
| 9,3 | 16.4 | 0.56 |
| 12,4 | 23.5 | 0.90 |

Table s4: F and CV for NCNR region 3L4 for different values (m,mim).

| m,mim | F | CV |
| --- | --- | --- |
| 3,0 | 12.8 | 1.07 |
| 5,1 | 12.6 | 1.02 |
| 7,2 | 6.17 | 0.95 |
| 9,3 | 19.27 | 0.89 |
| 12,4 | 50.34 | 1.3 |

Table s5: F and CV for NCNR masked region 3L4 for different values (m,mim).

| m,mim | F | CV |
| --- | --- | --- |
| 3,0 | 0.68 | 0.62 |
| 5,1 | 2.0 | 0.75 |
| 7,2 | 0.84 | 0.71 |
| 9,3 | 1.36 | 0.79 |
| 12,4 | 0.17 | 0.48 |
