# Supplementary material for: Some statistical properties of regulatory DNA sequences, and their use in predicting regulatory regions in the Drosophila genome: the fluffy-tail test
Source: BMC Bioinformatics. 2005 Apr 27;6:109. doi: 10.1186/1471-2105-6-109 (PMC1127108; doi:10.1186/1471-2105-6-109)
Supplement: Additional File 8 — Contains the Figures showing fluffiness and spatial clustering of similar words for NCNR 3L4 region. [file 1471-2105-6-109-S8.doc]

# Supplementary Materials to the manuscript 'Some statistical properties of regulatory DNA sequences, and their use in predicting regulatory regions in the Drosophila genome: the fluffy-tail test.' *Irina Abnizova, Klaudia Walter, Rene te Boekhorst and Walter R. Gilks*

F and CV for different (m,mim): visualization for NCNR 3L4 region.

Table s4: F and CV for NCNR region 3L4 for different values (m,mim).

| m,mim | F | CV |
| --- | --- | --- |
| 3,0 | 12.8 | 1.07 |
| 5,1 | 12.6 | 1.02 |
| 7,2 | 6.17 | 0.95 |
| 9,3 | 19.27 | 0.89 |
| 12,4 | 50.34 | 1.3 |


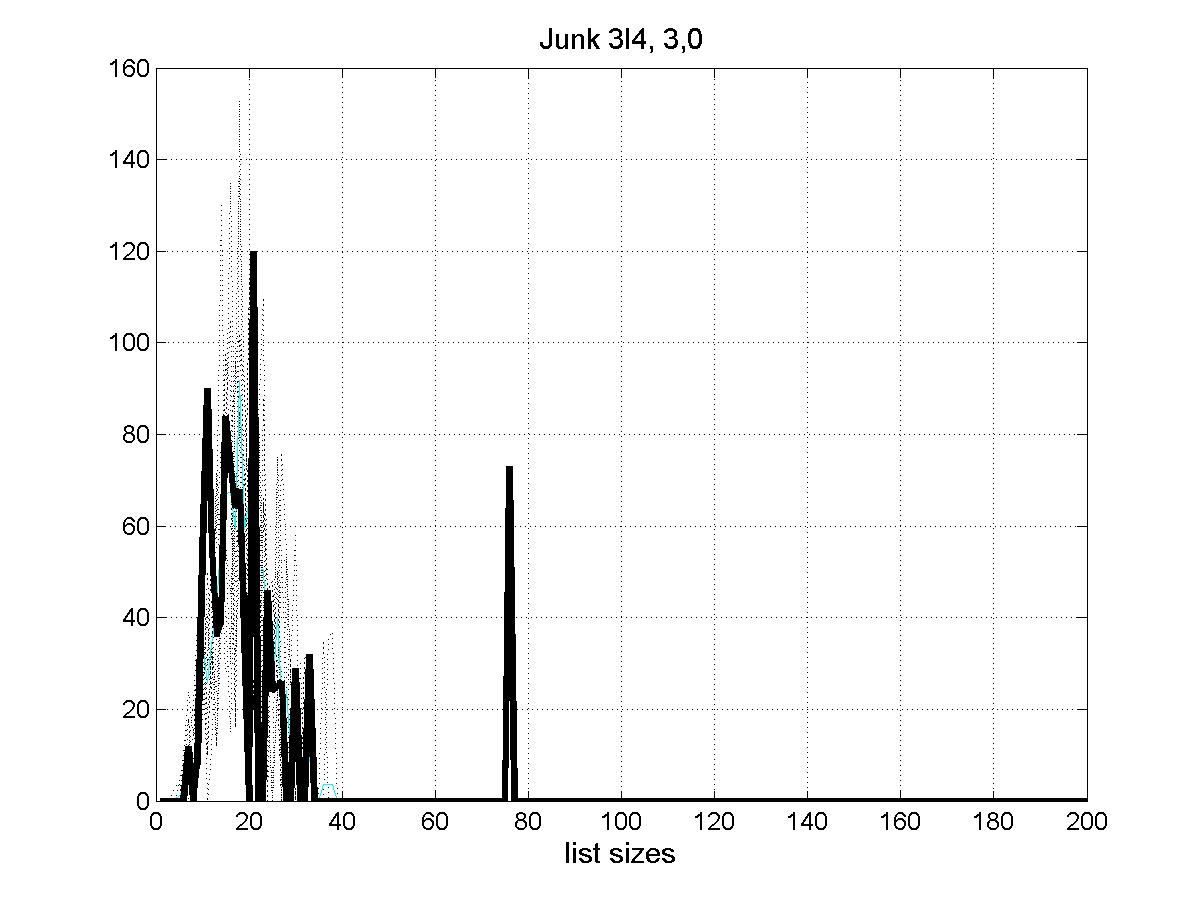

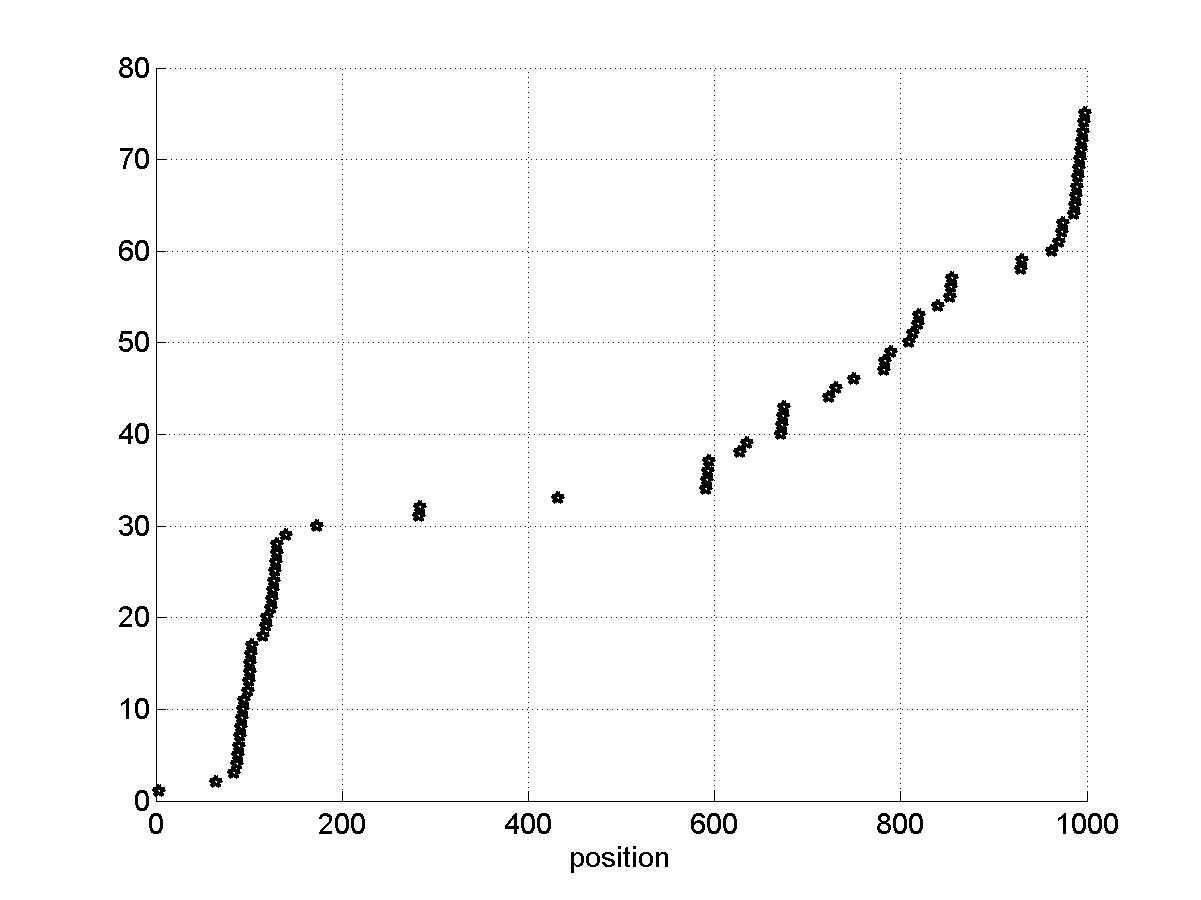


**Figure S10: Similar word distribution and spatial clustering for NCNR region 3L4, (m,mim)= 3,0**


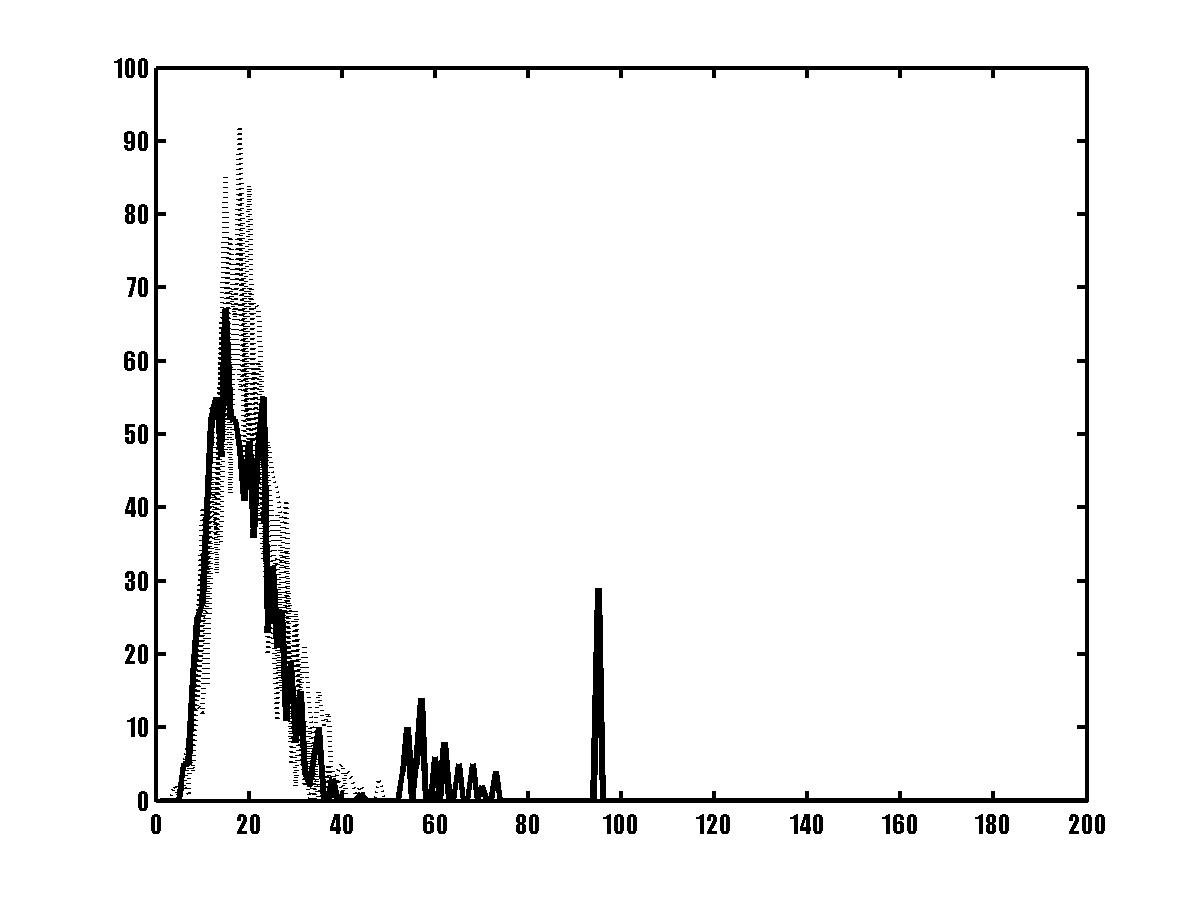

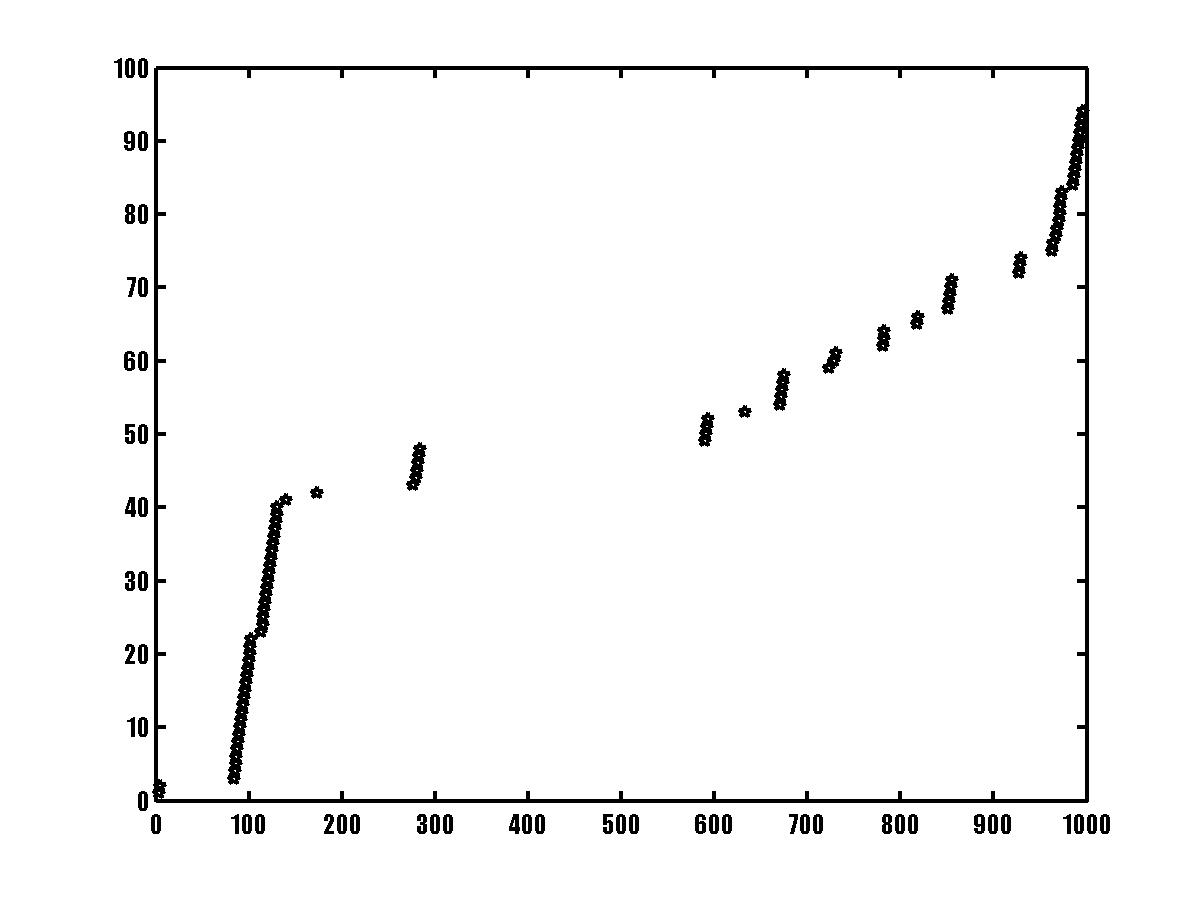


**Figure S11: Similar word distribution and spatial clustering for NCNR region 3L4, (m,mim)=(5,1).**


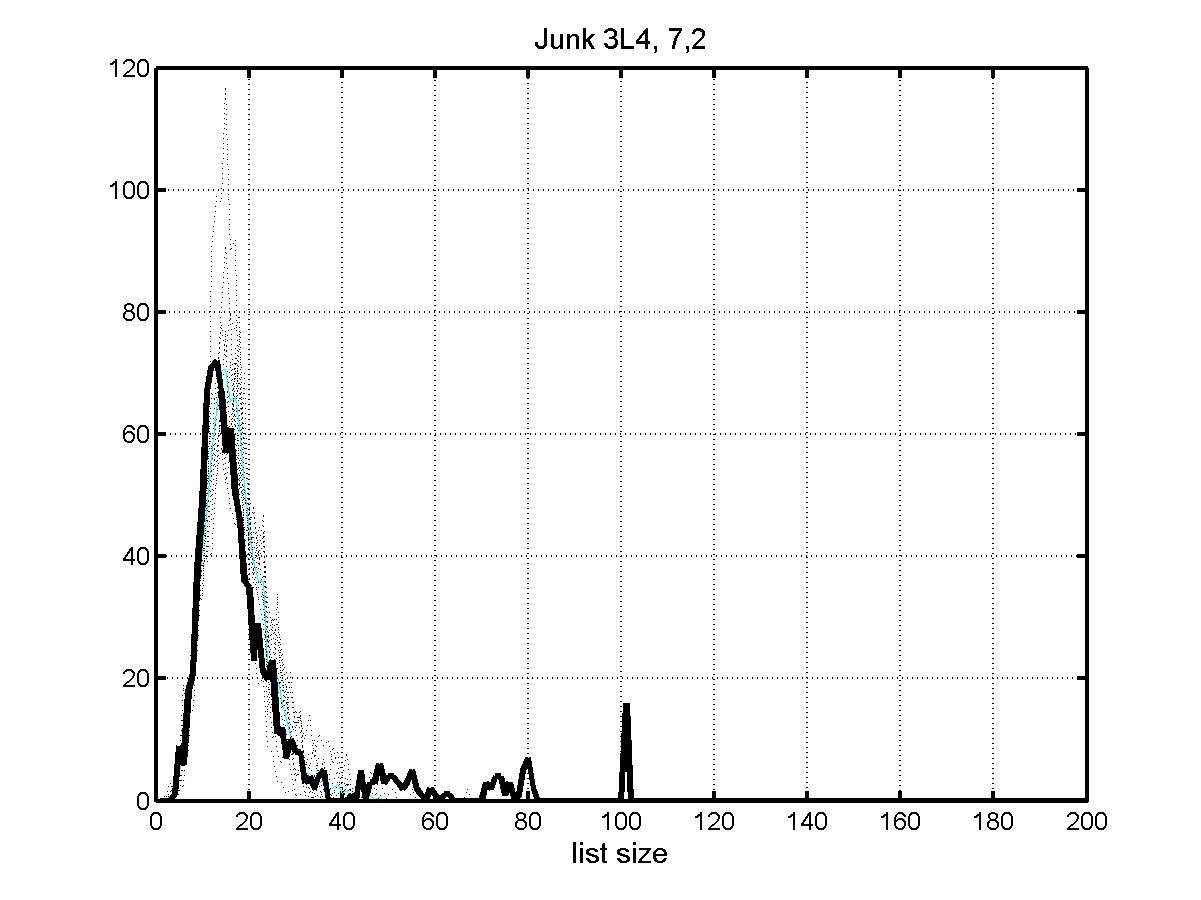

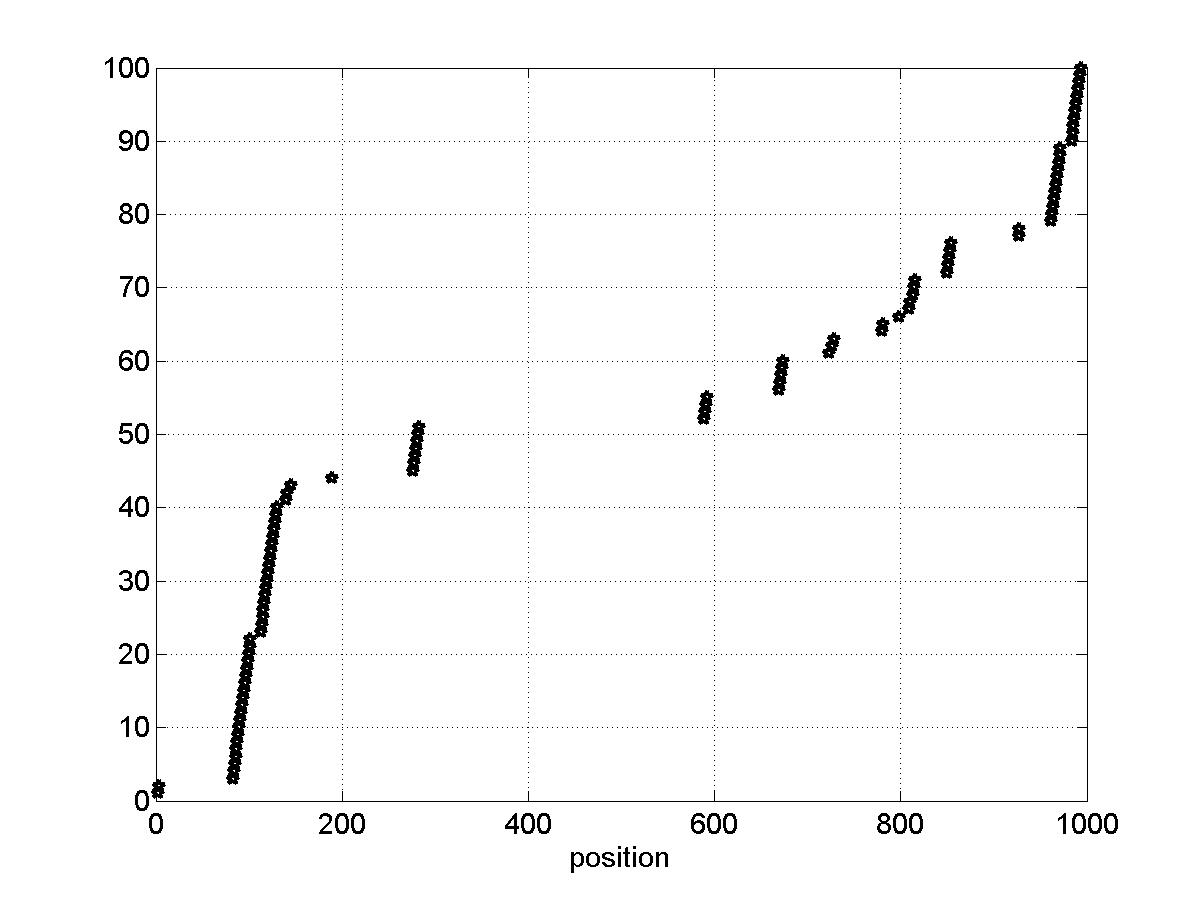


**Figure S12: Similar word distribution and spatial clustering for NCNR region 3L4, (m,mim)=(7,2).**


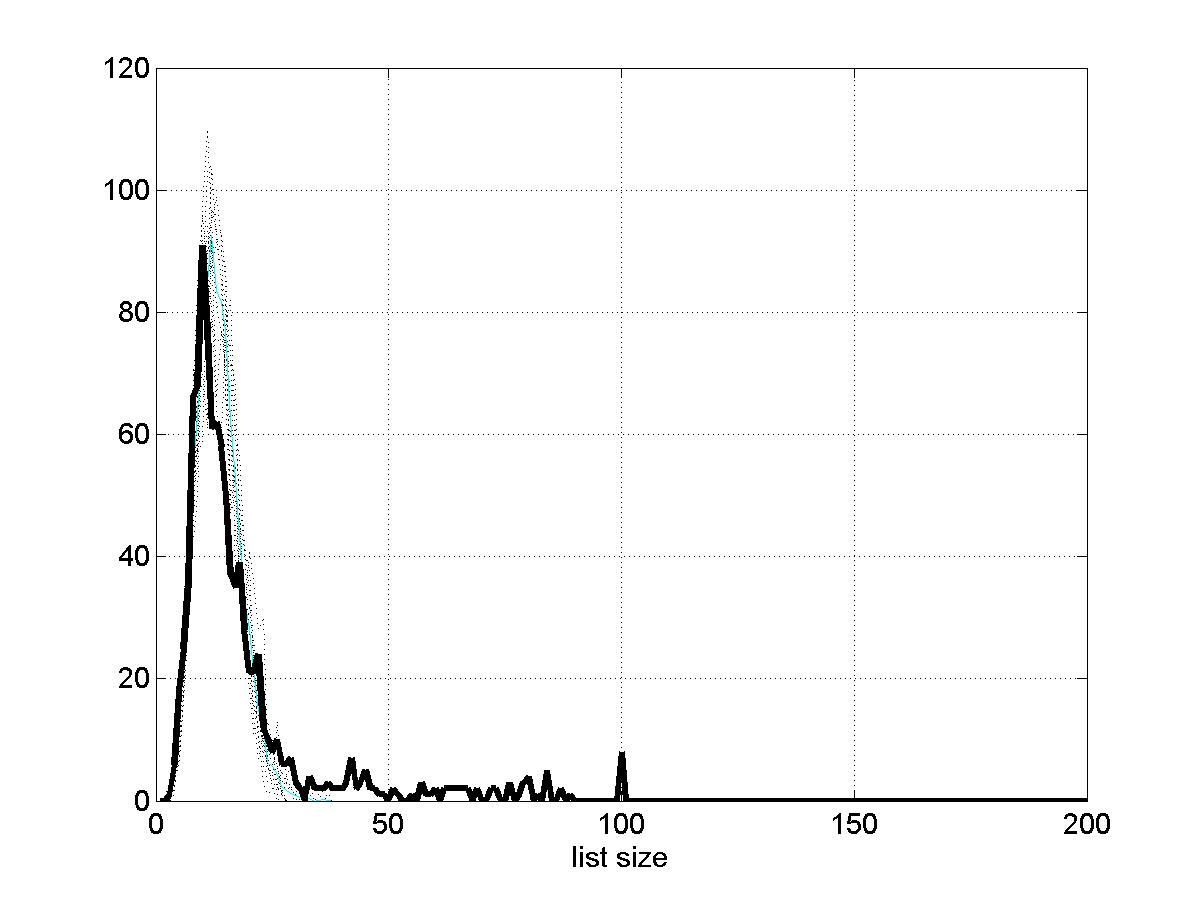

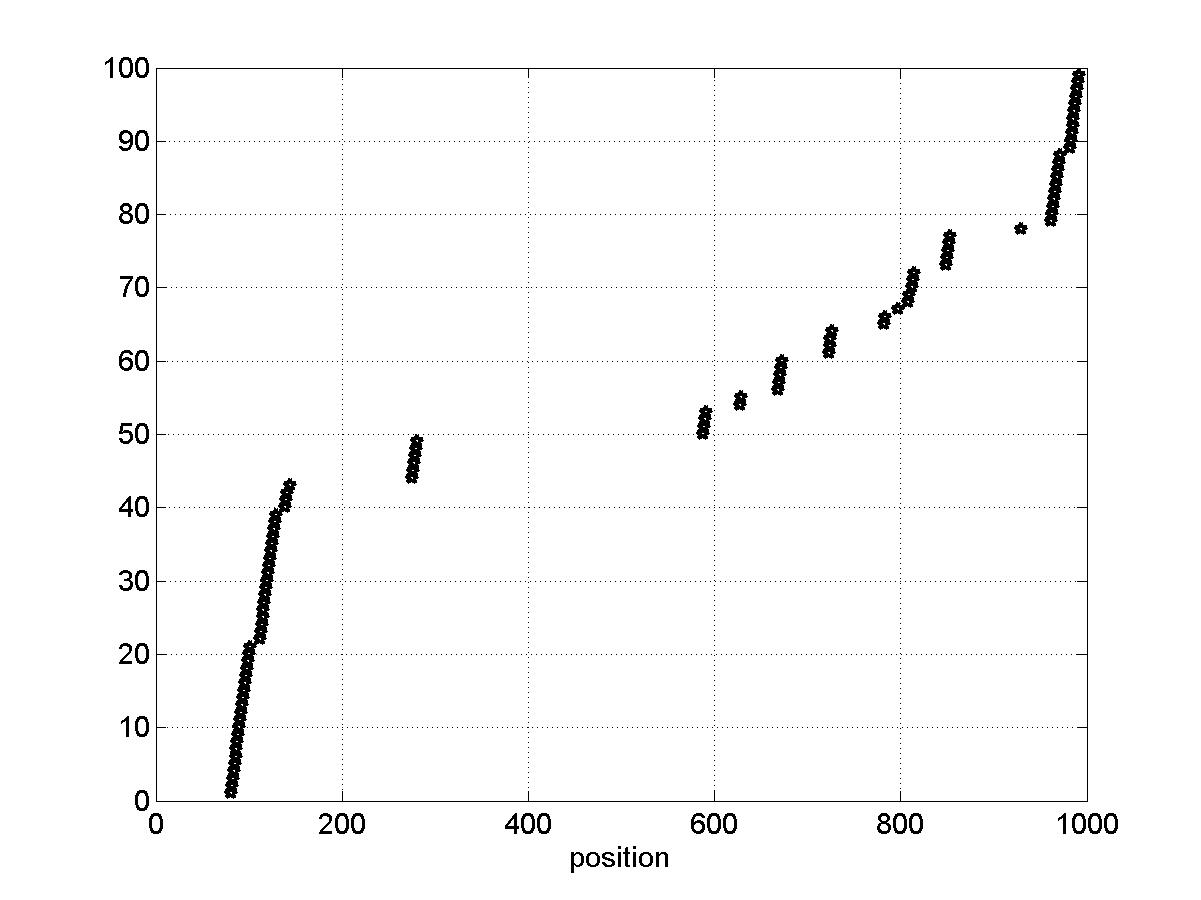


**Figure S13: Similar word distribution and spatial clustering for NCNR region 3L4, (m,mim)=(9,3).**


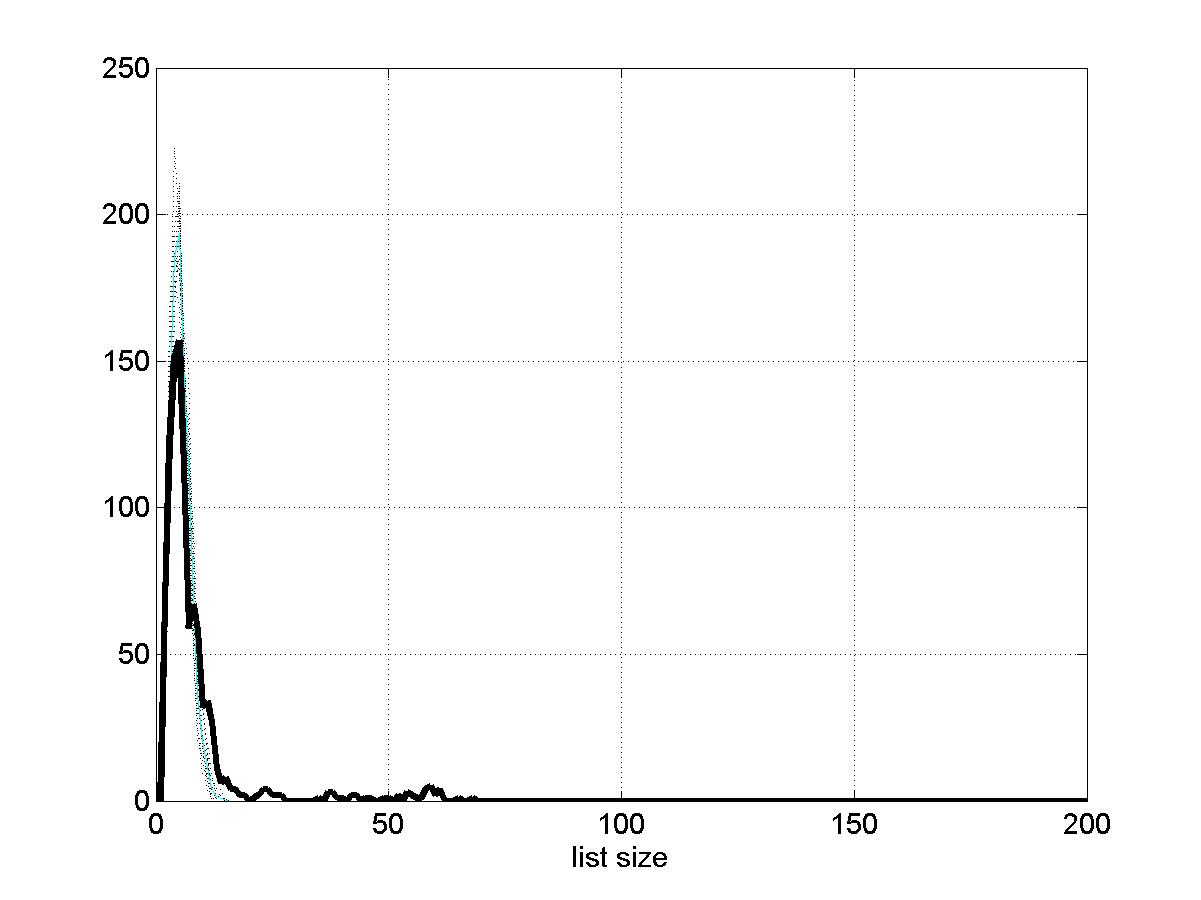

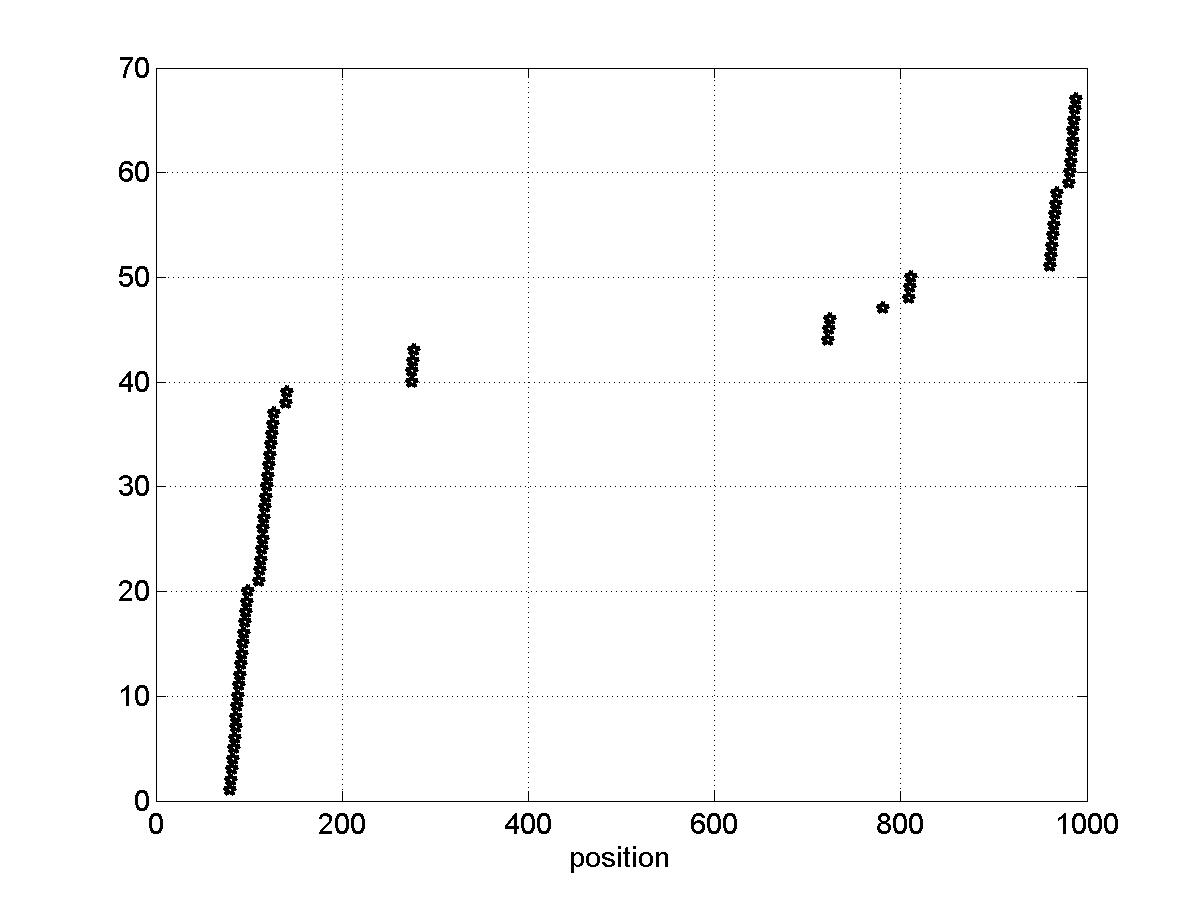


**Figure S14: Similar word distribution and spatial clustering for NCNR region 3L4, (m,mim)=(12,4).**
