# Supplementary material for: Some statistical properties of regulatory DNA sequences, and their use in predicting regulatory regions in the Drosophila genome: the fluffy-tail test
Source: BMC Bioinformatics. 2005 Apr 27;6:109. doi: 10.1186/1471-2105-6-109 (PMC1127108; doi:10.1186/1471-2105-6-109)
Supplement: Additional File 9 — Contains the Figures showing fluffiness and spatial clustering of similar words for NCNR repeat-masked 3L4 region. [file 1471-2105-6-109-S9.doc]

# Supplementary Materials to the manuscript 'Some statistical properties of regulatory DNA sequences, and their use in predicting regulatory regions in the Drosophila genome: the fluffy-tail test.' *Irina Abnizova, Klaudia Walter, Rene te Boekhorst and Walter R. Gilks*

Supplementary 3L4 masked

Table s5: F and CV for NCNR masked region 3L4 for different values (m,mim).

| m,mim | F | CV |
| --- | --- | --- |
| 3,0 | 0.68 | 0.62 |
| 5,1 | 2.0 | 0.75 |
| 7,2 | 0.84 | 0.71 |
| 9,3 | 1.36 | 0.79 |
| 12,4 | 0.17 | 0.48 |


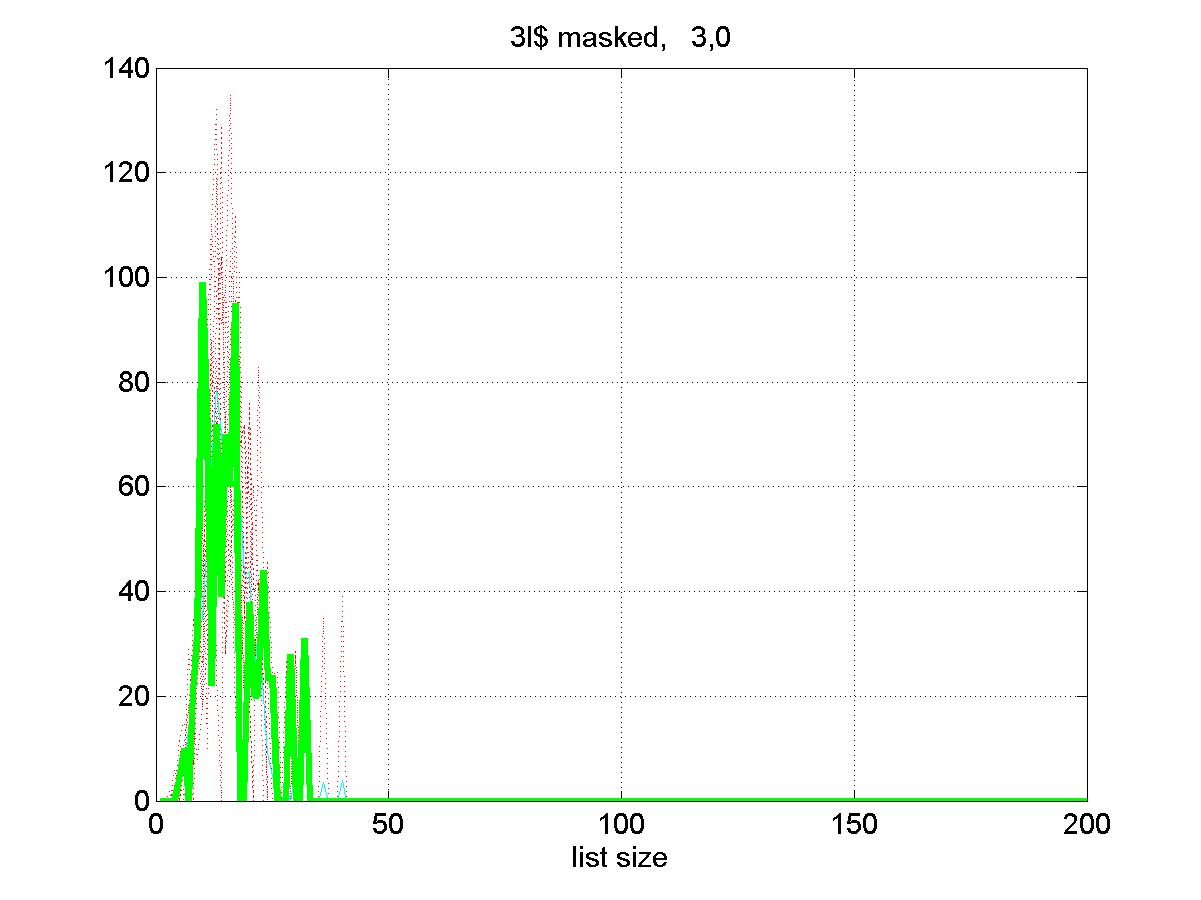

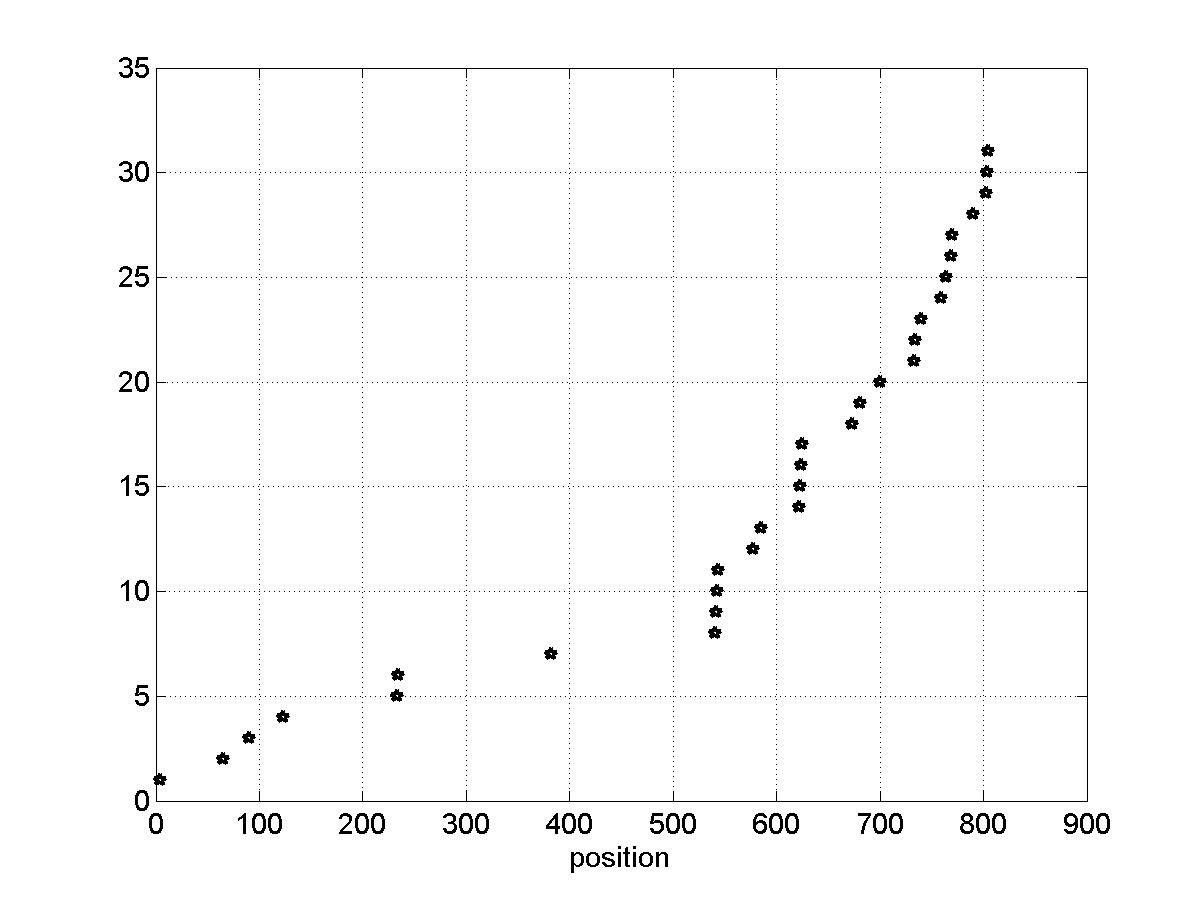


**Figure S15: Similar word distribution and spatial clustering for masked NCNR region 3L4, (m,mim)=(3,0).**


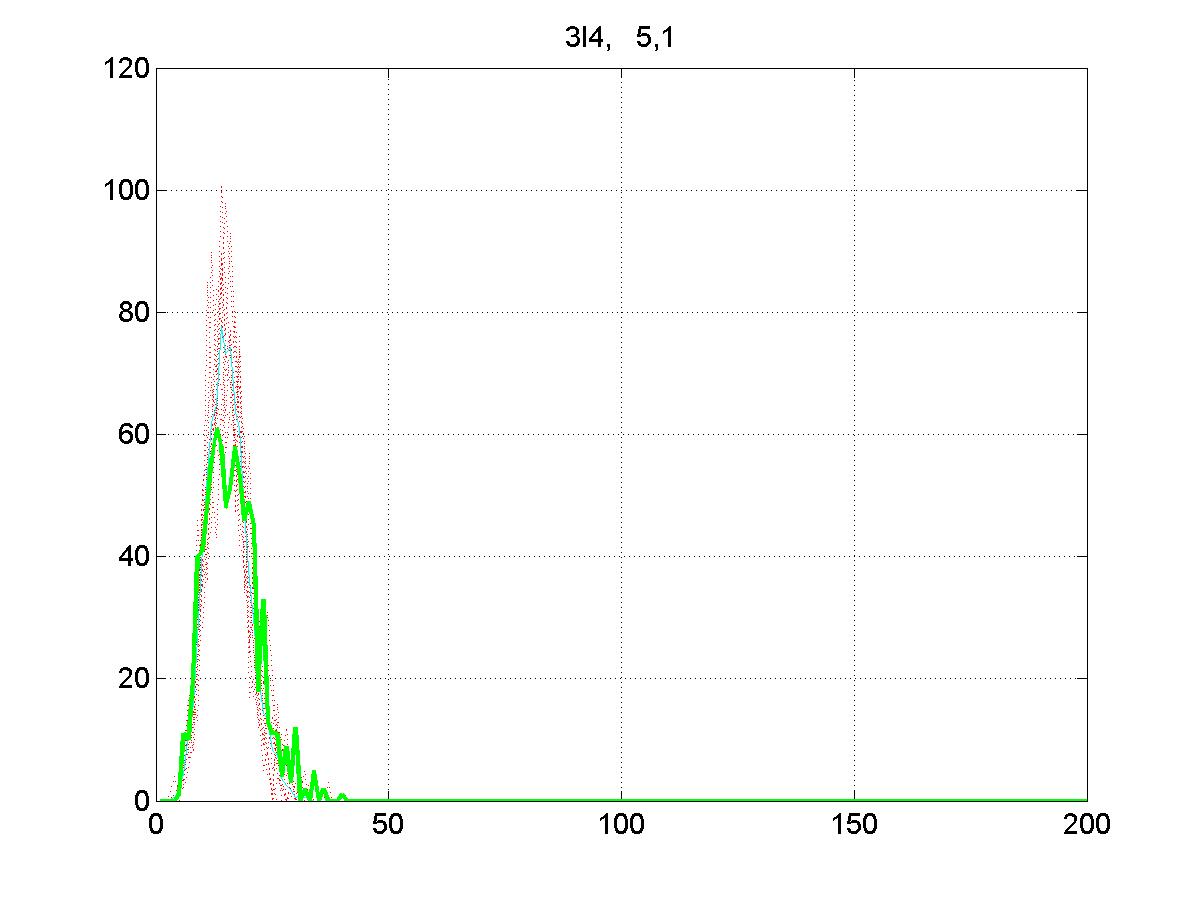

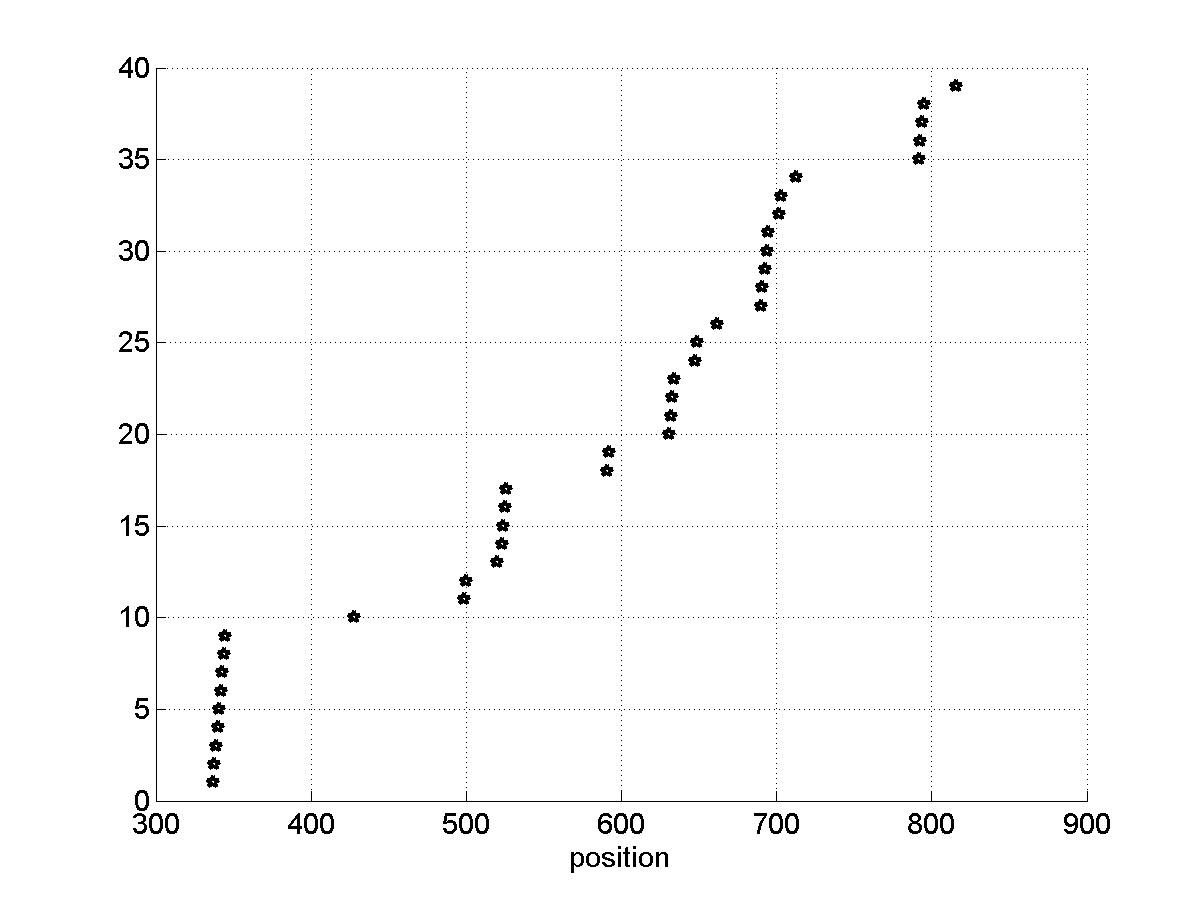


**Figure S16: Similar word distribution and spatial clustering for masked NCNR region 3L4, (m,mim)=(5,1).**


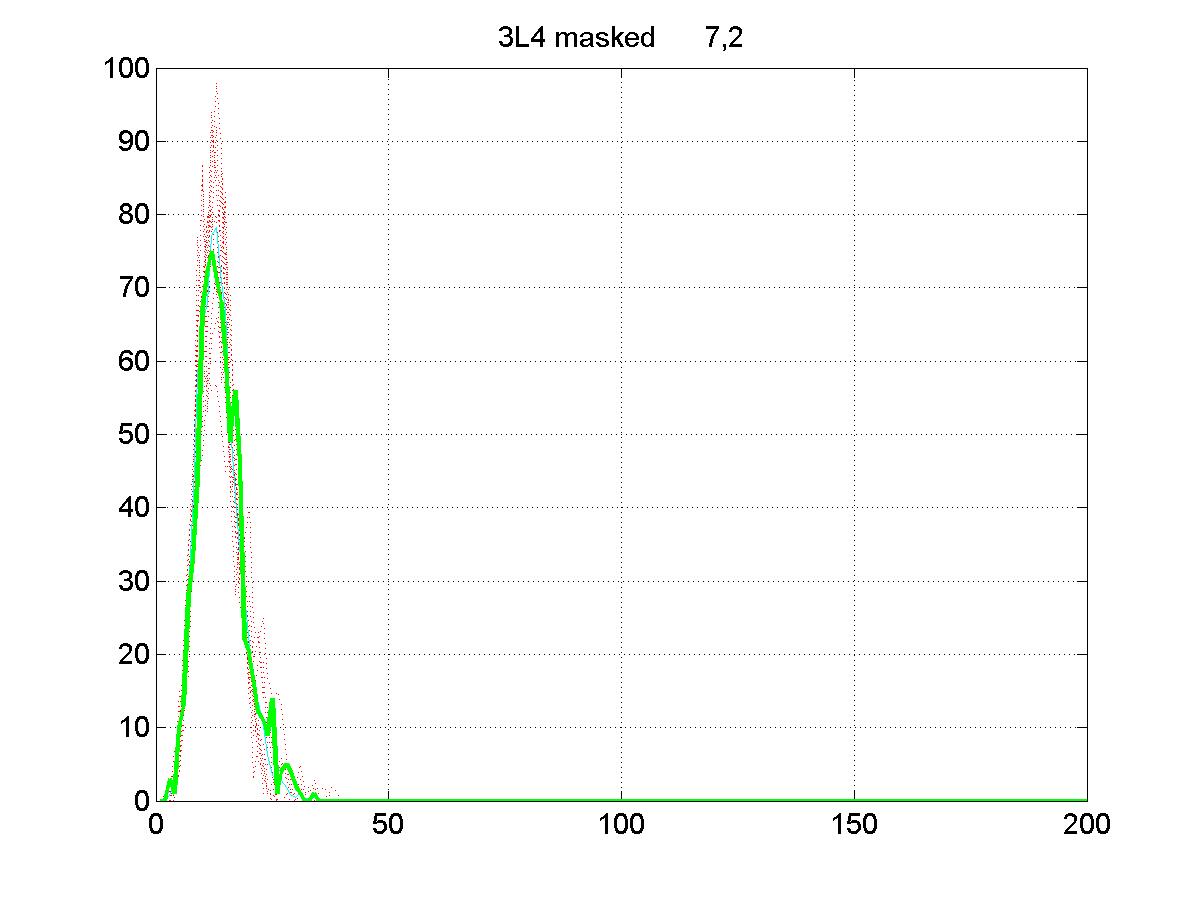

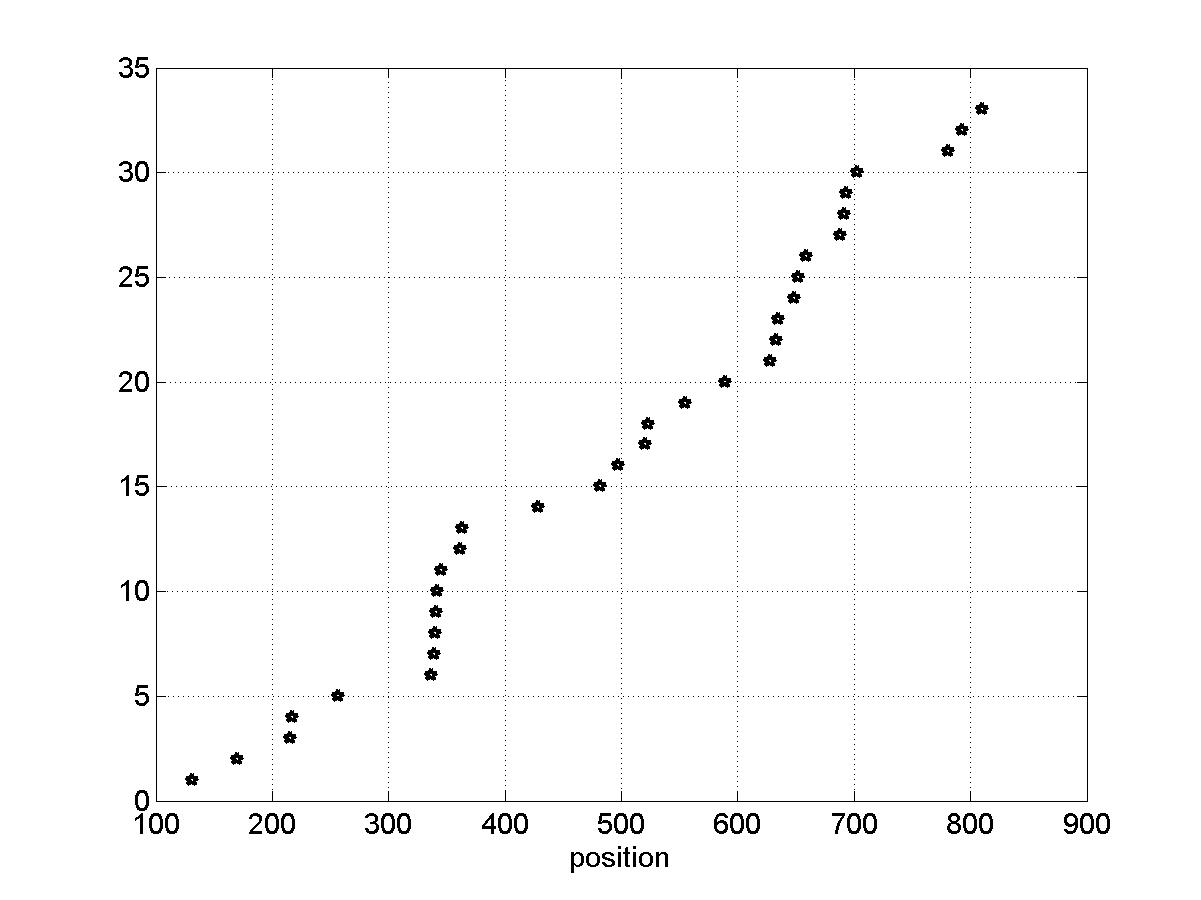


**Figure S17: Similar word distribution and spatial clustering for masked NCNR region 3L4, (m,mim)=(7,2).**


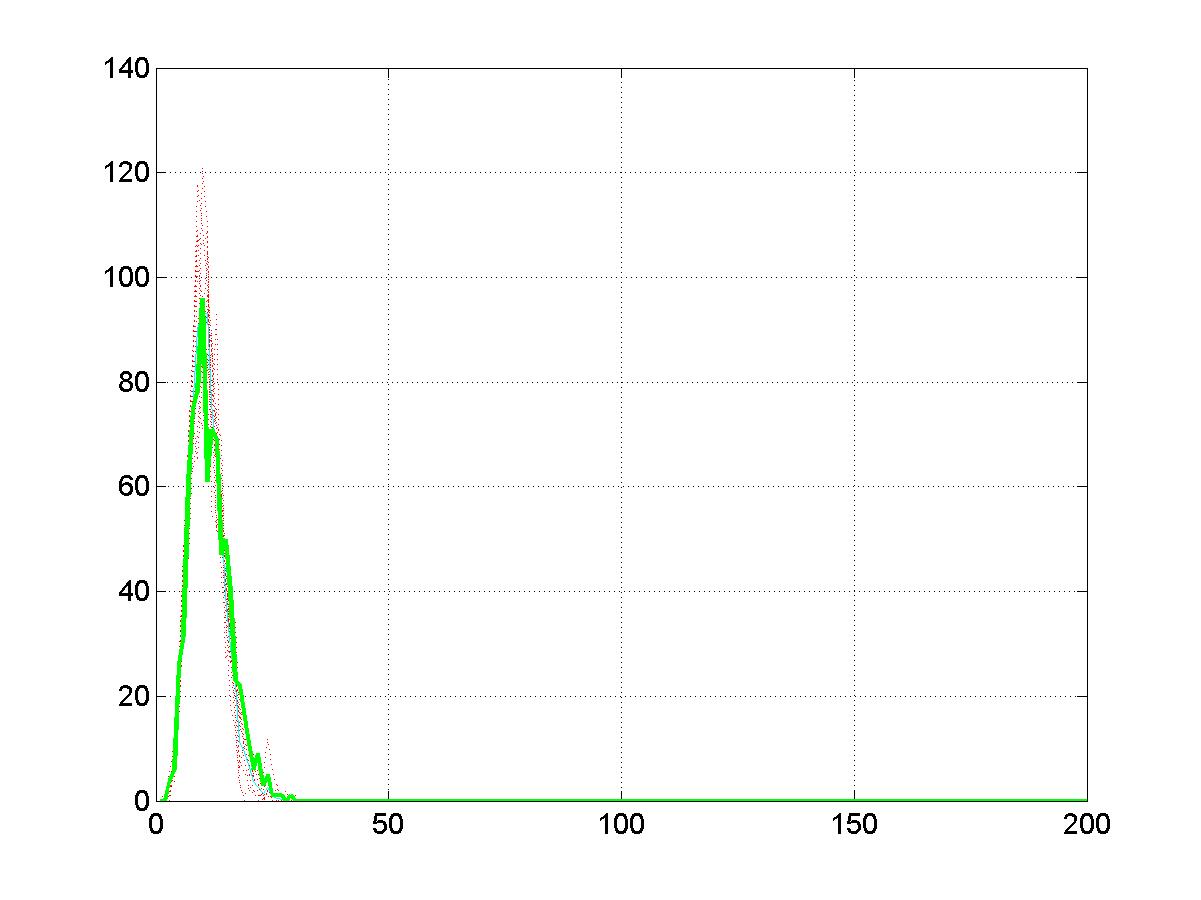

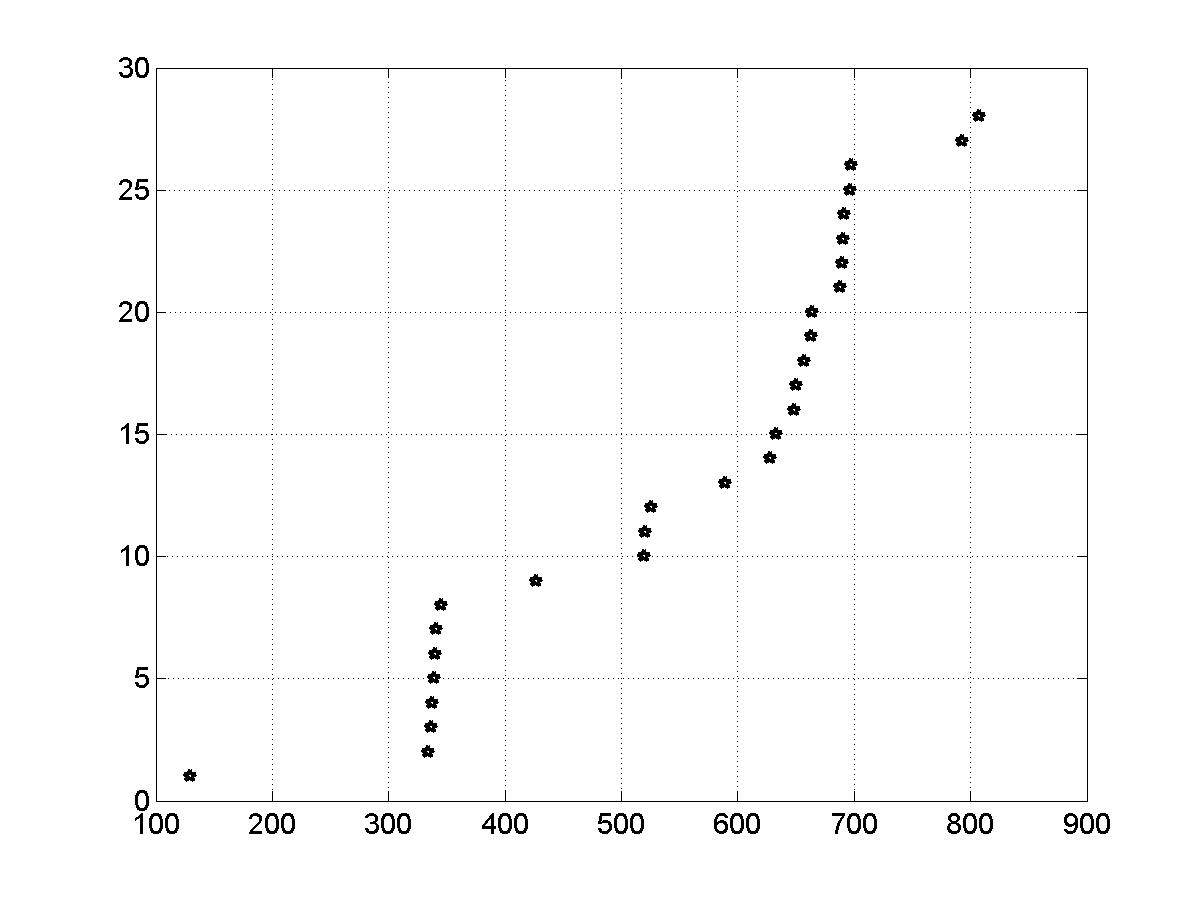


**Figure S18: Similar word distribution and spatial clustering for masked NCNR region 3L4, (m,mim)=(9,3).**


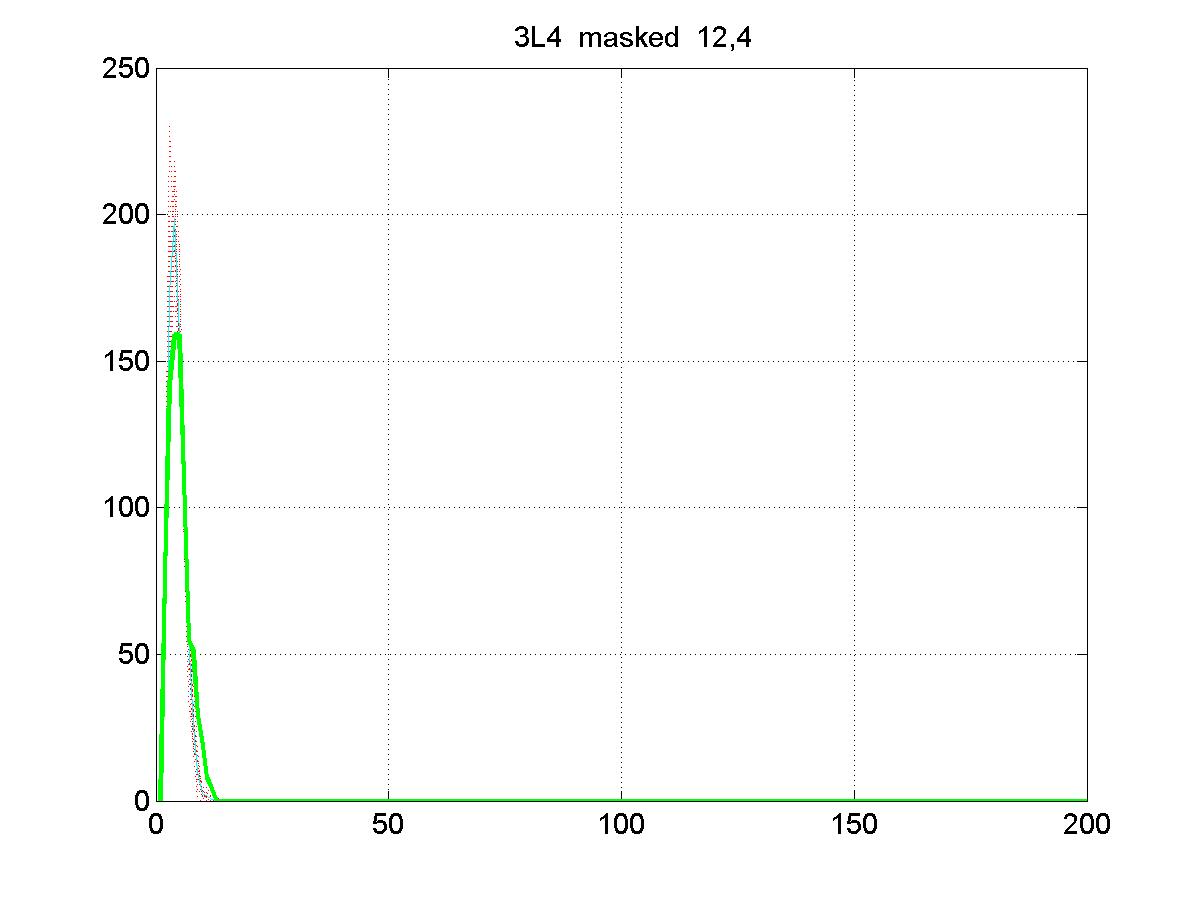

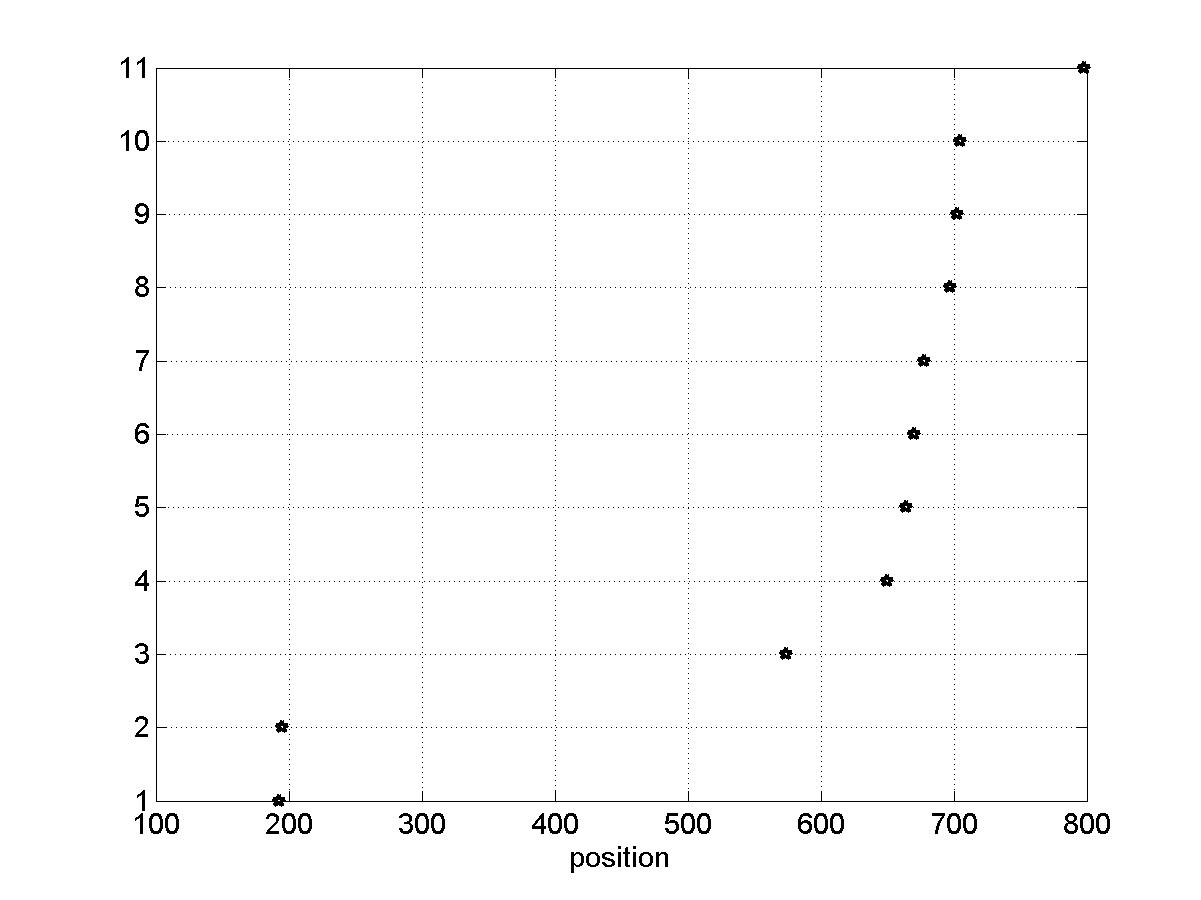


**Figure S19: Similar word distribution and spatial clustering for masked NCNR region 3L4, (m,mim)=(12,4).**
