# Supplementary material for: Some statistical properties of regulatory DNA sequences, and their use in predicting regulatory regions in the Drosophila genome: the fluffy-tail test
Source: BMC Bioinformatics. 2005 Apr 27;6:109. doi: 10.1186/1471-2105-6-109 (PMC1127108; doi:10.1186/1471-2105-6-109)
Supplement: Additional File 10 — Contains the Figures showing fluffiness and spatial clustering of similar words for knirps regulatory region [file 1471-2105-6-109-S10.doc]

# Supplementary Materials to the manuscript 'Some statistical properties of regulatory DNA sequences, and their use in predicting regulatory regions in the Drosophila genome: the fluffy-tail test.' *Irina Abnizova, Klaudia Walter, Rene te Boekhorst and Walter R. Gilks*

Supplementary F, CV for knirps cis-regulatory region

Table s3: F and CV for knirps regulatory region for different values (m,mim).

| m,mim | F | CV |
| --- | --- | --- |
| 3,0 | 11.18 | 0.78 |
| 5,1 | 8.65 | 0.68 |
| 7,2 | 10.11 | 0.78 |
| 9,3 | 16.4 | 0.56 |
| 12,4 | 23.5 | 0.90 |


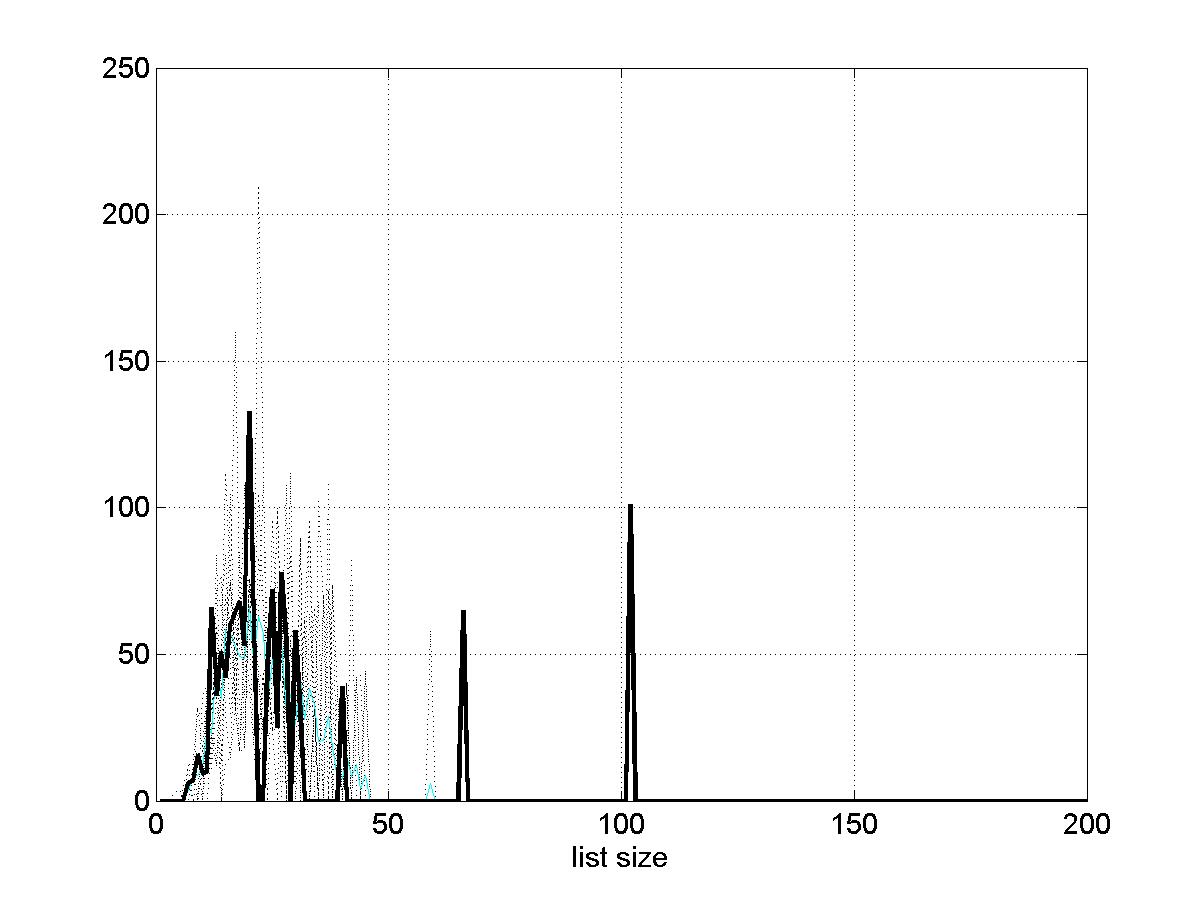

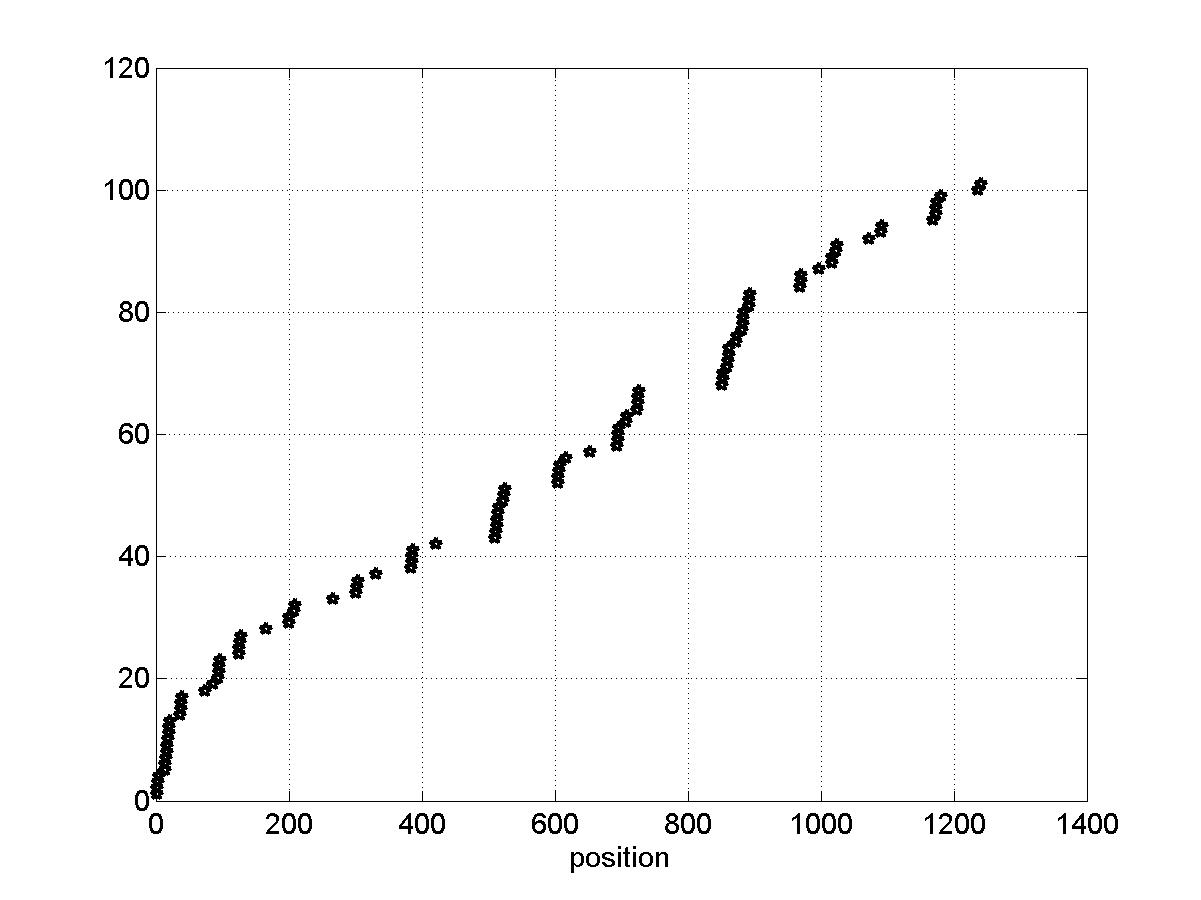


**Figure S20: Similar word distribution and spatial clustering for knirps regulatory region**

**(m,mim)=(3,0).**


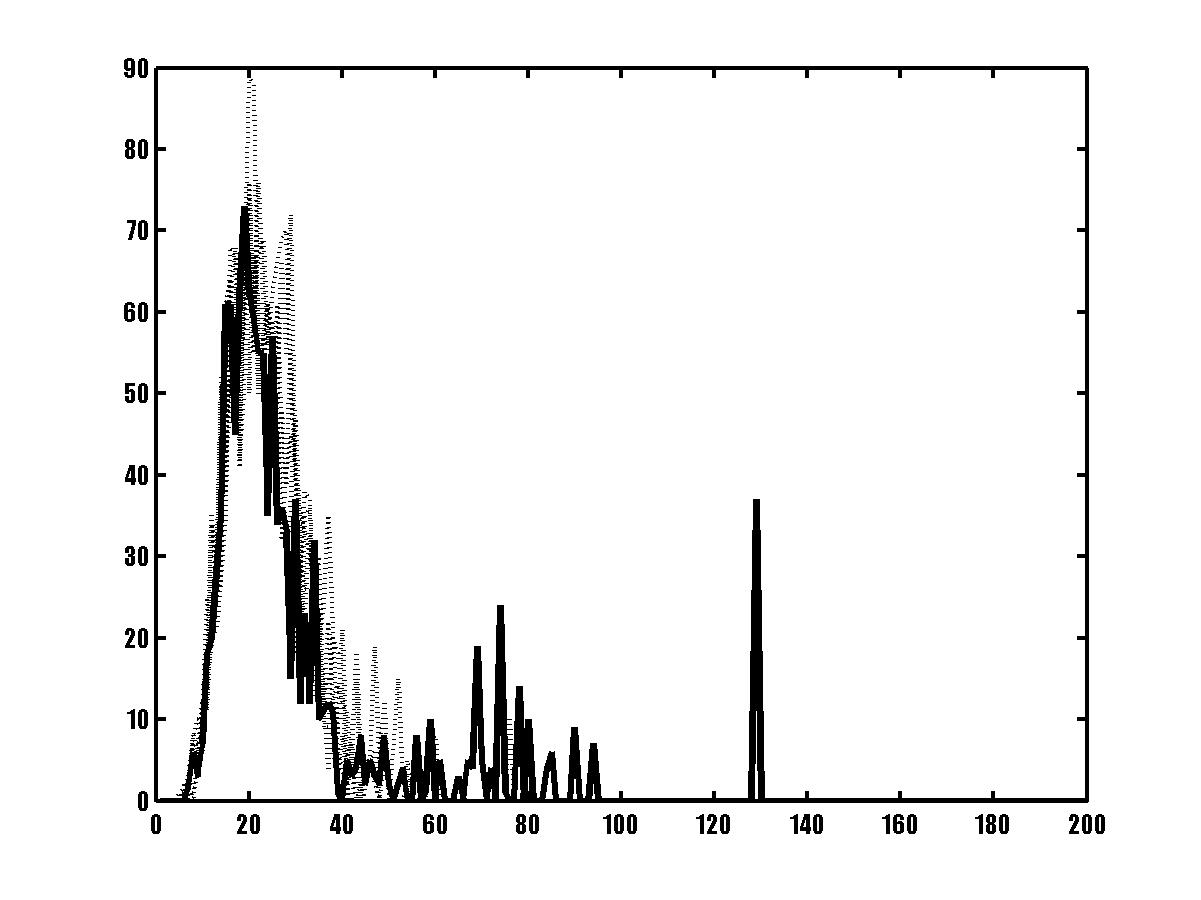

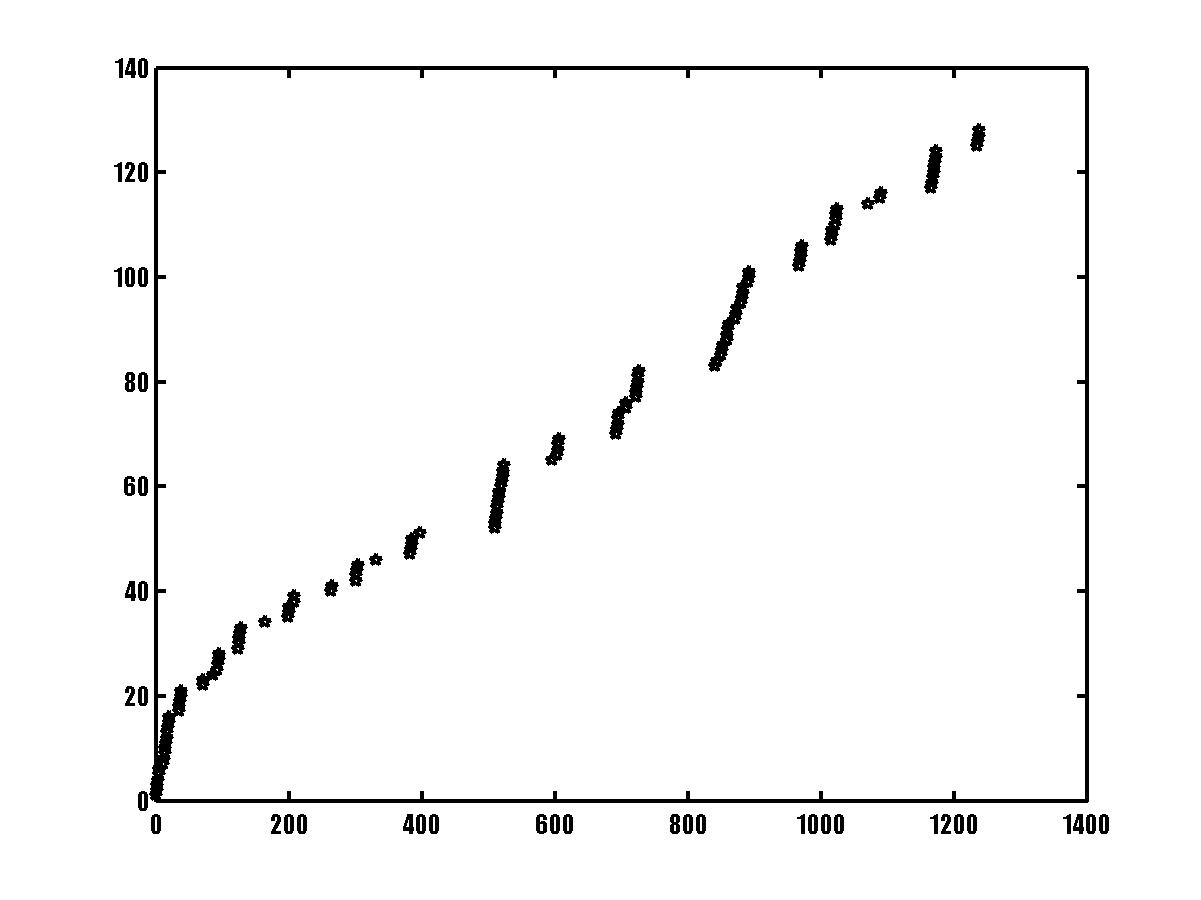


**Figure S21: Similar word distribution and spatial clustering for knirps regulatory region**

**(m,mim)=(5,1).**


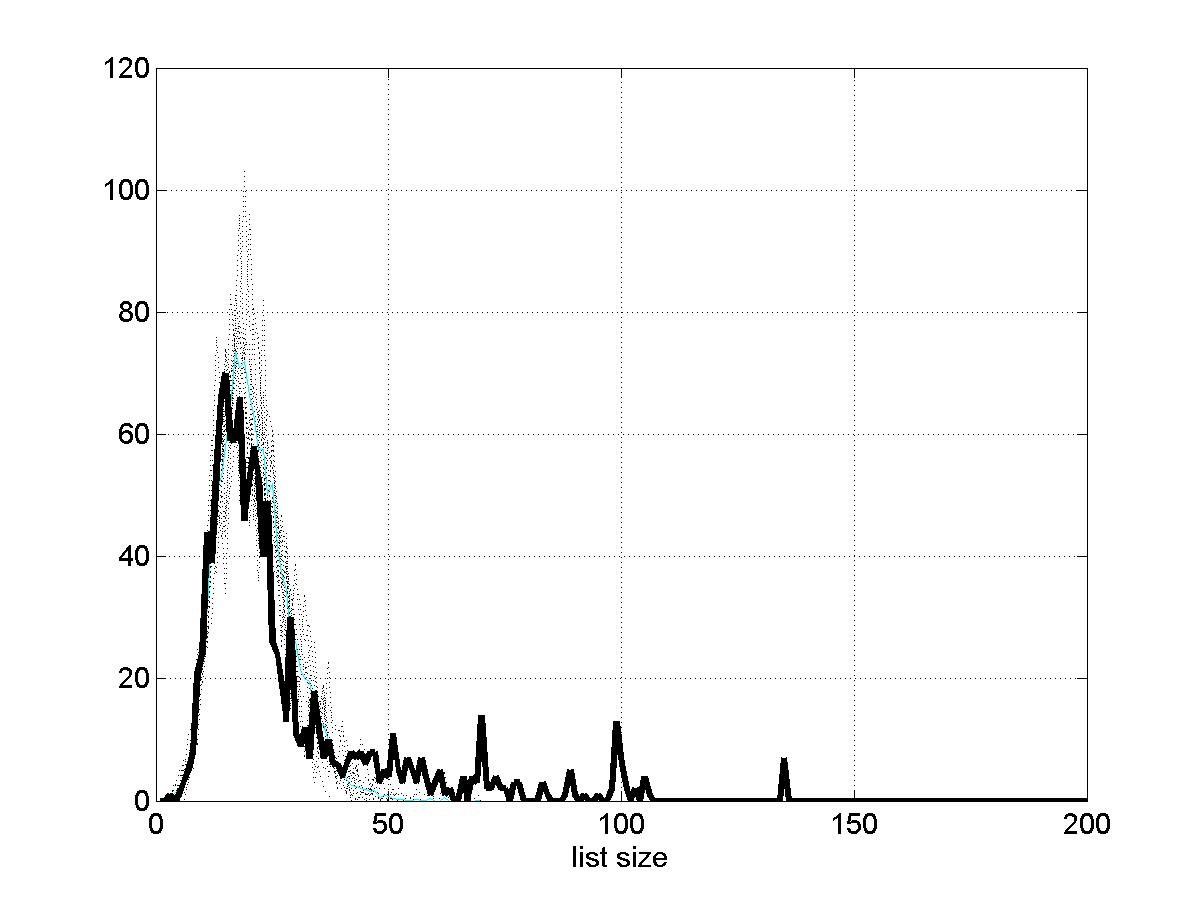

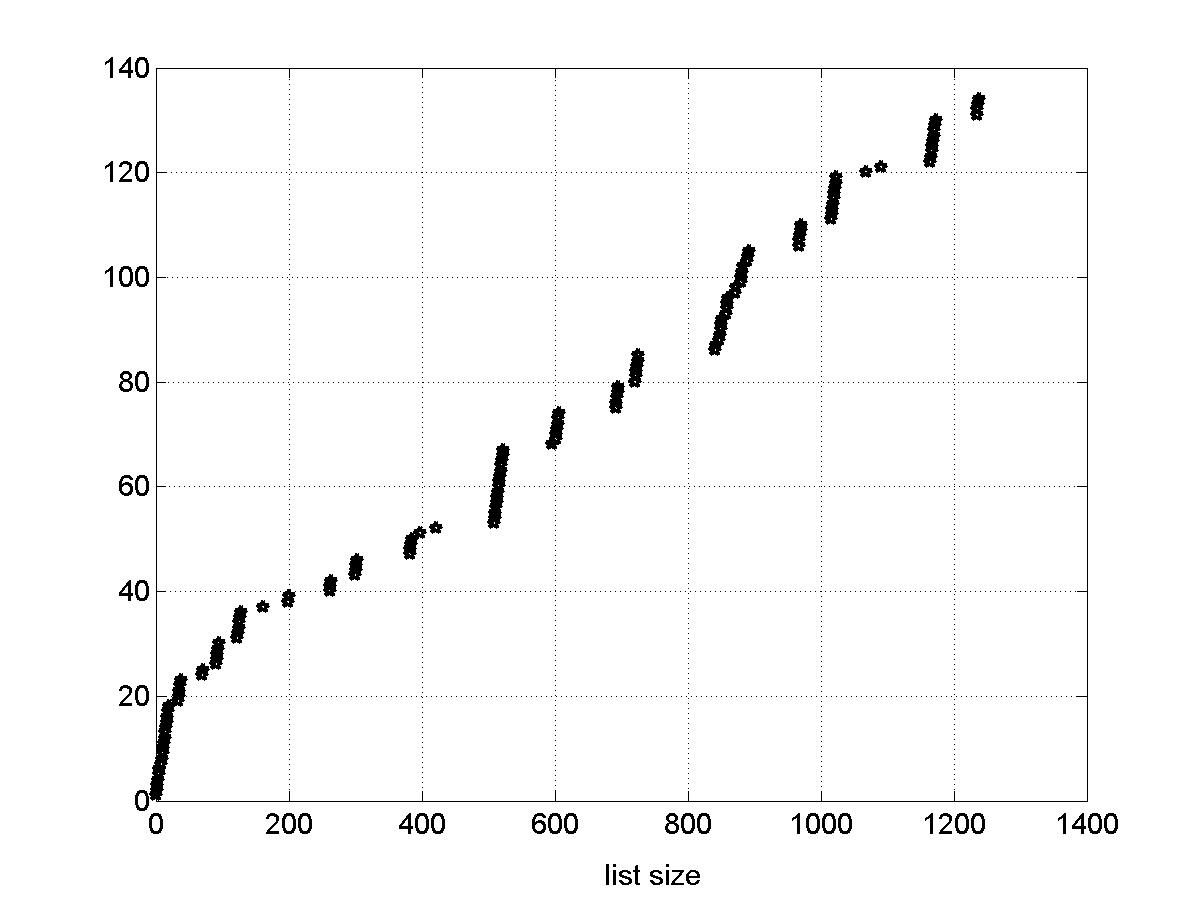


**Figure S22: Similar word distribution and spatial clustering for knirps regulatory region**

**(m,mim)=(7,2).**


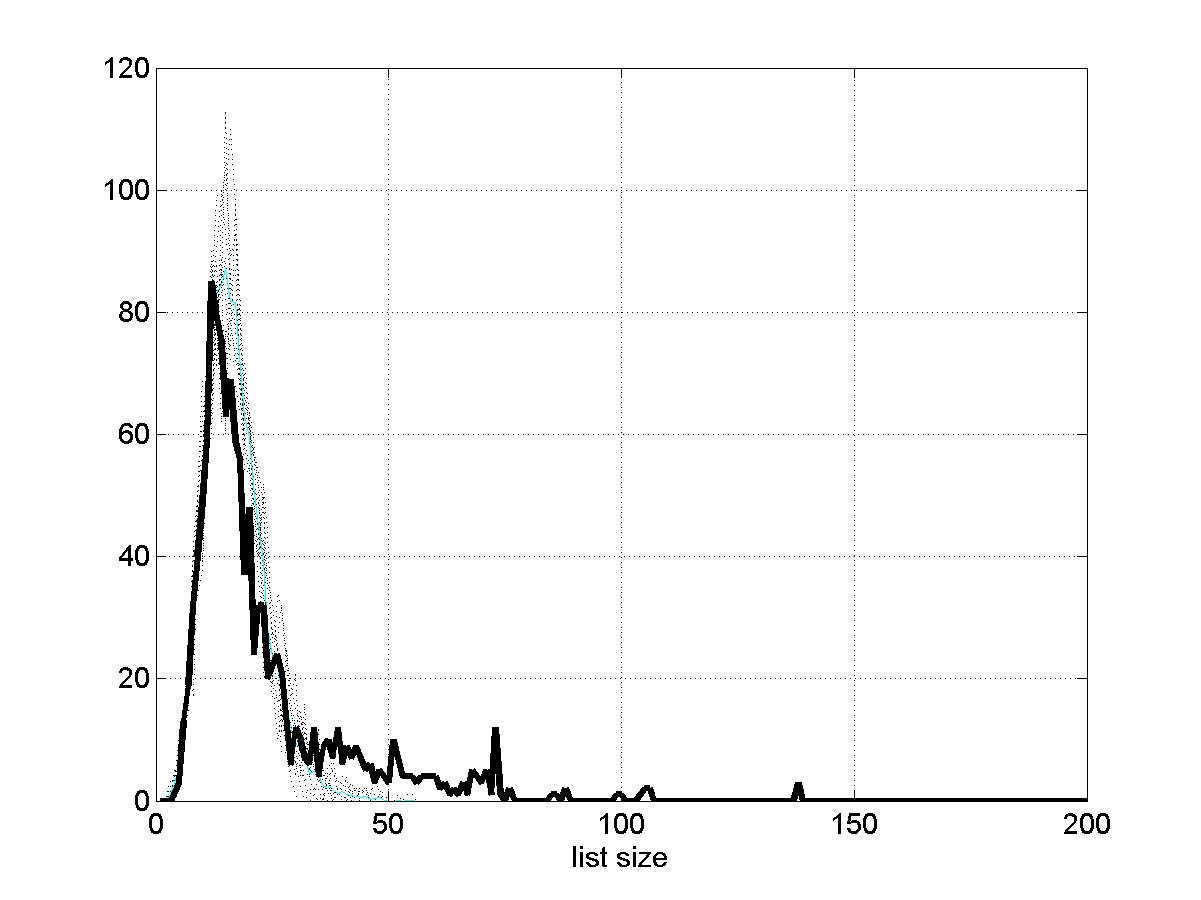

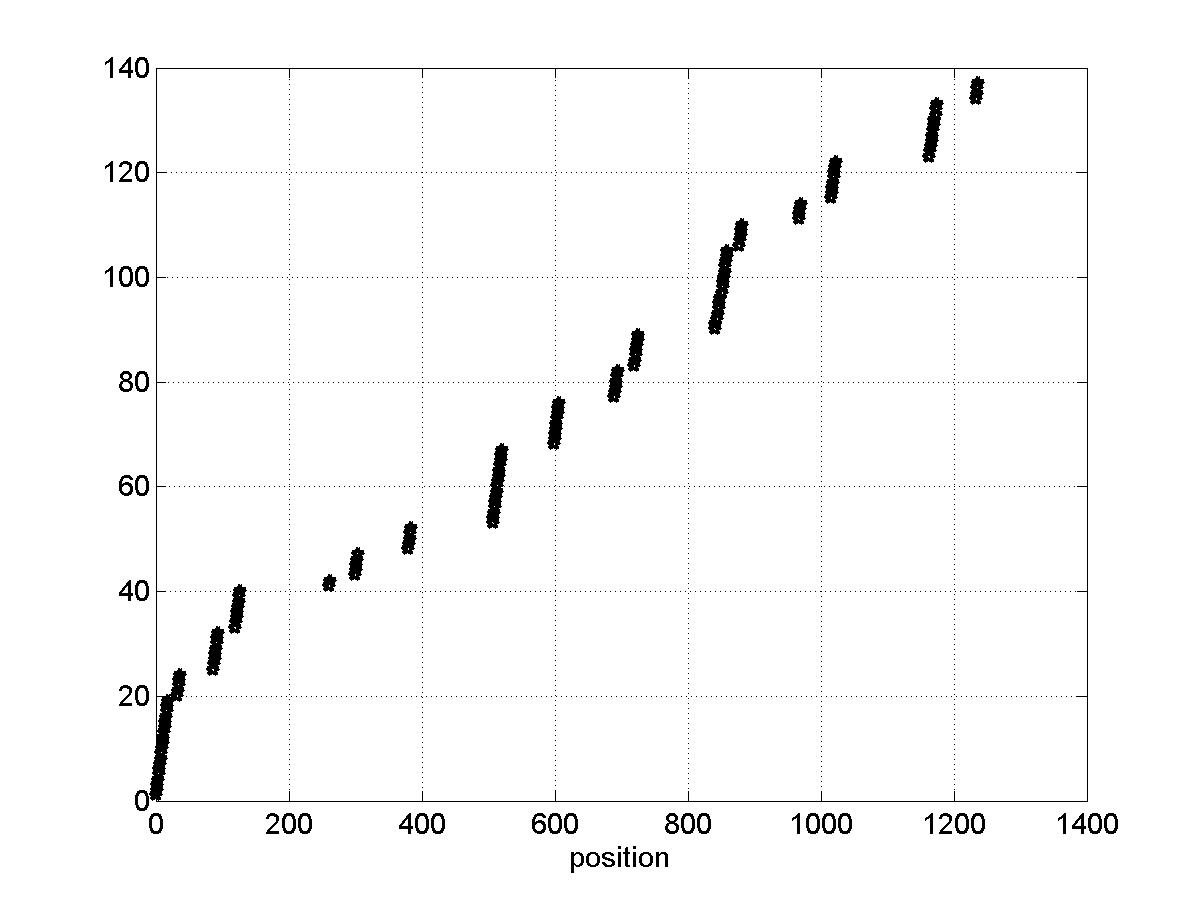


**Figure S23: Similar word distribution and spatial clustering for knirps regulatory region**

**(m,mim)=(9,3).**


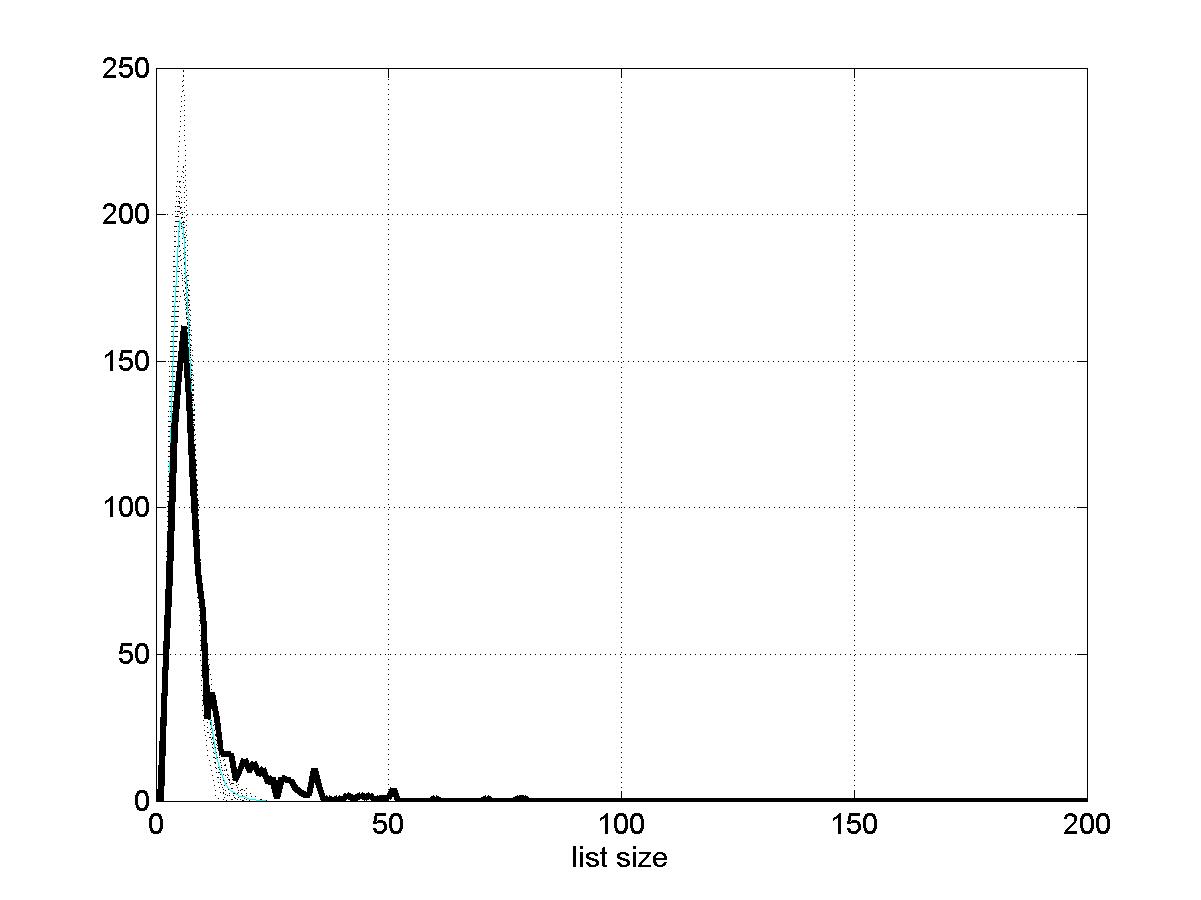

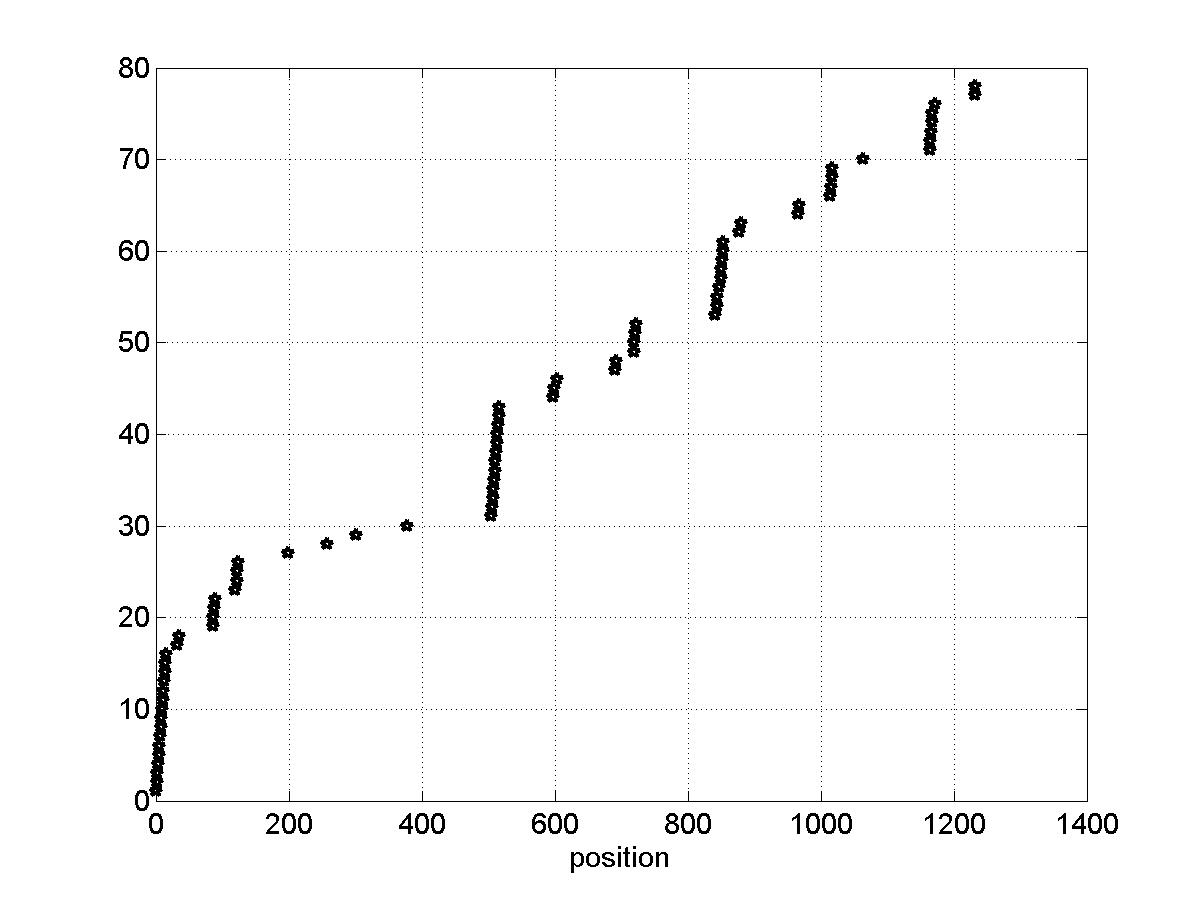


**Figure S24: Similar word distribution and spatial clustering for knirps regulatory region**

**(m,mim)=(12,4).**
