# Supplementary material for: Some statistical properties of regulatory DNA sequences, and their use in predicting regulatory regions in the Drosophila genome: the fluffy-tail test
Source: BMC Bioinformatics. 2005 Apr 27;6:109. doi: 10.1186/1471-2105-6-109 (PMC1127108; doi:10.1186/1471-2105-6-109)
Supplement: Additional File 11 — Contains the Figures showing fluffiness and spatial clustering of similar words for abdominantA regulatory region. [file 1471-2105-6-109-S11.doc]

# Supplementary Materials to the manuscript 'Some statistical properties of regulatory DNA sequences, and their use in predicting regulatory regions in the Drosophila genome: the fluffy-tail test.' *Irina Abnizova, Klaudia Walter, Rene te Boekhorst and Walter R. Gilks*

Supplementary F, CV for abdominantA regulatory region

Table s2: F and CV for abdominantA regulatory region for different values (m,mim).

| m,mim | F | CV |
| --- | --- | --- |
| 3,0 | 14.10 | 0.59 |
| 5,1 | 11.26 | 0.77 |
| 7,2 | 18.3 | 0.70 |
| 9,3 | 13.9 | 1.07 |
| 12,4 | 8.7 | 0.82 |


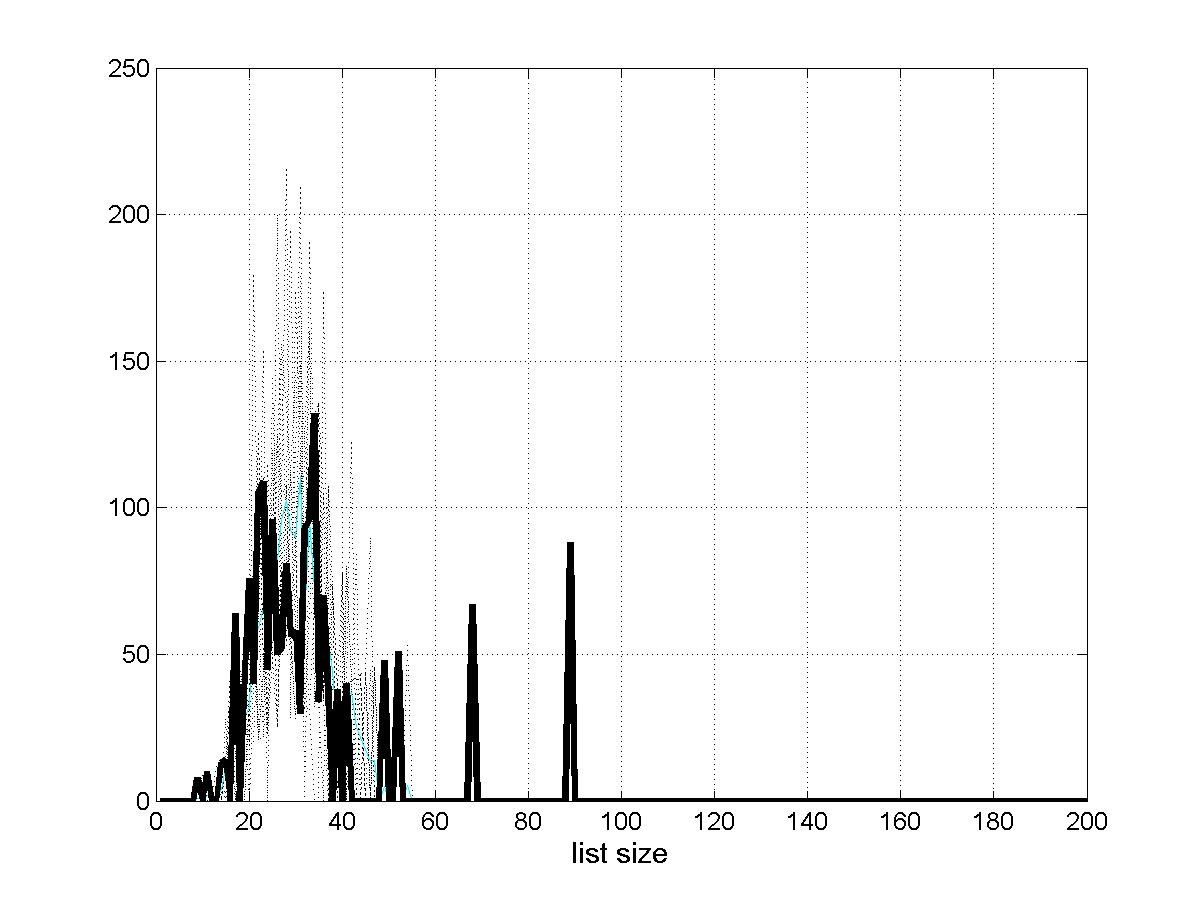

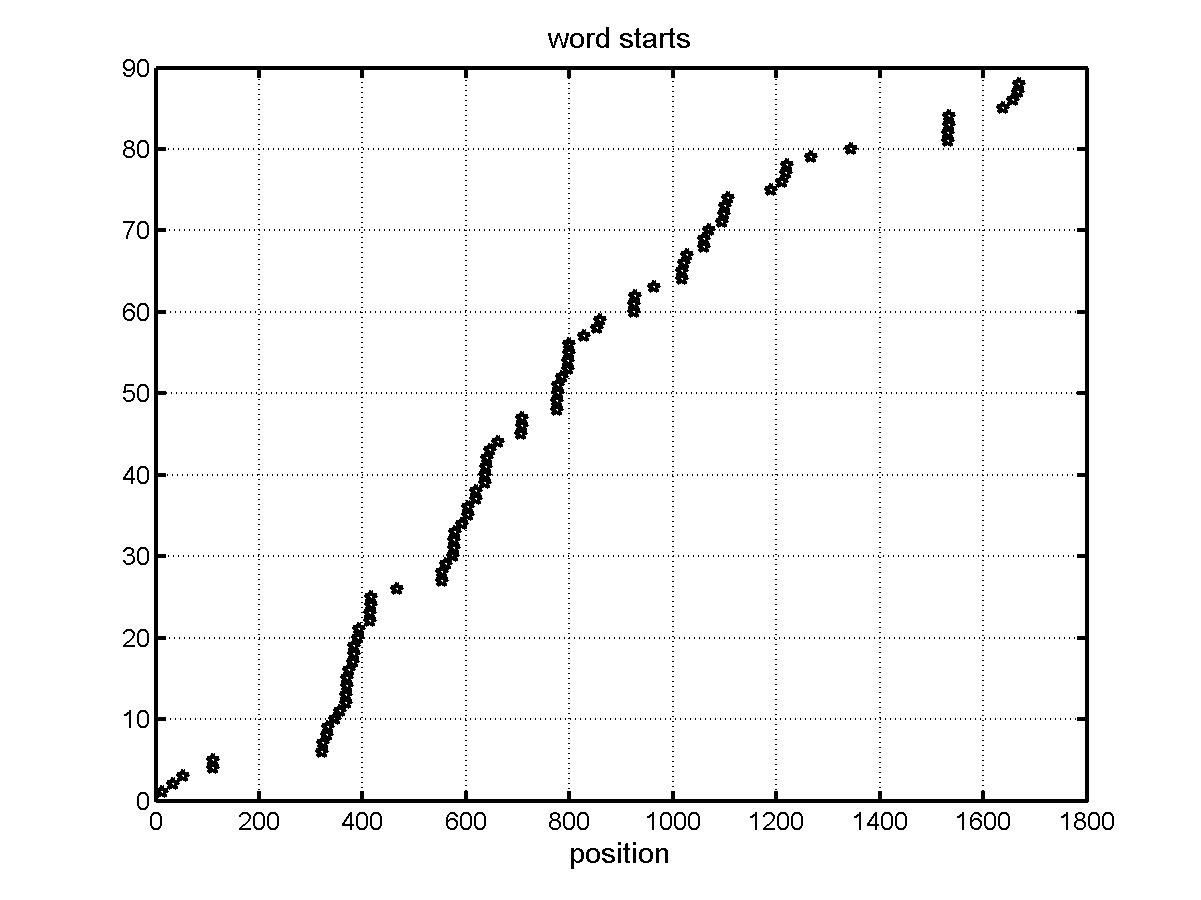


**Figure S31: Similar word distribution and spatial clustering for abdominantA regulatory region**

**(m,mim)=(3,0).**


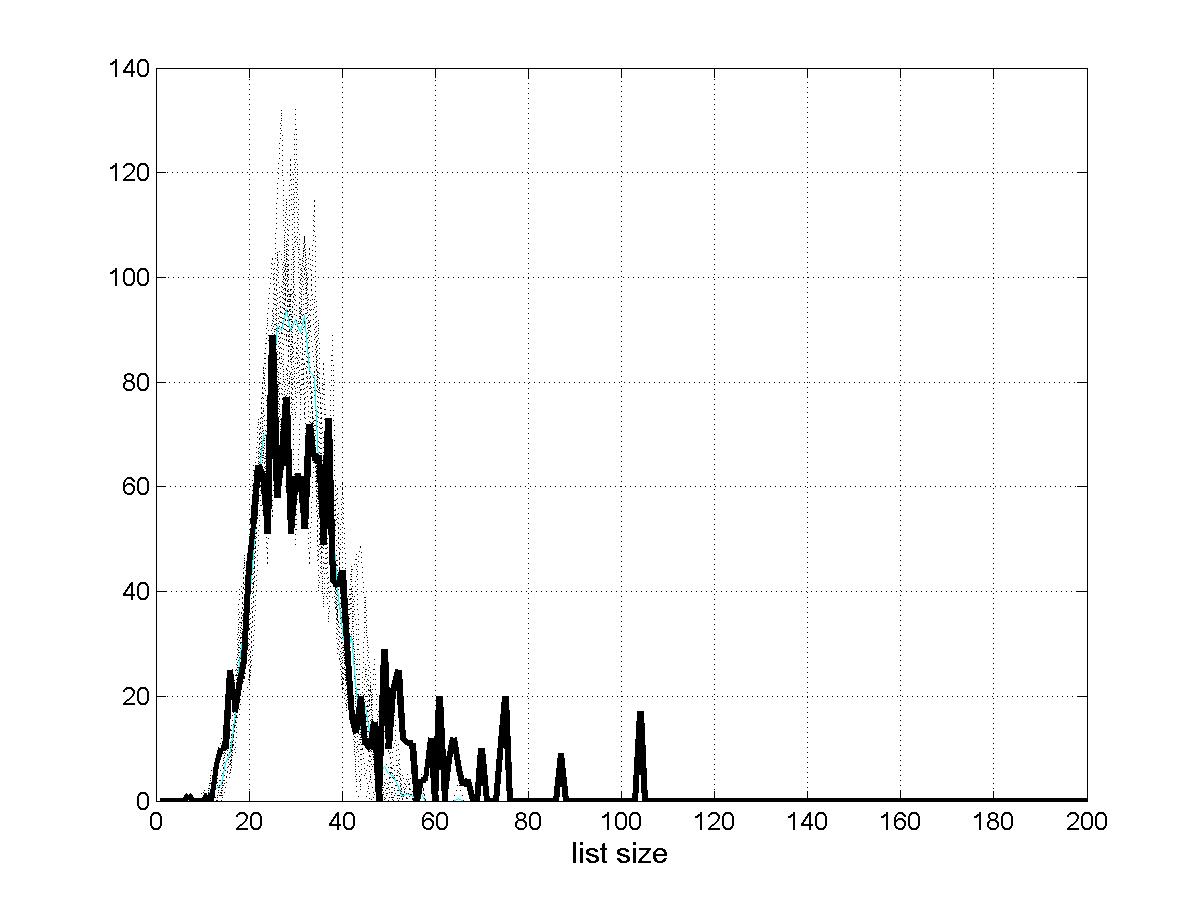

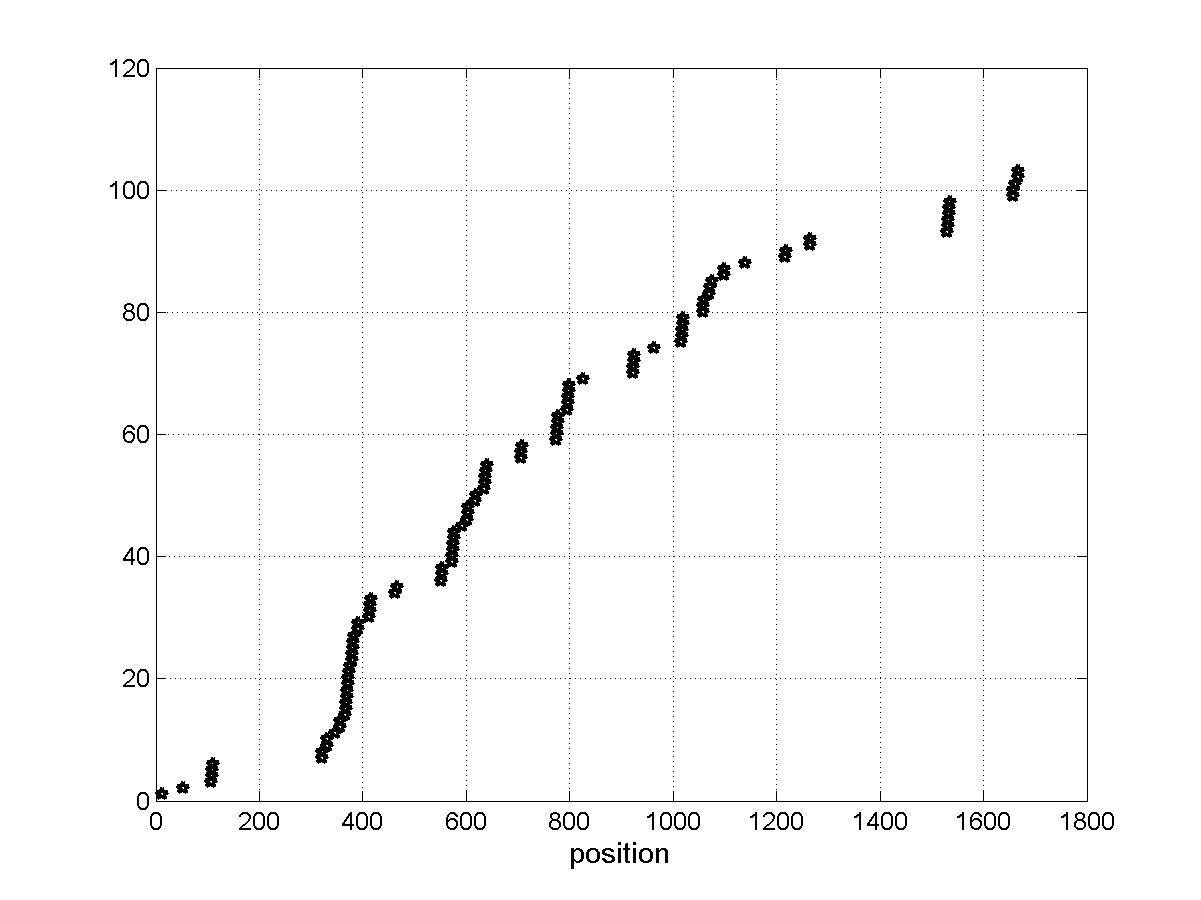


**Figure S32: Similar word distribution and spatial clustering for abdominantA regulatory region**

**(m,mim)=(5,1).**


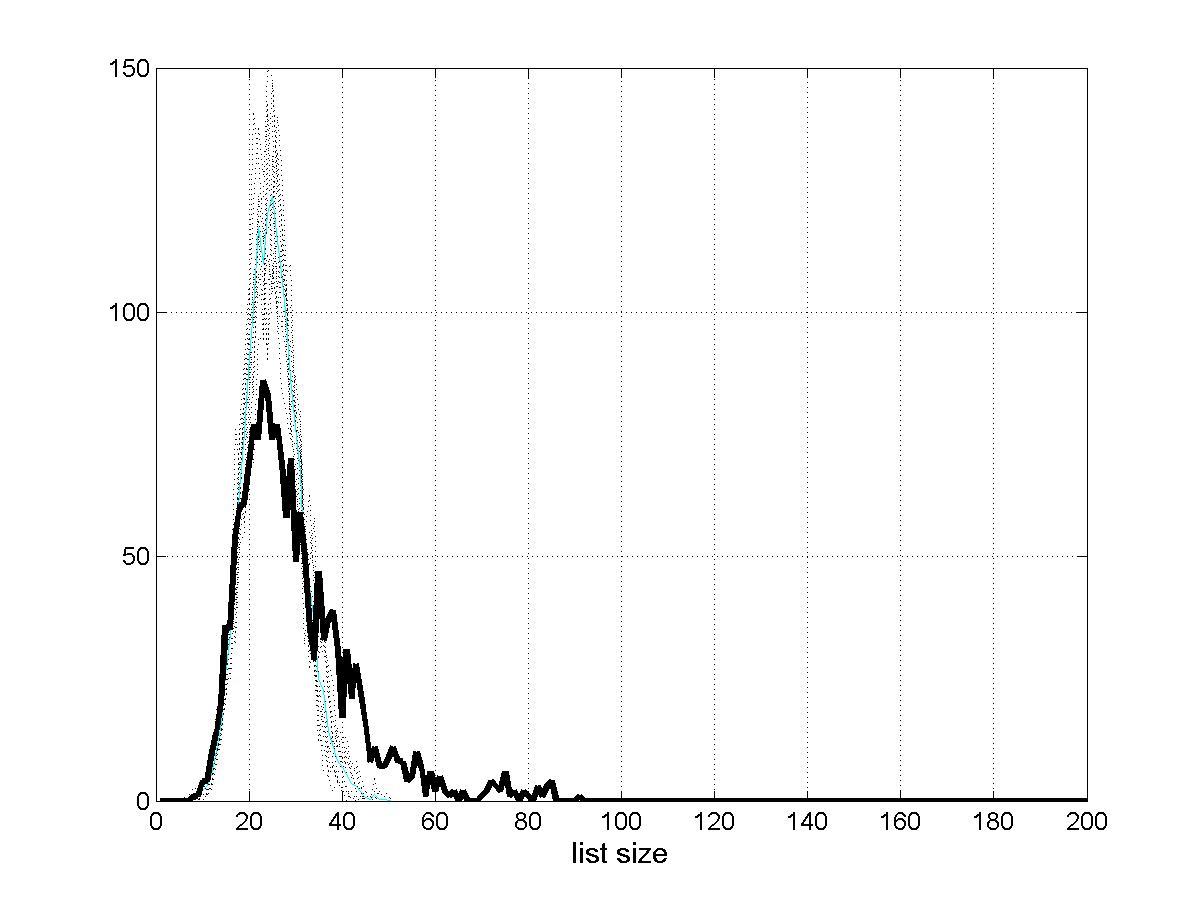

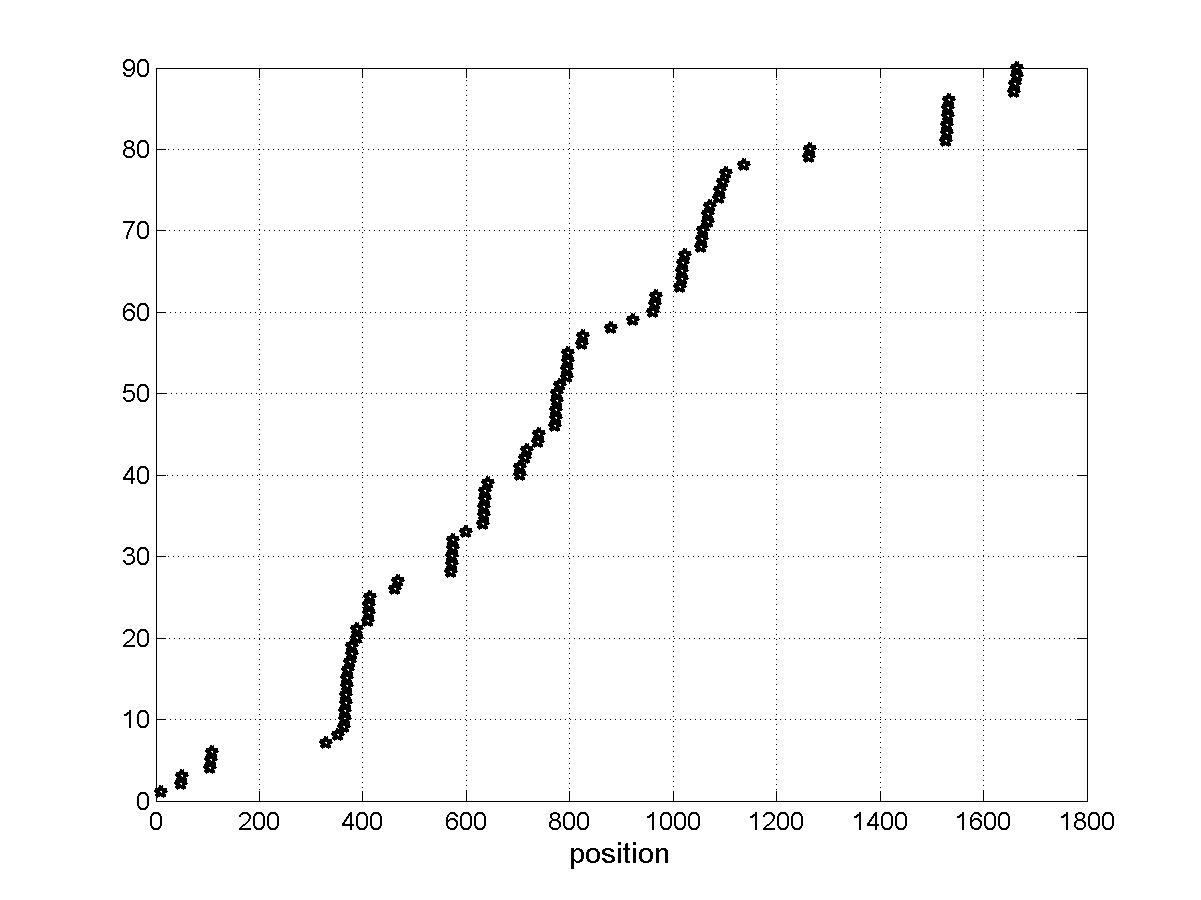


**Figure S33: Similar word distribution and spatial clustering for abdominantA regulatory region**

**(m,mim)=(7,2).**


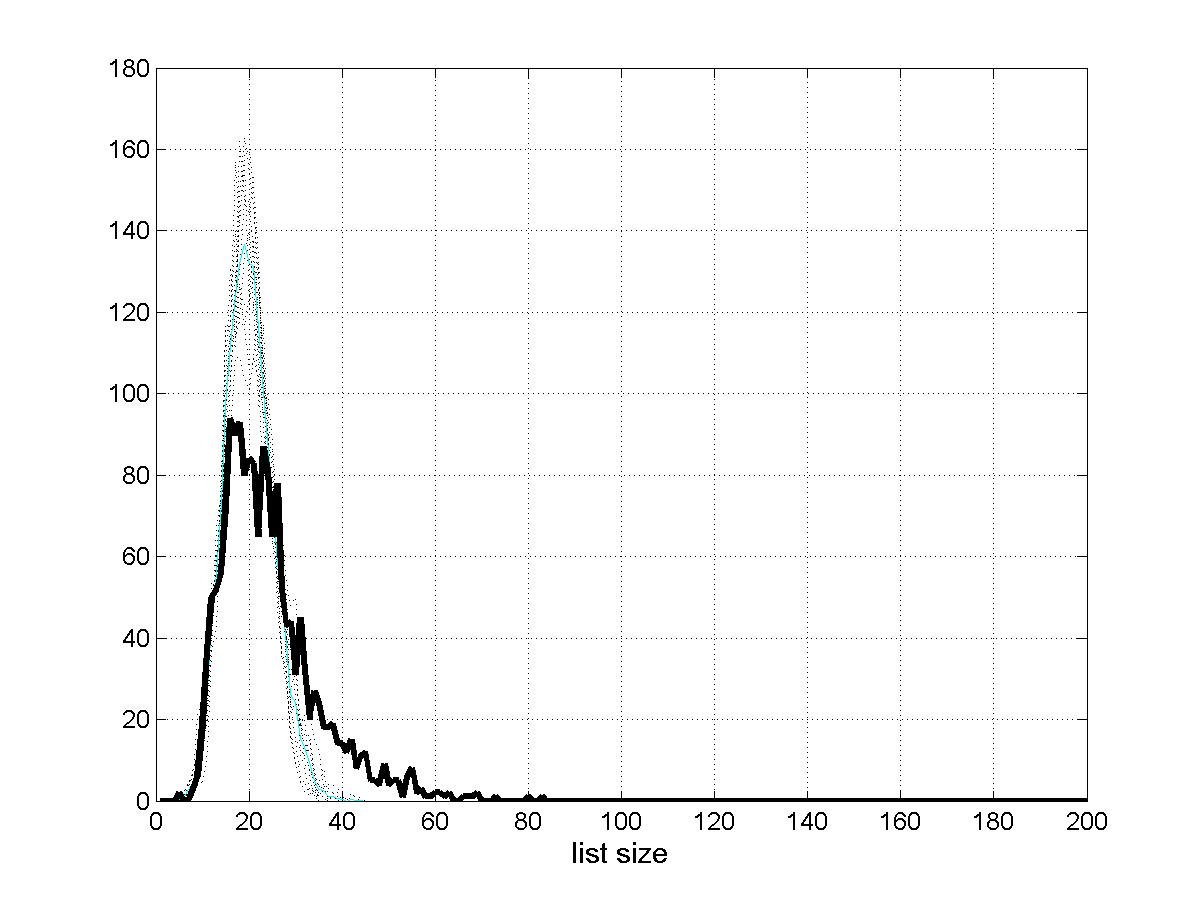

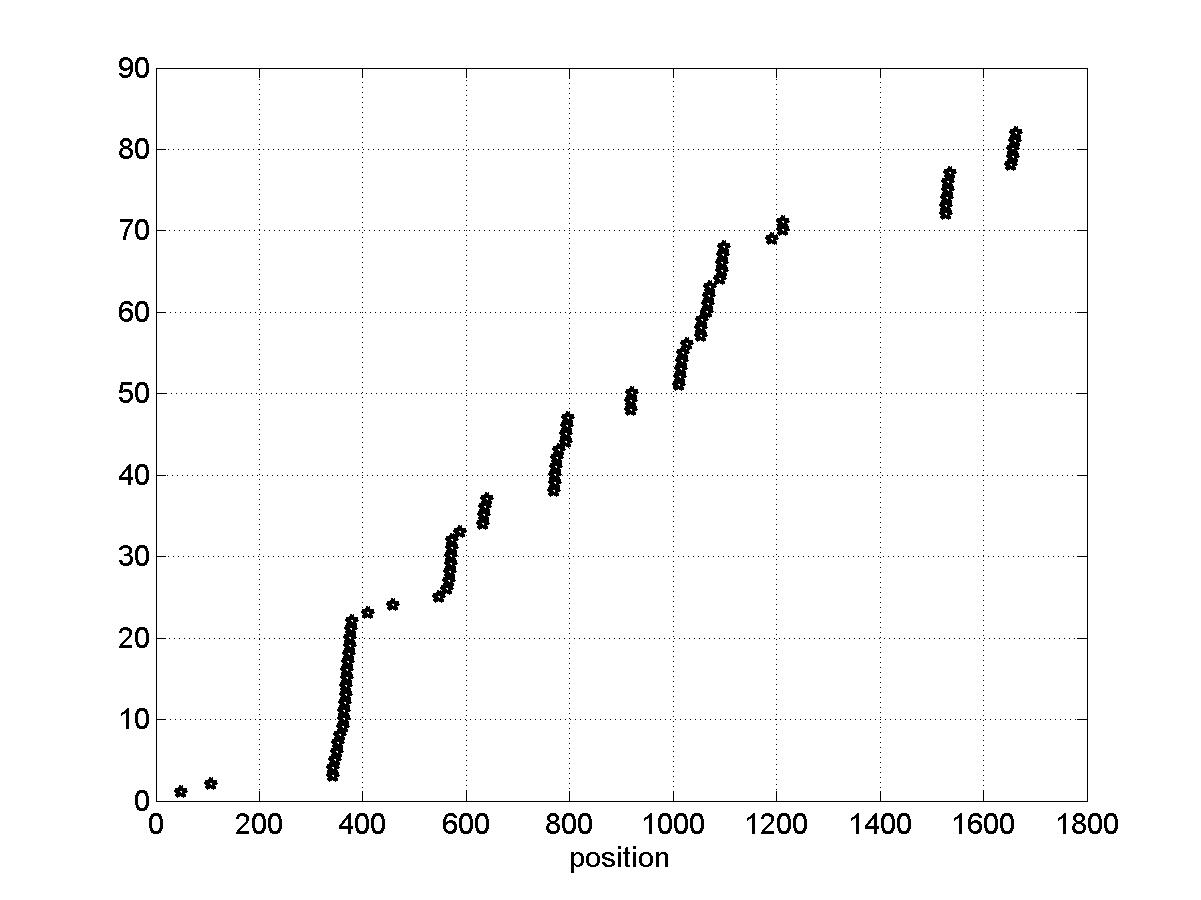


**Figure S34: Similar word distribution and spatial clustering for abdominantA regulatory region**

**(m,mim)=(9,3).**


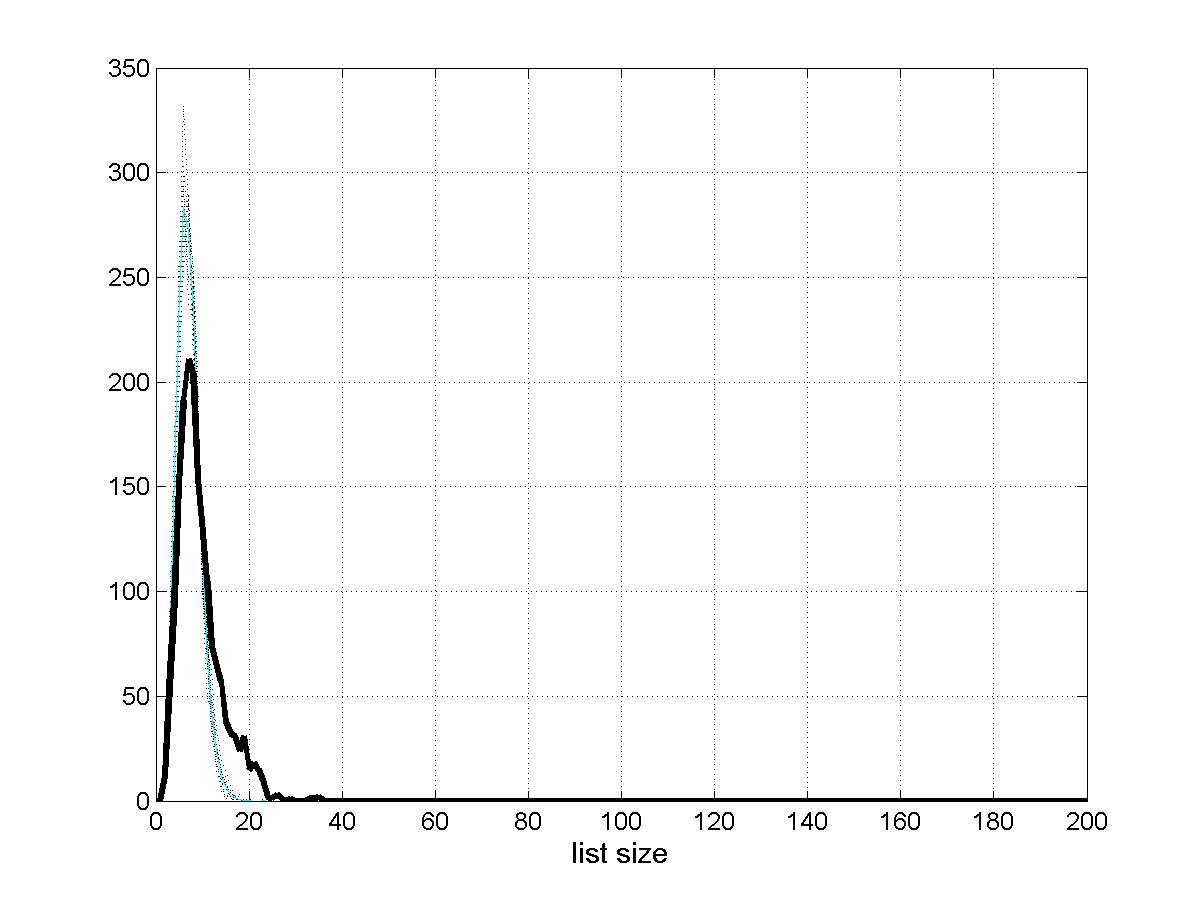

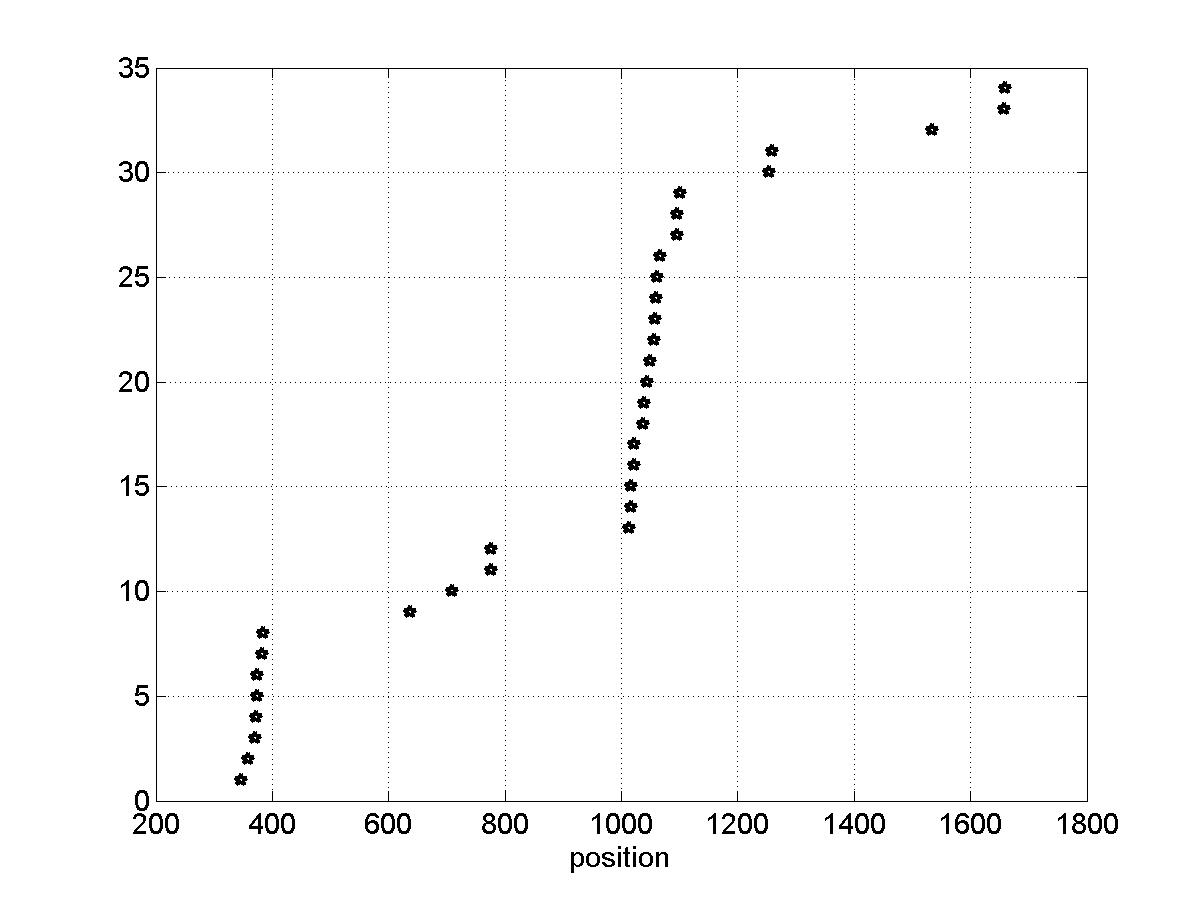


**Figure S35 Similar word distribution and spatial clustering for abdominantA regulatory region**

**(m,mim)=(12,4).**
