# Supplementary material for: Some statistical properties of regulatory DNA sequences, and their use in predicting regulatory regions in the Drosophila genome: the fluffy-tail test
Source: BMC Bioinformatics. 2005 Apr 27;6:109. doi: 10.1186/1471-2105-6-109 (PMC1127108; doi:10.1186/1471-2105-6-109)
Supplement: Additional File 12 — Contains the Figures showing fluffiness and spatial clustering of similar words for internal exon 2r4. [file 1471-2105-6-109-S12.doc]

# Supplementary Materials to the manuscript 'Some statistical properties of regulatory DNA sequences, and their use in predicting regulatory regions in the Drosophila genome: the fluffy-tail test.' *Irina Abnizova, Klaudia Walter, Rene te Boekhorst and Walter R. Gilks*

Supplementary, F and CV exon 2r4

Table s1: F and CV for exon 2r4 for different values (m,mim).

| m,mim | F | CV |
| --- | --- | --- |
| 3,0 | 1.6 | 0.52 |
| 5,1 | 0.85 | 0.69 |
| 7,2 | 2.0 | 0.62 |
| 8,2 | 1.6 | 0.64 |
| 9,3 | 1.91 | 0.55 |
| 12,4 | 1.18 | 0.33 |


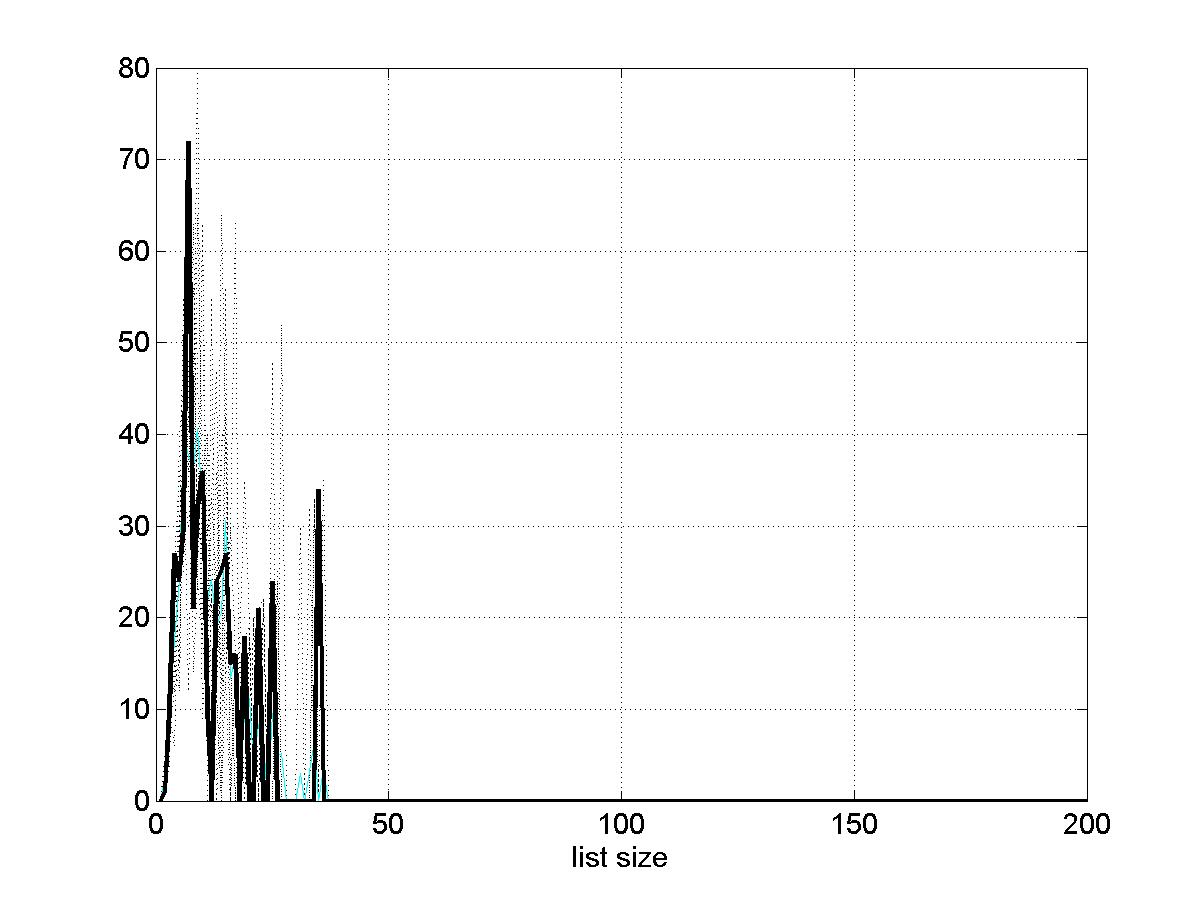

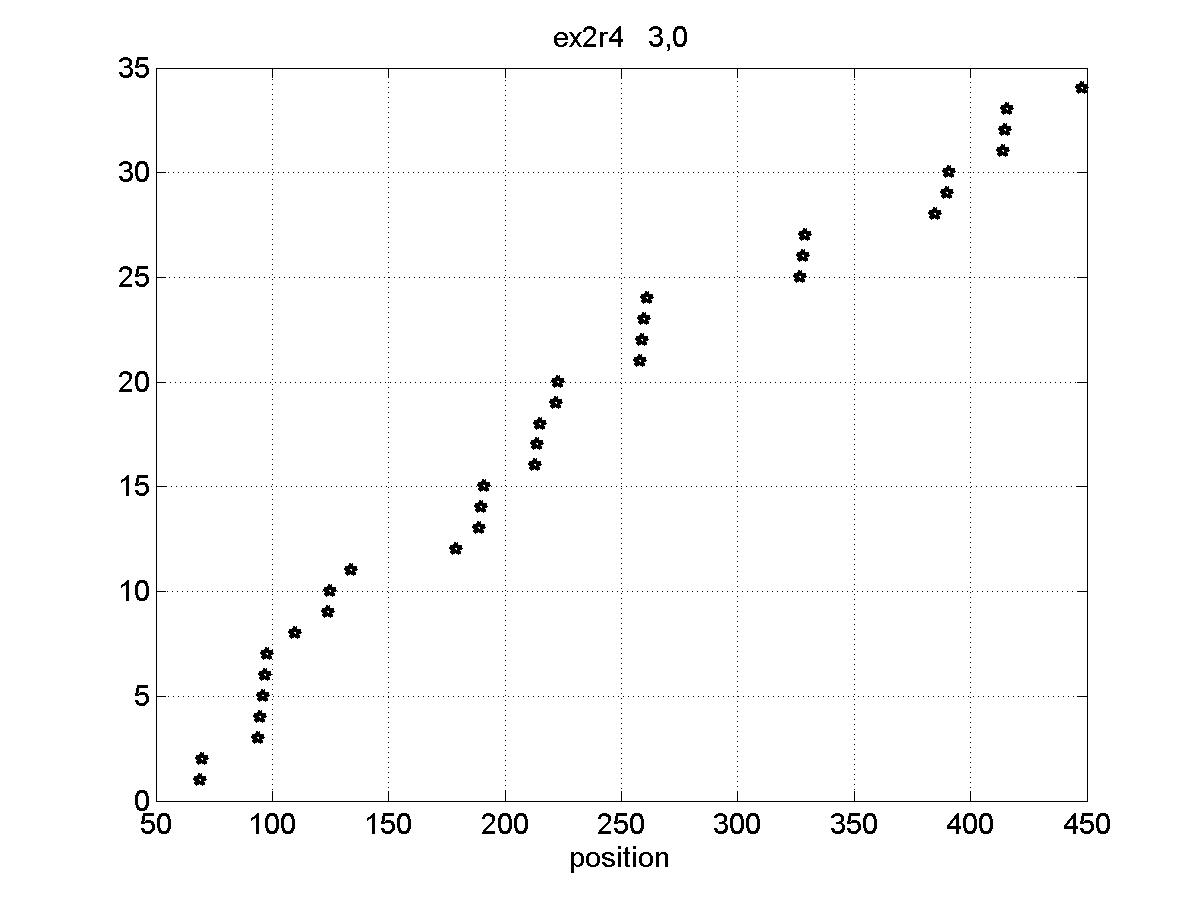


**Figure S25: Similar word distribution and spatial clustering for exon 2r4; (m,mim)=(3,0).**


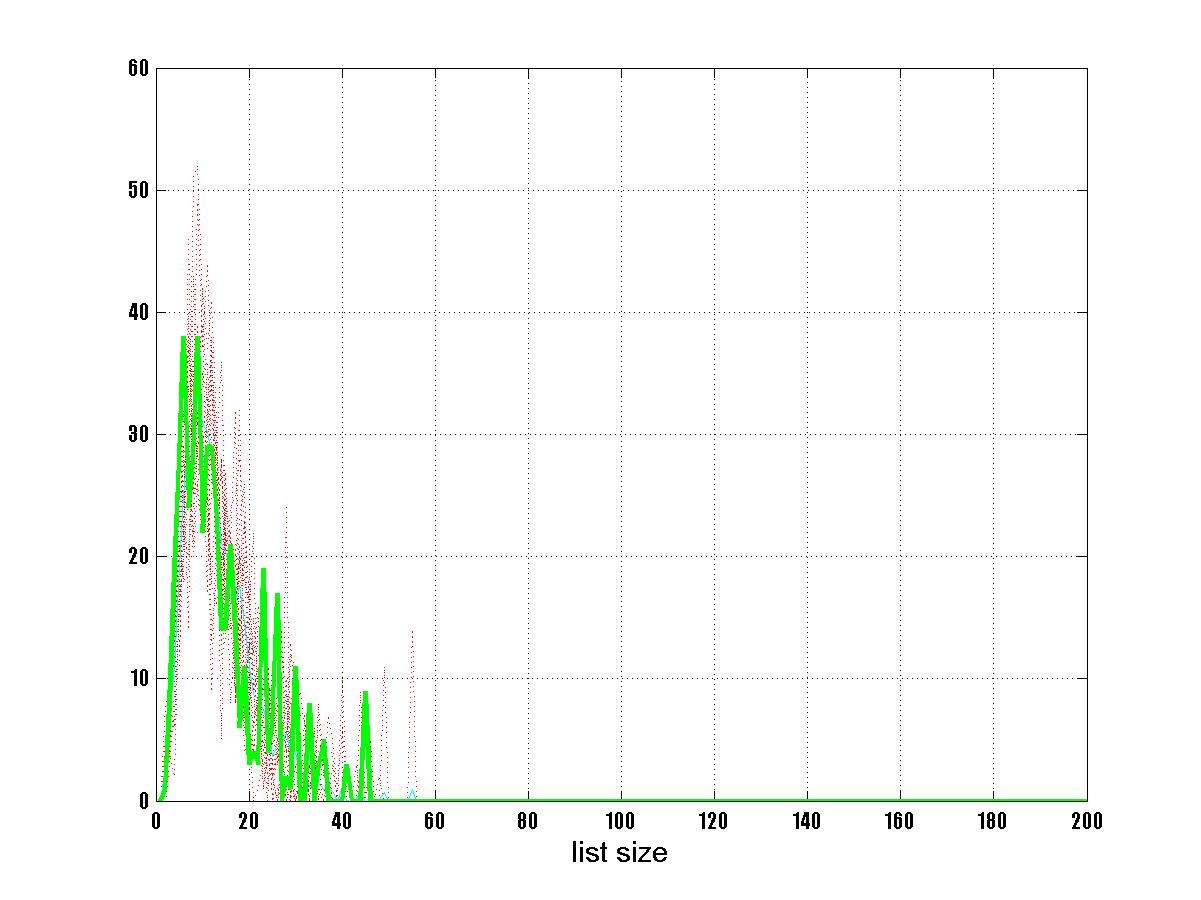

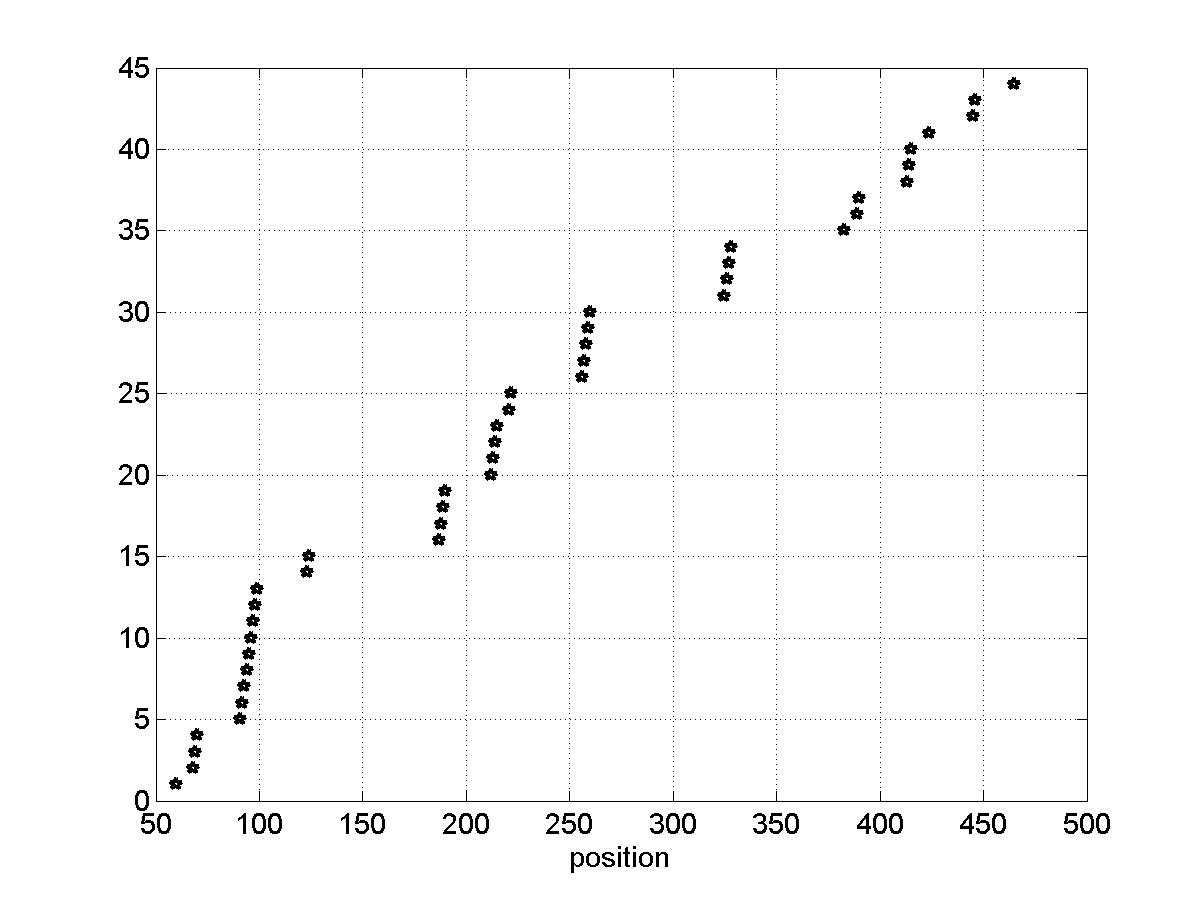


**Figure S26: Similar word distribution and spatial clustering for exon 2r4; (m,mim)=(5,1).**


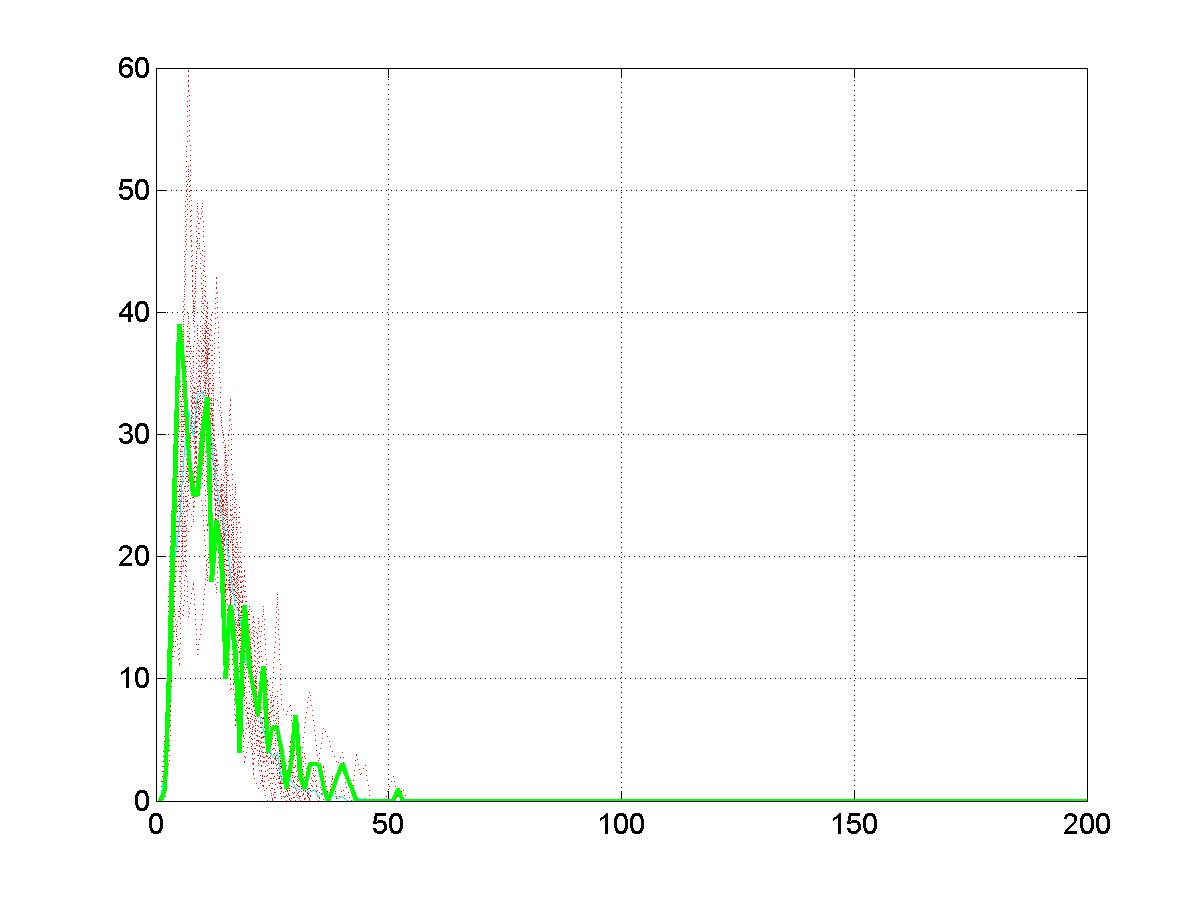

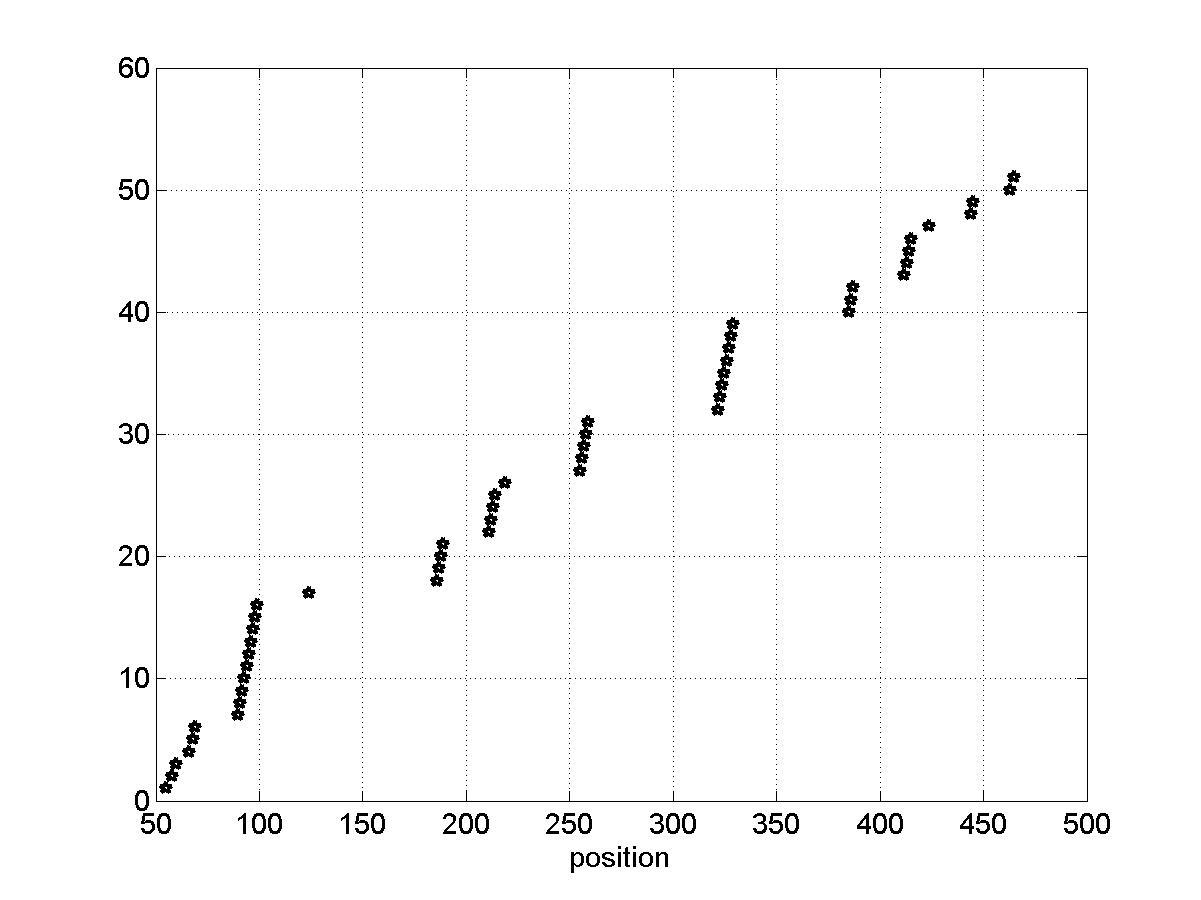


**Figure S27: Similar word distribution and spatial clustering for exon 2r4; (m,mim)=(7,2).**


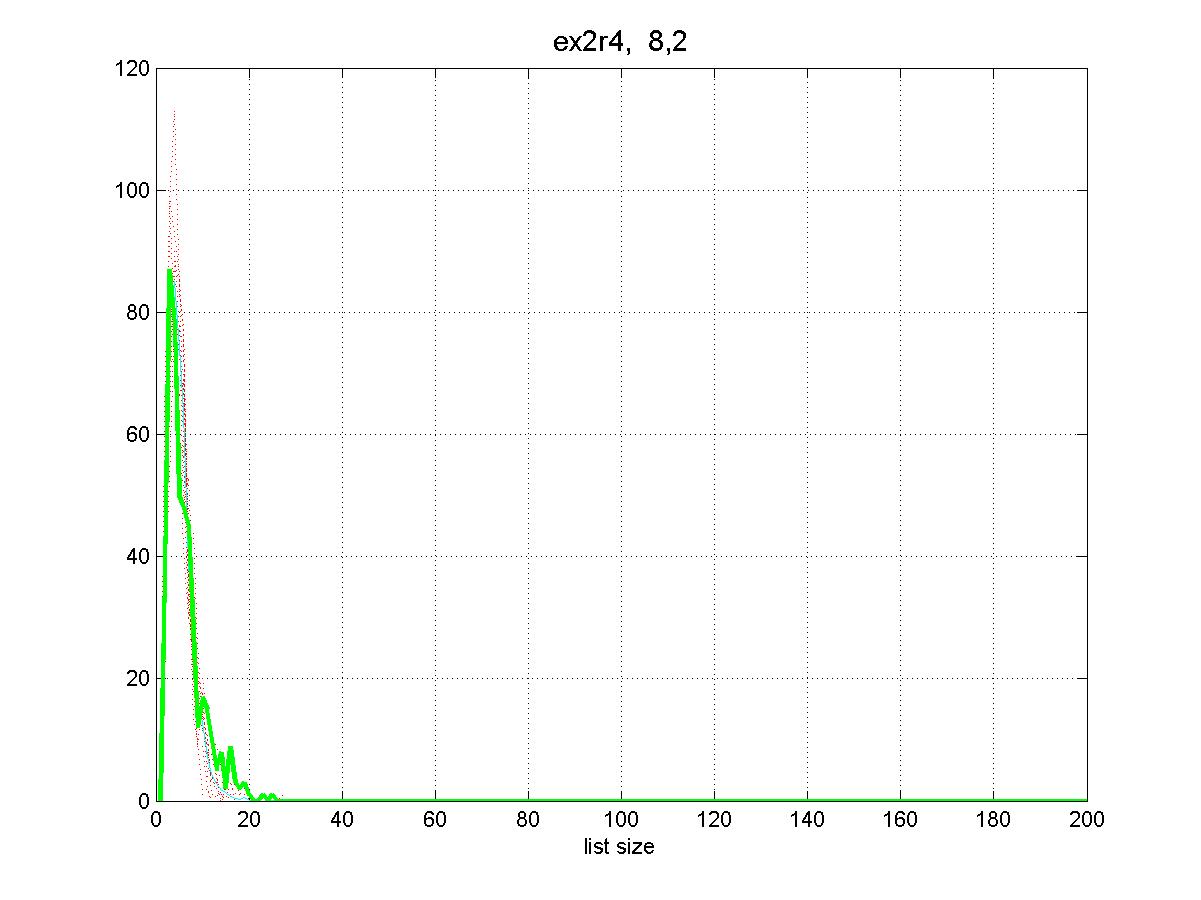

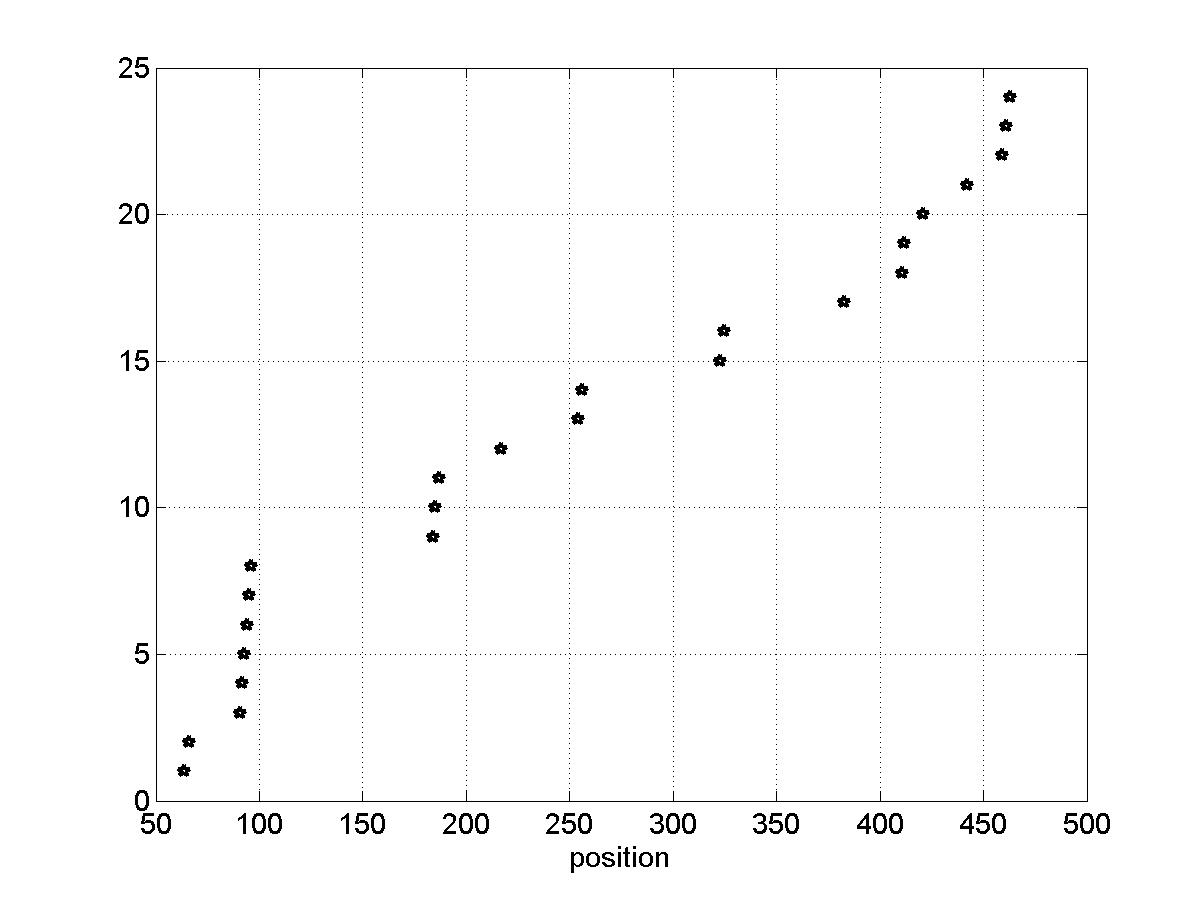


**Figure S28: Similar word distribution and spatial clustering for exon 2r4; (m,mim)=(8,2).**


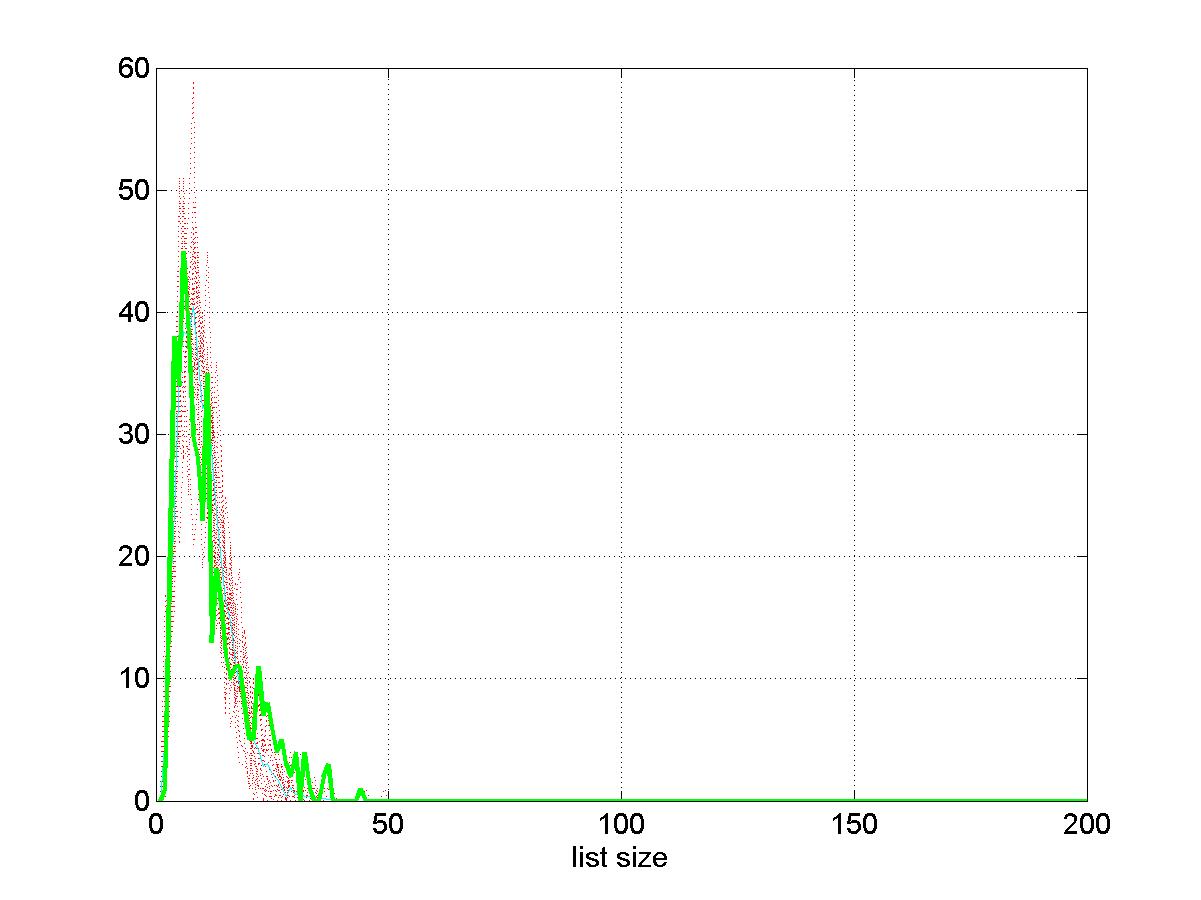

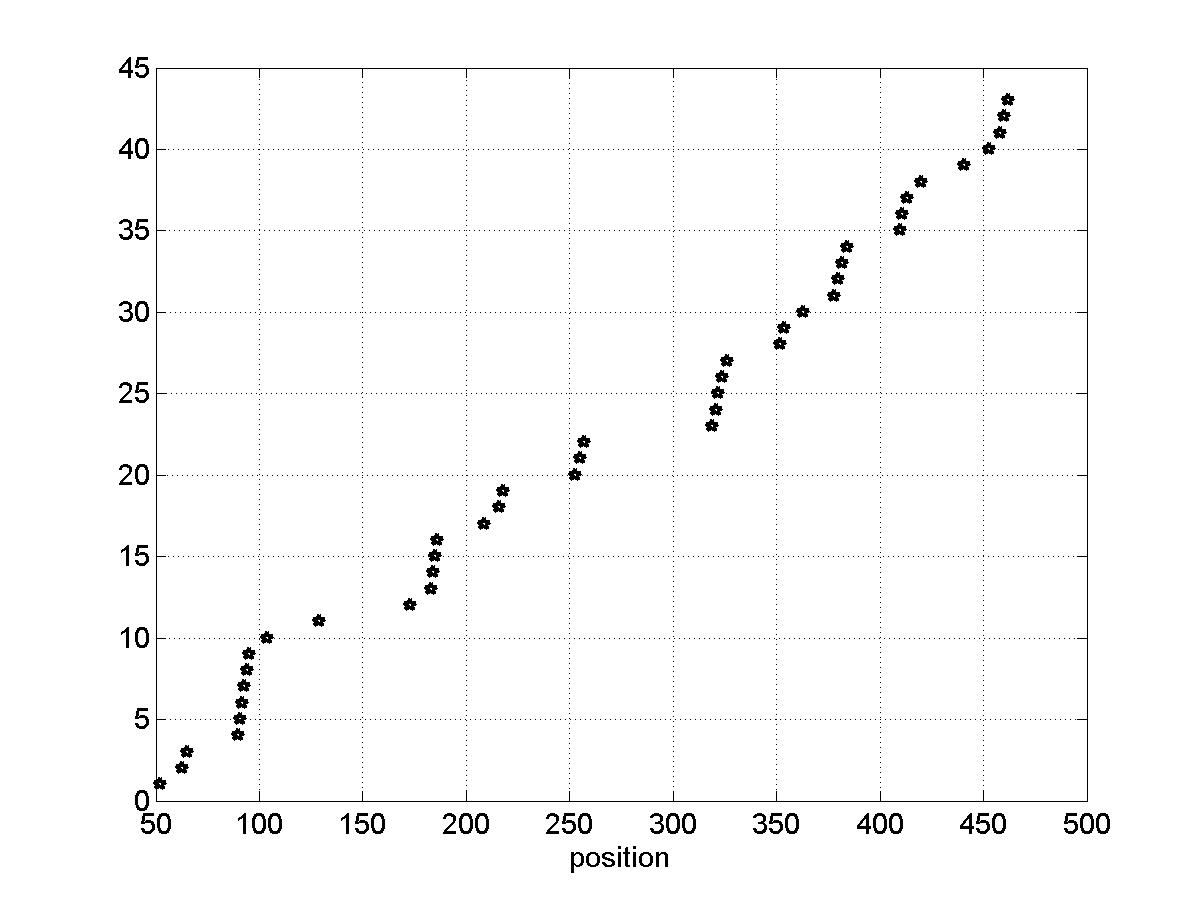


**Figure S29: Similar word distribution and spatial clustering for exon 2r4; (m,mim)=(9,3).**


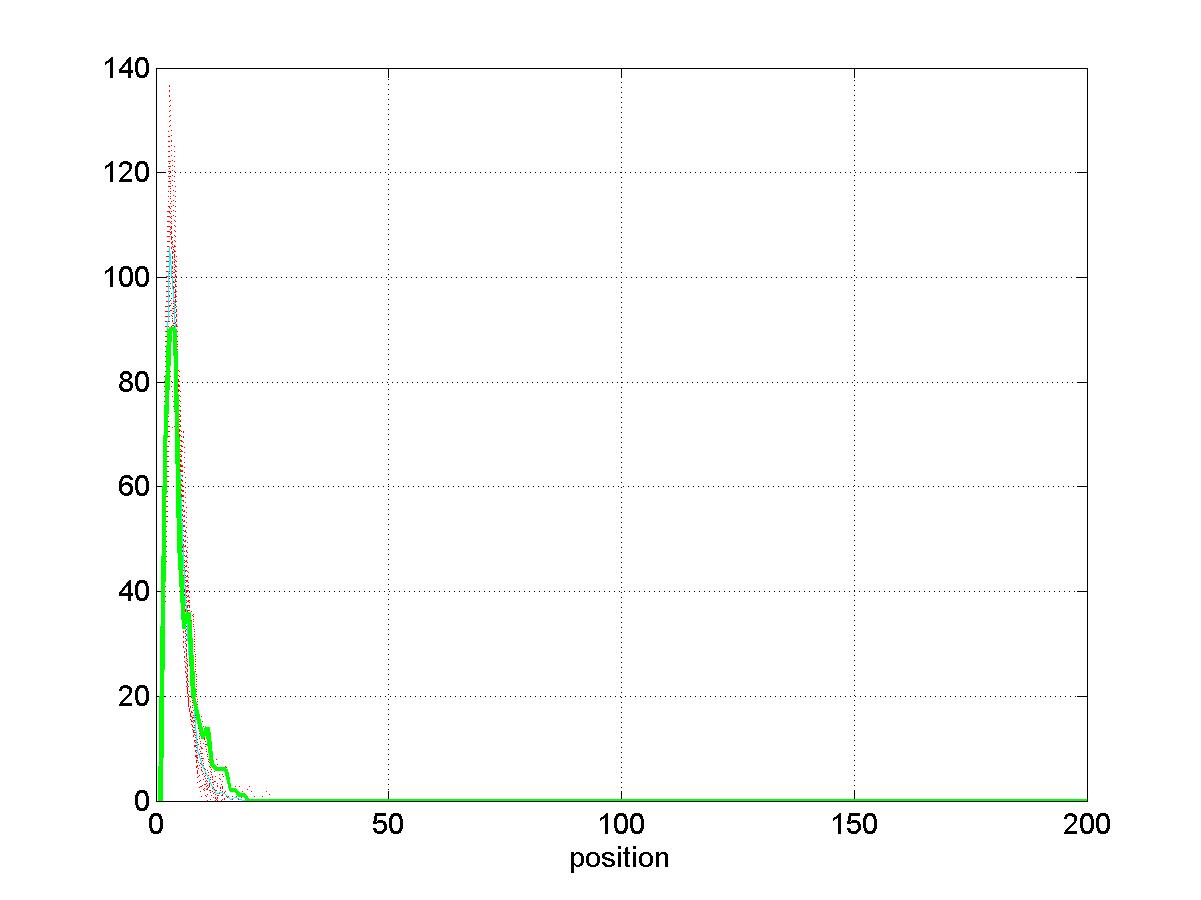

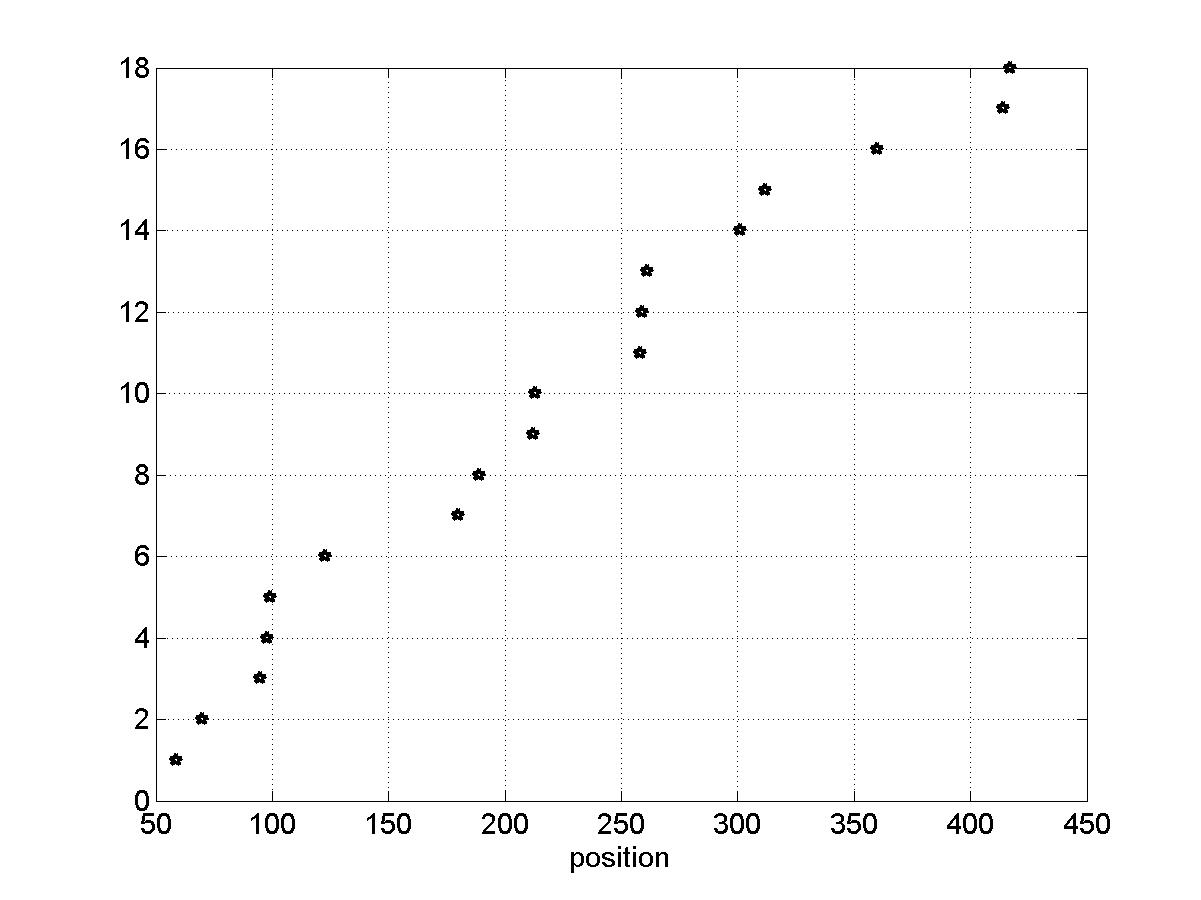


**Figure S30: Similar word distribution and spatial clustering for exon 2r4; (m,mim)=(12,4).**
